# Alkylation of lithiated dimethyl tartrate acetonide with unactivated alkyl halides and application to an asymmetric synthesis of the 2,8-dioxabicyclo[3.2.1]octane core of squalestatins/zaragozic acids

**DOI:** 10.3762/bjoc.15.116

**Published:** 2019-05-31

**Authors:** Herman O Sintim, Hamad H Al Mamari, Hasanain A A Almohseni, Younes Fegheh-Hassanpour, David M Hodgson

**Affiliations:** 1 Department of Chemistry, Chemistry Research Laboratory, University of Oxford, Mansfield Road, Oxford OX1 3TA, United Kingdom; 2 Department of Chemistry, Purdue University, West Lafayette, IN 47907− 2112, USA; 3 Department of Chemistry, College of Science, Sultan Qaboos University, PO Box 36, Al Khoud 123, Muscat, Sultanate of Oman; 4 permanent address: University of Kufa, Najaf Governorate, Iraq

**Keywords:** alkylation, cycloaddition, diazoester, epimerisation, tartaric acid

## Abstract

(*R*,*R*)-Dimethyl tartrate acetonide **7** in THF/HMPA undergoes deprotonation with LDA and reaction at −78 °C during 12–72 h with a range of alkyl halides, including non-activated substrates, to give single diastereomers (at the acetonide) of monoalkylated tartrates **17**, **24**, **33a–f**, **38a,b**, **41** of *R,R*-configuration, i.e., a stereoretentive process (13–78% yields). Separable *trans-*dialkylated tartrates **34a–f** can be co-produced in small amounts (9–14%) under these conditions, and likely arise from the achiral dienolate **36** of tartrate **7**. Enolate oxidation and acetonide removal from γ-silyloxyalkyl iodide-derived alkylated tartrates **17** and **24** give ketones **21** and **26** and then Bamford–Stevens-derived diazoesters **23** and **27**, respectively. Only triethylsilyl-protected diazoester **27** proved viable to deliver a diazoketone **28**. The latter underwent stereoselective carbonyl ylide formation–cycloaddition with methyl glyoxylate and acid-catalysed rearrangement of the resulting cycloadduct **29**, to give the 3,4,5-tricarboxylate-2,8-dioxabicyclo[3.2.1]octane core **31** of squalestatins/zaragozic acids. Furthermore, monoalkylated tartrates **33a**,**d**,**f**, and **38a** on reaction with NaOMe in MeOH at reflux favour (≈75:25) the *cis*-diester epimers *epi-***33a**,**d**,**f** and *epi-***38a** (54–67% isolated yields), possessing the *R,S-*configuration found in several monoalkylated tartaric acid motif-containing natural products.

## Introduction

**Figure 1:**
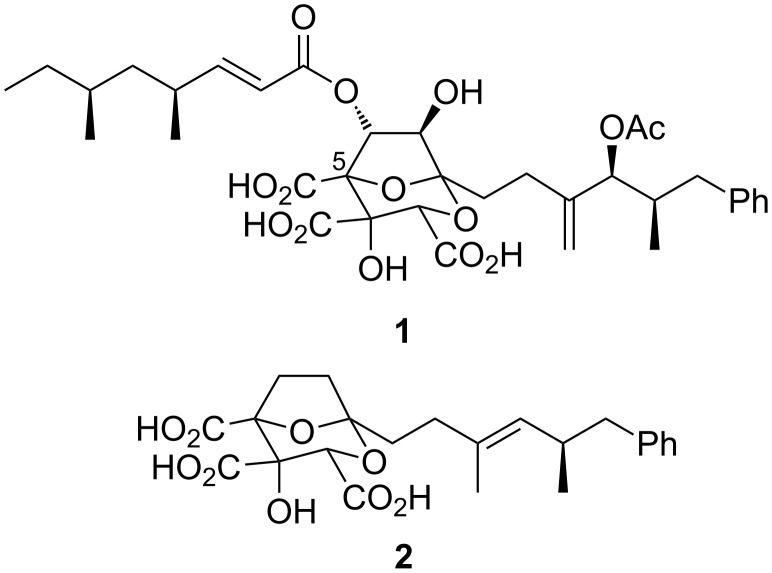
Squalestatin S1/zaragozic acid A (**1**) and DDSQ (**2**).

Since their isolation was reported in the early 1990s [1–2], the squalestatins/zaragozic acids (e.g., squalestatin S1/zaragozic acid A (**1**), Figure 1) have been of enduring interest to synthetic chemists, due to a combination of a synthetically challenging densely functionalised 2,8-dioxabicyclo[3.2.1]octane core [3–6], combined with an increasing range of intriguing biological activities [7–11]. Here, we report in detail the evolution of chemistry that provides an asymmetric entry to the tricarboxylate core of these natural products, with particular focus on tartrate alkylation methodology to establish the fully-substituted C-5 stereocentre (squalestatin numbering).

**Scheme 1:**
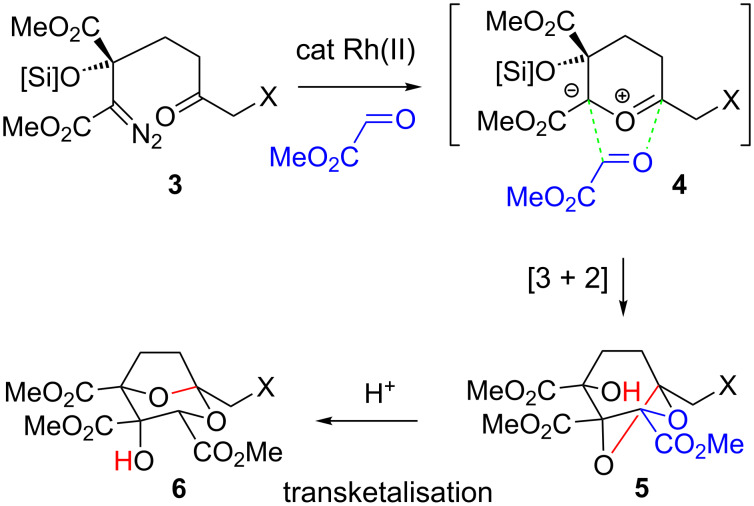
Carbonyl ylide cycloaddition–rearrangement to the squalestatin core [12–13].

Our studies in this area have recently culminated in two communicated syntheses of 6,7-dideoxysqualestatin H5 (DDSQ (**2**), Figure 1) [12–13]. The centrepiece of both of these strategies is a rhodium(II)-catalysed tandem carbon ylide formation from a diazoketone **3** (Scheme 1) and stereoselective [3 + 2] cycloaddition with a glyoxylate (**3** → **4** → **5**) [14–15], followed by an acid-catalysed rearrangement to generate the desired dideoxysqualestatin core **6** with the requisite tricarboxylate functionality installed. While we had earlier established the viability of this approach in a racemic model study (X = H) [14], extension to an asymmetric variant of our aldol route (α-diazoacetate ester anion addition to an α-ketoester) to the cycloaddition substrate **3** (X = H) did not appear promising [16].

**Scheme 2:**
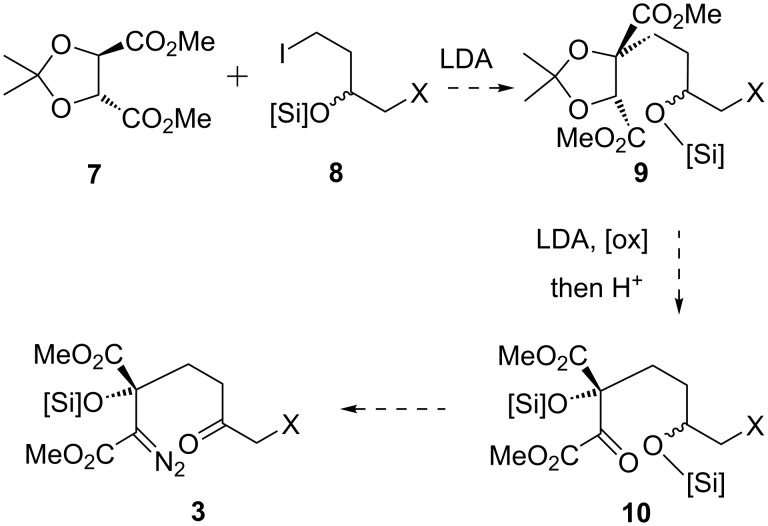
Tartrate alkylation strategy to cycloaddition substrate.

An alternative and asymmetric route to such substrates, ultimately successful, built on stereoselective alkylation of enolates of tartrates (e.g., **7**, Scheme 2) was originally reported by Seebach and co-workers for ‘activated’ (allylic, benzylic) alkyl halides [17–19]. If an alkylated tartrate **9** could be accessed from a silyloxy-substituted alkyl iodide **8** and subsequently oxidised (for example via a second tartrate enolate) with acetonide removal, this would give an α-ketoester **10**. The latter should in principle be a progenitor to the desired α-diazo ester **3**, following condensation with tosylhydrazide, then Bamford–Stevens-type base-induced sulfinate elimination [20] and oxidation of the secondary silyl ether.

## Results and Discussion

**Scheme 3:**
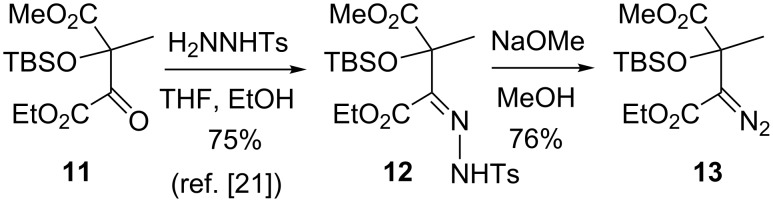
Conversion of α-ketoester to α-diazoester.

The general viability of the α-ketoester to α-diazoester functional group interconversion envisaged in Scheme 2 (**10** → **3**) was readily established on a simpler but closely structurally-related system (Scheme 3). Thus, the known *Z*-hydrazone **12**, previously prepared by us from α-ketoester **11** in 75% yield [21], gave α-diazo ester **13** in 76% yield following reaction with NaOMe. Furthermore, our earlier racemic model study had established that deprotection and oxidation of a secondary silyl ether in the presence of α-diazo ester functionality was feasible, which constitutes precedent for the generation of the ketone functionality in **3** [14]. These observations led us to examine the possibility of substrate assembly through Seebach’s tartrate alkylation methodology.

**Scheme 4:**
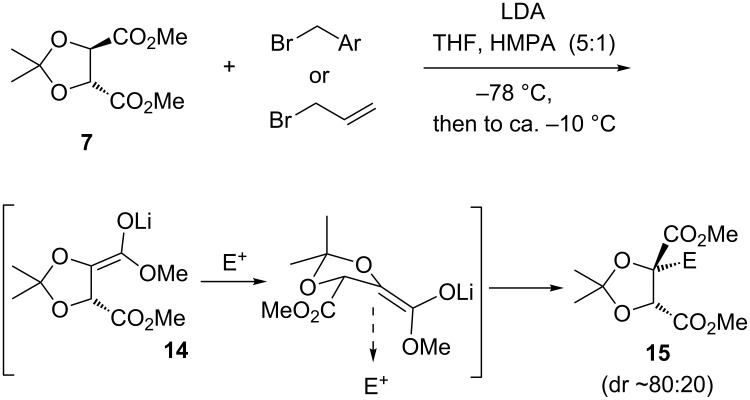
Seebach’s tartrate alkylation and rationalisation of stereoselectivity [17–19].

In 1981, Seebach and Naef communicated that (*R*,*R*)-tartrate acetonide **7** could be deprotonated and undergo stereoselective alkylation (≈80:20 dr) with reactive organohalides (Scheme 4) [17]. The process was valuable, because it allowed direct elaboration of a chiral pool building block that was readily available as either antipode [22–23], with the major alkylated diastereomer **15** being generated in 97:3 er [18]. The study was also notable in showing that the intermediate ester enolate **14** possessed sufficient stability not to undergo significant β-elimination under conditions of its generation and its alkylation: slow addition of pre-cooled LDA (−70 °C) to a mixture of the acetonide and electrophile in THF/HMPA at −78 °C, followed by slow warming to ≈−10 °C before work-up. Finally, the reaction displayed remarkable stereoselectivity, in that the electrophile was introduced on ostensibly the more hindered face of the enolate (that is, *cis* (“contrasteric”) [24] to the unenolised ester group). The former observation was rationalised due to the enolate π-system and potentially cleavable beta σ-C–O bond lying mutually orthogonal, while the latter was subsequently ascribed to alkylation occurring from an envelope conformation wherein the unenolised ester resided pseudoequatorial to avoid 1,3-steric interactions with a pseudoaxial methyl of the *gem*-dimethyl group [18,24]; it was proposed that the axial methyl group directed electrophile incorporation away from itself (Scheme 4).

**Scheme 5:**
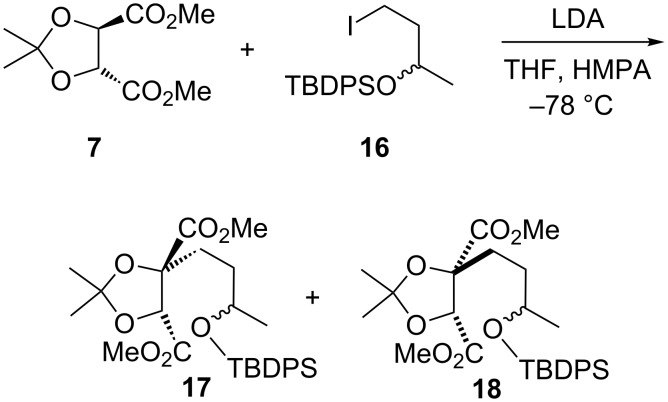
Tartrate alkylation with a non-activated alkyl iodide.

The fragile nature of the lithium ester enolate of dimethyl tartrate acetonide (to β-elimination with loss of acetone) was evident from Seebach’s work, which concluded that only especially reactive halides (methyl, benzylic, allylic) were feasible electrophiles; with iodoethane, 1-iodo-2-methylpropane and chloromethoxymethane no alkylation products were formed [17–19]. Given these rather discouraging observations in the context of our proposed chemistry (Scheme 2), we were pleased to find that initial studies with (*R*,*R*)-tartrate **7** and a 3-silyloxy-1-iodobutane **16** did generate alkylated tartrates (Scheme 5). Following Seebach’s protocol, with warming overnight to room temperature, gave a 50% yield (90% based on recovered iodide **16**) of a separable 76:24 mixture of alkylated tartrates **17** and **18**, respectively. The relative stereochemistry was assigned by analogy with Seebach’s findings for substituted tartrates: that the diastereomer with the ring methine *cis* to the ester group (i.e., **17**) always displays the higher chemical shift (≈5 ppm vs ≈4.5 ppm in CDCl_3_) [18], and was further supported by 1D NOESY experiments on both alkylated tartrates **17** and **18**. The use of DMPU as co-solvent [18,25] reversed the ratio, with the currently undesired diastereomer **18** becoming favoured (37:63, **17**:**18**). However, with HMPA the proportion of **17** improved significantly (>90:10, **17**:**18**) if the reaction was maintained at −78 °C for several hours before quenching at that temperature, giving isolated yields of 30–50% for **17**. The absolute configuration of **17** was based on the chemical correlation studies of Seebach [17–19] and of Pan [26] for benzylations, and subsequently those of Nagano [27] and of Li [28] for allylations, i.e., stereoretentive (contrasteric) alkylation. As noted above, Seebach recorded 97:3 er (by NMR using a chiral shift reagent) for a benzylated tartrate [18]; in the present case, chiral HPLC comparison of **17** with the corresponding adduct from *S*,*S*-tartrate (*ent*)-**7** indicated they were both of >98:2 enantiopurity in the tartrate portion.

**Scheme 6:**
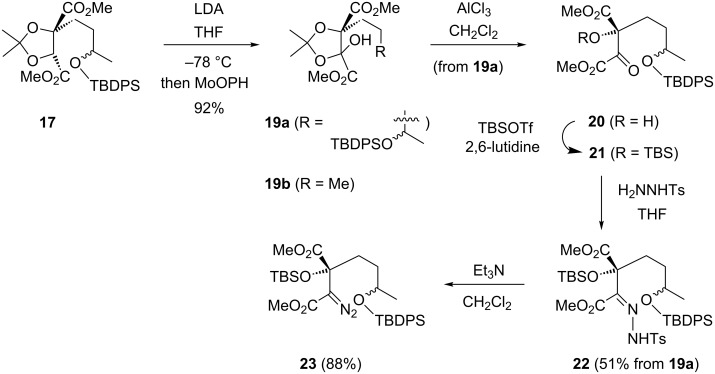
Alkylated tartrate to diazoester sequence.

Before exploring the scope of the alkylation chemistry further, it was considered prudent for the proposed asymmetric approach to the dideoxysqualestatin core **6** (Scheme 1 and Scheme 2) to establish the viability of the rest of sequence outlined in Scheme 2 from an alkylated tartrate. While further C–C bond formation by enolate formation at the remaining methine on a monoalkylated tartrate acetonide had been reported by Molander and Harris [29], and by Kelly and co-workers [30]; the question whether such an enolate could be oxidised required investigation. Although the reaction of alkylated tartrate **17** with NaHMDS/2-(phenylsulfonyl)-3-phenyloxaziridine [31] gave an unidentifiable mixture, the use of LDA and MoOPH [32–33] at −78 °C followed by warming to −50 °C for 3 h gave the hydroxy acetonide **19a** (Scheme 6) in 92% yield as a mixture of 4 diastereomers. Similarly, the enolate of the simpler propylated tartrate **33a** [12] reacted with MoOPH to give the analogous hydroxy acetonide **19b** in 96% yield (3:1 dr); if the MoOPH was added to the enolate which had been warmed to −40 °C, a more typical hydroxylation temperature, then a reduced yield of **19b** was observed (53%). Indirect hydroxylation of the propylated tartrate enolate was also attempted using CBr_4_ (at −78 °C) as a more readily available/convenient electrophile, which also gave the hydroxy acetonide **19b** presumably by way of hydrolysis on work-up of an intermediate bromo acetonide, albeit in significantly reduced yield (33%).

In contrast to a simple hydroxy acetonide (formally derived from an α-hydroxy aldehyde and acetone) [34], hydroxy acetonide **19a** was found stable to mild bases such as Et_3_N and iPr_2_NH, whereas the use of NaH or NaHMDS in THF both decomposed **19a** into unidentifiable polar products. Attempted acid-induced loss of acetone with PTSA in MeOH, PPTS in refluxing MeOH, or 80% AcOH at reflux [27] all led to quantitative recovery of hydroxy acetonide **19a**, whereas 5% aq HCl resulted in acetonide removal and concomitant desilylation. Initial Lewis acids screened either failed to react (PdCl_2_(MeCN)_2_), or led to complex mixtures (BF_3_, YbOTf, TBSOTf). More encouragingly, both AlCl_3_ and FeCl_3_ were found to cleave the acetonide **19a** at rt, with the TBDPS group only being partially lost (≈15%) in both cases. AlCl_3_ was observed to deprotect the TBDPS ether more slowly than FeCl_3_, and adjustment of the reaction conditions with AlCl_3_ (to 2 equiv in CH_2_Cl_2_, −78 °C, followed by slow warming to −50 °C) cleanly provided α-ketoester **20** (Scheme 6). α-Ketoesters can be prone to hydrate easily (2D TLC analysis of **20** indicated decomposition); therefore, **20** and the derived tertiary TBS ether **21** were carried on directly to form hydrazone **22** (51% yield over 3 steps from hydroxy acetonide **19a**). Unlike with hydrazone **12** (Scheme 3), application of NaOMe was not conducive to effective diazo formation from hydrazone **22**, giving a mixture of unidentified products; however, hydrazone **22** was cleanly converted into α-diazo ester **23** (88%) using Et_3_N [35–36].

**Scheme 7:**
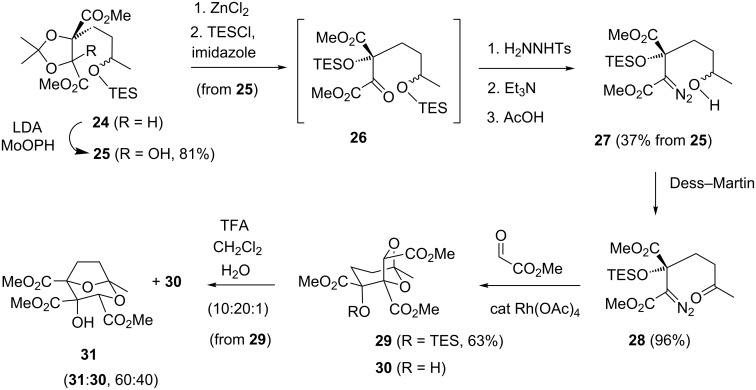
TES protection approach to the squalestatin core.

TBDPS protection for the secondary alcohol had originally been selected principally for its likely tolerance to potential (hydroxy) acetonide removal conditions, and with the possibility [37–38] of its selective deprotection in α-diazo ester **23** in the presence of the tertiary TBS ether. It was considered important that the tertiary alcohol remain masked during projected oxidation of the released secondary alcohol to give the ketone functionality in the cycloaddition substrate, as otherwise essentially irretrievable five-membered lactol formation would be expected [39]. Unfortunately, various reagents (TBAF/AcOH [40], NaH/HMPA [41], Bu_4_OH/DMF [40], NaOMe/MeOH) failed to selectively deprotect the secondary TBDPS ether in α-diazo ester **23** in the presence of the tertiary TBS ether. Reassessment of the protecting group strategy led us to TES protection at both alcohols, on the basis that this group should be robust enough to withstand the enolate manipulation chemistry, that desilylation of the secondary TES ether during acetonide removal could be restored in the subsequent tertiary alcohol silylation step, that selective 2° over 3° TES ether desilylation should be readily achievable using AcOH [14], and that the remaining tertiary TES ether should be potentially labile enough to be removed under typical transketalisation conditions (TFA/CH_2_Cl_2_/H_2_O (10:20:1), 40 °C, 48–68 h [14], cf, Scheme 1), thereby circumventing the separate prior desilylation step in our earlier racemic model study [14]. In the event, application of this TES protection approach did provide access to diazo alcohol **27** (Scheme 7). As anticipated from the above deprotection studies with TBDPS ether **23**, 1% aq HCl removed both the acetonide and TES groups in hydroxy acetonide **25**. Subsequent silylation using TESOTf gave the bis-TES ketone **26**, which was not purified but taken on through diazo formation and desilylation to give diazo alcohol **27** (17% from **25**). The efficiency of the sequence from hydroxy acetonide **25** to diazo alcohol **27** could be improved (to 37%) using ZnCl_2_ for the initial deprotection and TESCl in the silylation; the latter minimises formation of the undesired silylated six-membered lactol form of **26**. The remaining steps to the model core **31** (Scheme 7) closely mirrored our previous racemic synthesis of **31** (from the corresponding 3° TBS ether) [14], and this strategy was subsequently also successfully applied in our most recent total synthesis of DDSQ (**2**) [13].

**Scheme 8:**
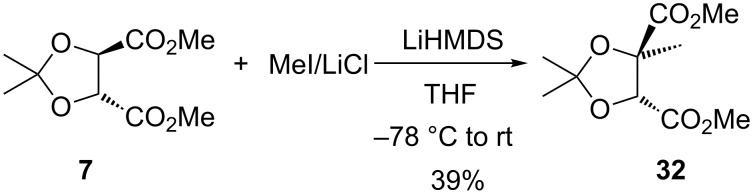
Tartrate acetonide methylation.

We now returned to study the Seebach alkylation chemistry in more detail. In 2008, Lipton and co-workers had observed that the cyclopentylidene derivative of diethyl tartrate reacted with LiHMDS in the presence of LiCl and MeI, to give exclusively the corresponding *trans*-monomethylated tartrate in 72% yield [42]; this compares with methylation of acetonide tartrate **7** by Seebach, which was reported to give an inseparable mixture of monomethylated product (86:14 dr), dimethylated, and recovered **7** (50%, 79:15:6, respectively) [17–19]. Application of Lipton's conditions to tartrate **7**, cleanly gave the *trans*-monomethylated product **32**, albeit in moderate yield (39%, Scheme 8); however, extension to a higher alkylating agent (PrI) was unsuccessful, returning only ≈25% of tartrate **7** in an impure state.

**Scheme 9:**
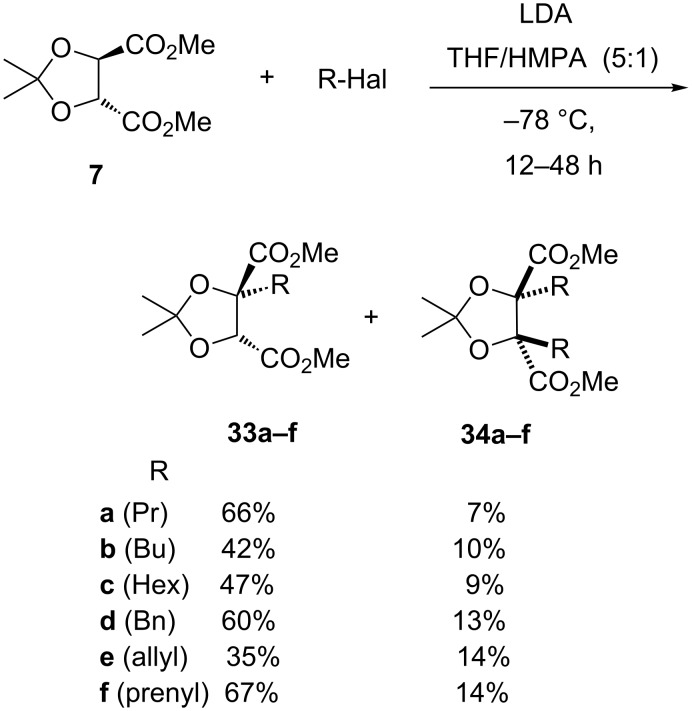
Tartrate alkylation with various alkyl halides.

In contrast to the unsuccessful propylation with LiHMDS/LiCl mentioned above, following our modified Seebach‘s protocol, propylation could be achieved to give propylated tartrate **33a** [12] (Scheme 9), in 66% yield and 97:3 er by chiral HPLC, with the *trans*-dipropylated product **34a** also being separately isolated, in 7% yield. Other primary ‘non-activated’ alkyl iodides also led to alkylated tartrates **33b**,**c** and the corresponding dialkylated side-products **34b**,**c** (Scheme 9). Under our modified alkylation conditions, ‘activated’ bromides (benzyl, allyl and prenyl), previously examined by Seebach [17–19], all gave the corresponding monoalkylated tartrates **33d–f** also as single diastereomers (along with separable *trans*-dialkylated material **34d–f**, Scheme 9). These latter results indicate that it is the modified reaction conditions, rather than the nature of the alkylating agent, which leads to the improved diastereoselectivity [43]. The *trans* stereochemistry assignment for the dialkylated products **34** follow from the observed equivalence of the acetonide methyl groups in all their proton and carbon NMR spectra.

**Scheme 10:**
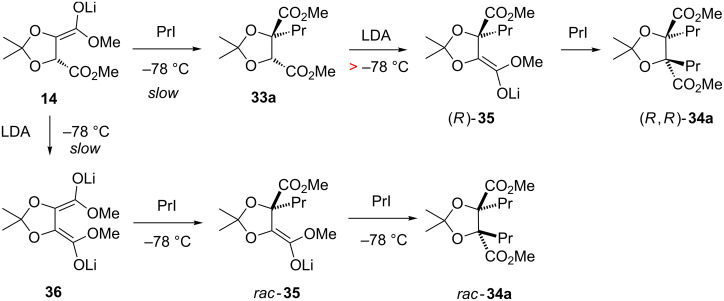
Rationalisation of dialkylation observations.

In Seebach's original studies, which established retention on tartrate alkylation for the major diastereomer (formed, as noted above, in 97:3 er), a further intriguing observation was made: that the minor diastereomer was obtained in virtually racemic form (determined by NMR using a chiral shift reagent) [18–19]. It was suggested that the latter principally arose from alkylation (then protonation, by unreacted **7**) [19] of the (achiral) dienolate **36** (Scheme 10) of **7**. In the current work, where the major difference is prolonged reaction time at low temperature and no warming before quenching, the minor diastereomer is not observed – but dialkylated byproduct **34** is. An attempt to propylate the monopropylated tartrate **33a** under our conditions mainly returned starting **33a** (68%), with only a trace of *trans*-dipropylated product **34a** being isolated (2%). Seebach’s observations, together with ours, indicate that the dienolate **36** does form to some extent at −78 °C (Scheme 10) [44–46], and both it and the derived alkylated mono-anion *rac*-**35** are quite reactive to alkylation, but protonation of the alkylated mono-anion *rac*-**35** (by unreacted **7**) does not occur at that temperature; also, once monoalkylation has occurred from the mono-enolate **14**, then **33a** is not readily deprotonated at −78 °C. Clearly, monoalkylated tartrate can be deprotonated and trapped with electrophiles if the system is warmed above −78 °C: to −30 °C by Molander and Harris [29], whereas −50 °C was sufficient for the enolate oxidation steps in Scheme 6 and Scheme 7. Indeed, propylation of the monopropylated tartrate **33a** can be achieved in 34% yield, if the reaction mixture is warmed to −50 °C (Scheme 10). Chiral HPLC comparison of the *trans*-dipropylated material **34a** obtained as the byproduct from the alkylation of tartrate acetonide **7**, and from propylation of the monopropylated tartrate **33a**, confirmed that the former was essentially racemic (52:48 er), while the latter was 98:2 er. Therefore, if one wishes to generate (*R*,*R*)- (or (*S*,*S*)-) *C*2-symmetric dialkylated tartrates [47–49], an important conclusion from the above observations and analysis is that monoalkylation should be carried out first, and the isolated monoalkylated material then separately subjected to a second alkylation, allowing warming to ≈−40 °C.

**Scheme 11:**
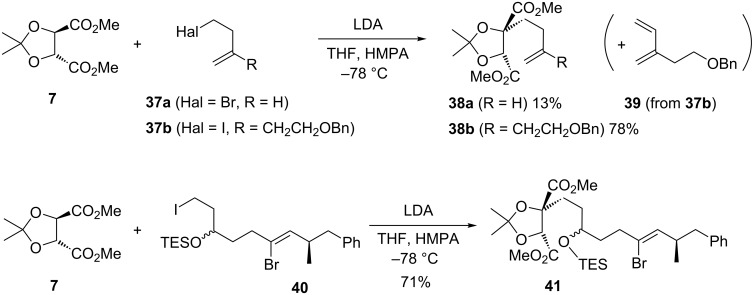
Tartrate alkylation chemistry with more complex alkyl iodides [12–13].

Our first synthesis of 6,7-dideoxysqualestatin H5 (DDSQ (**2**), Figure 1), required extension of the above tartrate alkylation chemistry to a homoallylic halide as the electrophile (Scheme 11) [12]. An initial experiment with 4-bromobut-1-ene (**37a**) was not encouraging, delivering the homo-allylated product **38a** in only 13% yield. It was suspected that competing elimination (to give butadiene) contributed to the low yield. Support for this was found with the real system, where the corresponding diene **39** was isolated in up to 36% yield. In our earlier studies, typically approximately equimolar quantities of tartrate and alkylating agent were used, but with the halide now being synthetically more valuable, efforts focused on conditions which gave the best yields using it as the limiting agent. A slight excess of tartrate relative to iodide produce the homo-allylated tartrate **38b** in 34% yield, but this could be improved to 60–78% by using 100% excess of tartrate [12]. It was also noted that minimal contact time between the sensitive iodide **37b** and HMPA at low temperature before slow addition of the pre-cooled LDA minimised diene formation, with the excess residual unreacted tartrate being most conveniently removed by distillation on large-scale. Our second synthesis of DDSQ (**2**) introduced the full side-chain through alkylation with iodide **40** to give the alkylated tartrate **41** in 71% yield, and used only a slight excess of tartrate **7** and LDA (1.2 and 1.5 equiv, respectively) for 72 h at −78 °C [13]. These examples demonstrate the viability of the tartrate alkylation chemistry with more complex and valuable electrophiles.

**Figure 2:**
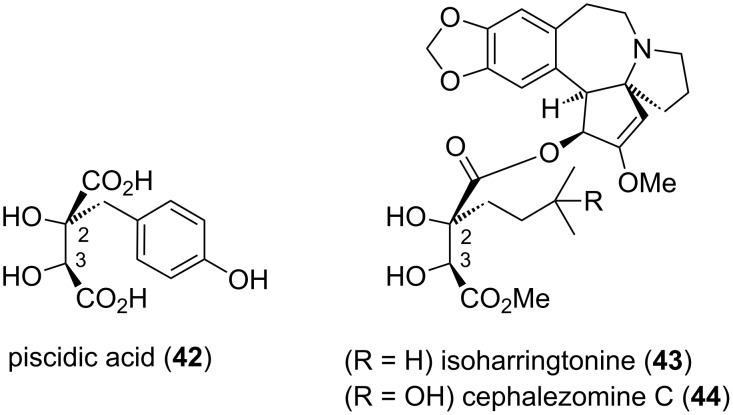
Natural product examples containing the monoalkylated tartaric acid motif.

While monoalkylated tartrate acetonides were applied in squalestatin syntheses as highlighted above, the monoalkylated tartaric acid motif is also directly present in several natural products, such as hydroxybenzyl-substituted piscidic acid (**42**) and congeners (fukiic and cimicifugic acids) [50], and the Cephalotaxus alkaloids isoharringtonine (**43**) and cephalezomine C (**44**) [51]. In these latter natural products, the monoalkylated tartaric acid residues typically possess 2*R*,3*S* stereochemistry (Figure 2); one exception is cephalezomine D [51], which is the 3*R*-epimer of cephalezomine C (**44**). Since the chiral (*R*,*R*- *S*,*S*-) tartrate acetonides undergo stereoretentive alkylation, then direct access to the stereochemistry present in the majority of these natural products is not possible. Nevertheless, a couple of isolated examples in the Chinese chemical literature from the late 1980s indicated that subsequent base-induced epimerisation of monoalkylated (*p*-(benzyloxy)benzylated and prenylated) tartrate acetonides favours *R*,*S* stereochemistry, providing access to piscidic acid (**42**) [26] and the tartrate residue of isoharringtonine (**43**) [28].

**Scheme 12:**
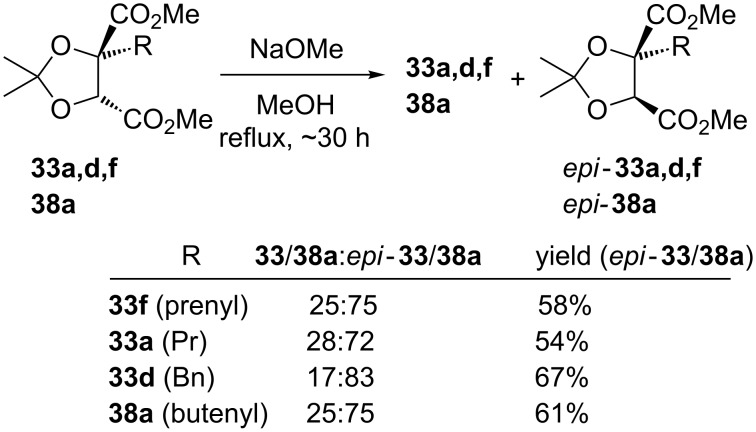
Epimerisation of monoalkylated tartrates.

We studied the previously reported epimerisation of prenylated tartrate **33f**, on 0.5 mmol scale ([28] 5 mmol), using NaOMe (1 equiv) in MeOH (0.5 M) at room temperature for 12 h. Under these conditions we observed only partial epimerisation (**33f**/*epi-***33f** 75:25, [[28] 12:88]), but a 25:75 ratio in favour of *epi-***33f** could be achieved in MeOH (0.06 M) at reflux for 30 h (Scheme 12). That equilibrium had been reached was established by subjecting *epi-***33f** to these latter reaction conditions, which returned the same 25:75 ratio of **33f**/*epi-***33f**. Other monoalkylated tartrates were found to give similar levels of epimerisation (Scheme 12), generating the chromatographically separable epimerised tartrates in 54–67% isolated yields.

## Conclusion

Contrary to the seminal observations of Seebach and Naef, we have demonstrated that lithiated dimethyl tartrate acetonide can undergo stereoretentive alkylation even with “non-activated” alkyl halides, in synthetically useful yields and high er. Optimal reaction conditions are prolonged reaction time at −78 °C, followed by quenching at that temperature; these conditions also avoid cogeneration of the (racemic) diastereomer side-product originally observed on warming. Essentially racemic dialkylated tartrate is observed as a minor side-product under our modified conditions. The reaction pathways that lead to these racemic products under the different reaction conditions have been rationalised. Application of this methodology to generate the 6,8-dioxabicyclo[3.2.1]octane core of the squalestatins/zaragozic acids in an asymmetric fashion, is shown to evolve from a carefully orchestrated sequence of oxidation and judicious protecting group manipulation. Base-induced epimerisation of the monoalkylated tartrates favours *cis*-disposition of the ester groups on the five-membered ring, thereby accessing the predominant stereochemistry found in several substituted tartaric acid-containing natural products. Our findings on tartrate alkylation with non-activated alkyl halides, on origins of side-products, and on conditions for epimerisation of the monoalkylated tartrates, significantly broaden the scope and understanding of Seebach’s alkylation chemistry.

## Supporting Information

File 1 Experimental procedures, characterisation data and ^1^H and ^13^C NMR spectra for all new compounds.

## References

[1] Dawson M J, Farthing J E, Marshall P S, Middleton R F, O’Neill M J, Shuttleworth A, Stylli C, Tait R M, Taylor P M, Wildman H G (1992). J Antibiot.

[2] Dufresne C, Wilson K E, Zink D, Smith J, Bergstrom J D, Kurtz M, Rew D, Nallin M, Jenkins R, Bartizal K (1992). Tetrahedron.

[3] Armstrong A, Blench T J (2002). Tetrahedron.

[4] Nicewicz D A, Satterfield A D, Schmitt D C, Johnson J S (2008). J Am Chem Soc.

[5] Wang Y, Metz P (2011). Chem – Eur J.

[6] Kawamata T, Nagatomo M, Inoue M (2017). J Am Chem Soc.

[7] Liu C-I, Jeng W-Y, Chang W-J, Ko T-P, Wang A H-J (2012). J Biol Chem.

[8] Guan Z, Wen R, Lam B I (2014). Compositions and Methods for the Diagnosis and Treatment of Dolichol Deficiency Related Disorders. PCT Int. Appl..

[9] Gabriel H B, Silva M F, Kimura E A, Wunderlich G, Katzin A M, Azevedo M F (2015). Antimicrob Agents Chemother.

[10] Saito K, Shirasago Y, Suzuki T, Aizaki H, Hanada K, Wakita T, Nishijima M, Fukasawa M (2015). J Virol.

[11] Lanterna C, Musumeci A, Raccosta L, Corna G, Moresco M, Maggioni D, Fontana R, Doglioni C, Bordignon C, Traversari C (2016). Cancer Immunol Immunother.

[12] Fegheh-Hassanpour Y, Arif T, Sintim H O, Al Mamari H H, Hodgson D M (2017). Org Lett.

[13] Almohseni H A A, Al Mamari H H, Valade A, Sintim H O, Hodgson D M (2018). Chem Commun.

[14] Hodgson D M, Villalonga-Barber C, Goodman J M, Pellegrinet S C (2010). Org Biomol Chem.

[15] Hodgson D M, Labande A H, Muthusamy S (2013). Org React.

[16] Wang F, Liu X, Zhang Y, Lin L, Feng X (2009). Chem Commun.

[17] Naef R, Seebach D (1981). Angew Chem.

[18] Seebach D, Aebi J D, Gander-Coquoz M, Naef R (1987). Helv Chim Acta.

[R19] 19Naef, R. Chiral enolates, Dissertation No. 7442, ETH Zürich, Switzerland, 1983.

[20] Regitz M, Maas G (1986). The Bamford-Stevens Reaction. Diazo Compounds: Properties and Synthesis.

[21] Fegheh-Hassanpour Y, Ebrahim F, Arif T, Sintim H O, Claridge T D W, Amin N T, Hodgson D M (2018). Org Biomol Chem.

[22] Gawronski J, Gawronska K (1999). Tartaric and Malic Acids in Synthesis.

[23] Ghosh A K, Koltun E S, Bilcer G (2001). Synthesis.

[24] Ladner W (1982). Angew Chem.

[25] Mukhopadhyay T, Seebach D (1982). Helv Chim Acta.

[26] Nie X, Wang Q, Li Y, Pan X (1987). Gaodeng Xuexiao Huaxue Xuebao.

[27] Tokunaga Y, Nagano H, Shiota M (1986). J Chem Soc, Perkin Trans 1.

[28] Zhang G-L, Li S-B, Li Y-L (1989). Acta Chim Sin (Chin Ed).

[29] Molander G A, Harris C R (1996). J Am Chem Soc.

[30] Kelly T R, Cai X, Tu B, Elliott E L, Grossmann G, Laurent P (2004). Org Lett.

[31] Evans D A, Trotter B W, Barrow J C (1997). Tetrahedron.

[32] Vedejs E, Engler D A, Telschow J E (1978). J Org Chem.

[33] Vedejs E, Larsen S (1990). Org Synth, Coll Vol VII.

[34] Jarosz S, Skóra S, Szewczyk K (2000). Tetrahedron: Asymmetry.

[35] House H O, Blankley C J (1968). J Org Chem.

[36] Blankley C J, Sauter F J, House H O (1973). Org Synth, Coll Vol V.

[37] Crouch R D (2013). Tetrahedron.

[38] Wuts P G M (2014). Greene's Protective Groups in Organic Synthesis.

[39] Villalonga–Barber C (2001). Synthetic studies towards the zaragozic acids.

[40] Higashibayashi S, Shinko K, Ishizu T, Hashimoto K, Shirahama H, Nakata M (2000). Synlett.

[41] Shekhani M S, Khan K M, Mahmood K, Mozzam Shah P, Malik S (1990). Tetrahedron Lett.

[42] Ramamoorthy G, Acevedo C M, Alvira E, Lipton M A (2008). Tetrahedron: Asymmetry.

[43] Crich D, Hao X (1999). J Org Chem.

[44] Barros M T, Burke A J, Maycock C D (1999). Tetrahedron Lett.

[45] Barros M T, Burke A J, Lou J-D, Maycock C D, Wahnon J R (2004). J Org Chem.

[46] Burke A J, Maycock C D, Ventura M R (2006). Org Biomol Chem.

[47] Zhu J, Yuan Y, Wang S, Yao Z-J (2017). ACS Omega.

[48] Wang N, Song J, Jang K H, Lee H-S, Li X, Oh K-B, Shin J (2008). J Nat Prod.

[49] Balansa W, Islam R, Fontaine F, Piggott A M, Zhang H, Webb T I, Gilbert D F, Lynch J W, Capon R J (2010). Bioorg Med Chem.

[50] Miranda V, Maycock C D, Ventura M R (2015). Eur J Org Chem.

[51] Morita H, Arisaka M, Yoshida N, Kobayashi J (2000). Tetrahedron.

