# Supplementary material for: Alkylation of lithiated dimethyl tartrate acetonide with unactivated alkyl halides and application to an asymmetric synthesis of the 2,8-dioxabicyclo[3.2.1]octane core of squalestatins/zaragozic acids
Source: Beilstein J Org Chem. 2019 May 31;15:1194–202. doi: 10.3762/bjoc.15.116 (PMC6604716; doi:10.3762/bjoc.15.116)

BEILSTEIN JOURNAL OF ORGANIC CHEMISTRY

Supporting Information

for

Alkylation of lithiated dimethyl tartrate acetonide with unactivated alkyl halides and application to an asymmetric synthesis of the 2,8-dioxabicyclo[3.2.1]octane core of squalestatins/zaragozic acids

Herman O. Sintim, Hamad H. Al Mamari, Hasanain A. A. Almohseni, Younes Fegheh-Hassanpour and David M. Hodgson

Beilstein J. Org. Chem. **2019,** *15,* 1194–1202. doi:10.3762/bjoc.15.116

Experimental procedures, characterisation data and ^{1}H and ^{13}C NMR spectra for all new compounds

License and Terms: This is a supporting information file under the terms of the Creative Commons Attribution License (http://creativecommons.org/licenses/by/4.0). Please note that the reuse, redistribution and reproduction in particular requires that the authors and source are credited.
The license is subject to the *Beilstein Journal of Organic Chemistry* terms and conditions: (https://www.beilstein-journals.org/bjoc)

Experimental

General information

All reactions requiring anhydrous conditions were carried out under an atmosphere of argon in flame-dried glassware. Tetrahydrofuran (THF), dichloromethane (DCM), ether (Et_2O) and ethyl acetate (EtOAc) were obtained from Grubbs' drying stills [1]. MeOH was dried over 4 Å MS for at least 24 h. Light petrol (petroleum ether) 30–40 °C, or petrol (petroleum ether) 40–60 °C was used in column chromatography. The latter was carried out using silica gel (VWR chemicals, BDH), monitored by thin-layer chromatography (TLC) (Merck 60 F_{254}) plates. TLC plates were viewed using ultraviolet light (λ_{max} = 254/365 nm) and immersion in $KMnO_4$, anisaldehyde or vanillin stains, followed by heating. Except where stated otherwise, commercially available reagents were used as received. Melting points (mp) were obtained using an Electrothermal melting point apparatus to the nearest 1 °C and are uncorrected. $[\alpha]_D$ values are given in 10^{-1} deg cm^2 g^{-1}. Concentrations (*c*) are given in g/cm^3. HPLC was performed on a Gilson system with 712 Controller Software (for **17**), Dionex UltiMate 3000 (for **33a**) and Shimadzu Prominence-i LC-2030 (for **34a**). Infrared spectra were obtained using a PerkinElmer FT–IR spectrometer (Universal ATR Sampling Accessory), with absorption maxima quoted in wavenumbers (cm^{-1}). Peak intensities are described as broad (br), weak (w), medium (m) or strong (s). Nuclear magnetic resonance (^1H NMR and ^{13}C NMR) spectra were recorded on Bruker Avance DPX 200, AVIIIHD 400, AVII 500, and AVIIIHD 500 spectrometers in $CDCl_3$ (referenced to residual $CHCl_3$ singlet at δ 7.27 for ^1H NMR spectra, and to the central line of $CDCl_3$ triplet at δ 77.16 for ^{13}C NMR spectra), or in C_6D_6 (referenced to residual C_6H_6 singlet at δ 7.16 for ^1H NMR spectra, and to the central line of C_6D_6

triplet at δ 128.06 for ^{13}C NMR spectra). Chemical shifts are quoted in parts per million (ppm). Coupling constants (*J*) are measured to the nearest 0.5 Hertz (Hz). The splittings are quoted as singlet (s), doublet (d), triplet (t), quartet (q), or multiplet (m). The ^{13}C NMR peaks were assigned by standard methods using HSQC. High-resolution mass spectra were obtained by electrospray ionisation (ESI) or chemical ionisation (CI) using tetraoctylammonium bromide or sodium dodecyl sulfate as the lock mass.

4-Ethyl 1-methyl 2-((*tert*-butyldimethylsilyl)oxy)-3-diazo-2-methylsuccinate (13).

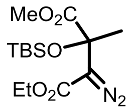

A mixture of *Z*-hydrazone **12** [2] (87 mg, 0.18 mmol) and NaOMe (5 mL, 0.1 M in MeOH) was stirred at 45 °C. After 3 h, the resulting yellow solution was extracted with Et_2O (3 × 5 mL), the combined organic layers washed with sat. aq NaCl (5 mL), dried ($MgSO_4$) and evaporated under reduced pressure. Purification of the residue twice by column chromatography (50% Et_2O in light petrol, then 30% CH_2Cl_2 in toluene), gave α-diazo ester **13** (48 mg, 76%) as a yellow liquid. Data as lit [2].

Dimethyl (4*R*,5*R*)-4-(3-((*tert*-butyldiphenylsilyl)oxy)butyl)-2,2-dimethyl-1,3-dioxolane-4,5-dicarboxylate (17) and **dimethyl (4*S*,5*R*)-4-(3-((*tert*-butyldiphenyl silyl)oxy)butyl)-2,2-dimethyl-1,3-dioxolane-4,5-dicarboxylate (18)**.

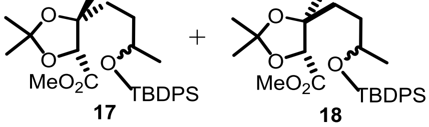

17 **18**

To a stirred solution of dimethyl (*R*,*R*)-tartrate acetonide (**7**) (1.1 g, 5 mmol), *tert*-butyl((4-iodobutan-2-yl)oxy)diphenylsilane (**16**) [3-5] (4.3 g, 10 mmol) and HMPA (3 mL) in THF (12 mL) at –78 °C, was added a precooled (–78 °C) solution of LDA (13 mL, 0.58 M in THF, 7.5 mmol) over 30 min. The temperature was maintained at –78 °C for 24 h, before phosphate buffer pH 7 (5 mL) was added and the reaction mixture extracted with EtOAc (3 × 100 mL). The combined organic layers were washed with sat. aq $CuSO_4$ (2 × 20 mL), filtered through a short pad of silica and evaporated under reduced pressure. Purification of the residue by column chromatography (10% Et_2O in light petrol) gave a colourless oil, alkylated tartrate **17** (1.3 g, 50%, 50:50 mixture of epimers in the side-chain by ^{1}H NMR analysis). The enantiopurity at the acetonide portion of **17** was determined to be >98:2 by HPLC [(Chiralcel OD, 98:2 heptane–*i*-PrOH, 0.5 mL/min), t_R(*S*,*S*-**7** derived) 7.54 min, t_R(*R*,*R*-**7** derived) 10.48 min (an ~1:1 mixture of (*R*,*R*)-**7** derived and (*S*,*S*)-**7** derived **17** gave t_Rs of 7.49 min and 10.64 min; epimers in the side-chain not resolved)].

An otherwise identical reaction to that described above, except being allowed to warm to rt overnight before buffer addition, gave the following, all as colourless oils, alkylated tartrate **17** (1.0 g, 38%, 50:50 mixture of epimers in the side-chain by ^{1}H NMR analysis), alkylated tartrate **18** (0.3 g, 12%, 60:40 mixture of epimers in the side-chain by ^{1}H NMR analysis) and recovered *tert*-butyl((4-iodobutan-2-yl)oxy)diphenylsilane (**16**) (3.0 g).

NOE studies: irradiation at the ring methine of alkylated tartrates **17** and **18** gave significant increase in NOE intensities at the side-chain methylene groups (with respect to the acetonide Me NOE intensity) for **18** compared with **17**. Also, the OMe NOE intensities of the two esters for **18** show a greater difference compared with **17**.

Data for alkylated tartrate **17**: R_f 0.60 (50% Et_2O in light petrol); ν_{max}/cm^{-1}(neat) 3071 m, 2955 s, 2858 s, 1760 s, 1748 s; δ_H(400 MHz, C_6D_6) 7.79–7.71 (8H, m, Ar–H), 7.26–7.17 (12H, m, Ar–H), 5.16 (1H, s, C*H*CO_2Me), 5.15 (1H, s, C*H*CO_2Me), 3.93–3.79 (2H, m, 2 x SiOCH), 3.33–3.30 (12H, m, 4 x CO_2Me), 2.37–2.27, 2.15–2.07, 1.98–1.80, 1.67–1.56 (14H, m, 2 x CH_2CH_2 and $C(Me)_2$), 1.44 (3H, s, C(Me*Me*)), 1.43 (3H, s, C(*Me*Me)), 1.16 and 1.15 (18H, 2 s, 2 x $C(Me)_3$), 0.98–0.92 (6H, m, 2 x CH*Me*); δ_C(100 MHz, C_6D_6) 172.6 (*C*O_2Me), 169.2 (*C*O_2Me), 169.1 (*C*O_2Me), 136.3 (Ar), 135.2 (Ar), 135.1 (Ar), 134.6 (Ar), 129.9 (Ar), 127.9 (Ar), 113.3 (*C*$(Me)_2$), 113.2 (*C*$(Me)_2$), 86.8 (quat. *C*CO_2Me), 86.6 (quat. *C*CO_2Me), 81.1 (*C*HCO_2Me), 69.8 (SiOCH), 69.7 (SiOCH), 52.1 (CO_2*Me*), 51.6 (CO_2*Me*), 51.5 (CO_2*Me*), 34.4 (CH_2), 34.2 (CH_2), 31.1 (CH_2), 30.7 (CH_2), 28.3 (Me), 28.2 (Me), 27.3 (Me), 26.8 (Me), 23.5 (Me), 23.4 (Me), 19.6 (Me); HRMS *m*/*z* ($M+NH_4^+$) found: 546.2887, $C_{29}H_{44}NO_7Si$ requires 546.2887.

Data for alkylated tartrate **18**: R_f 0.49 (50% Et_2O in light petrol); δ_H(400 MHz, C_6D_6) 7.81–7.67 (8H, m, Ar–H), 7.22–7.20 (12H, m, Ar–H), 4.38 (1H, s, C*H*CO_2Me), 4.36 (1H, s, C*H*CO_2Me), 3.91–3.82 (2H, m, 2 x SiOCH), 3.41 (3H, s, CO_2Me), 3.40 (3H, s, CO_2Me), 3.29 (3H, s, CO_2Me), 3.28 (3H, s, CO_2Me), 2.51–2.42 (1H, m, CH_2CH*H*), 2.32–2.22 (1H, m, CH_2C*H*H), 1.95–1.10 (36H, m, 2 x C*H_2*CH_2, CH_2, 2 x $C(Me)_2$ and 2 x $C(Me)_3$), 1.01–0.96 (6H, m, 2 x CH*Me*); δ_C(100 MHz, C_6D_6) 171.7 (*C*O_2Me), 171.5 (*C*O_2Me), 167.9 (*C*O_2Me), 167.8 (*C*O_2Me), 136.4 (Ar), 136.3 (Ar), 135.1 (Ar), 134.6 (Ar), 130.0 (Ar), 129.9 (Ar), 128.3 (Ar), 128.1 (Ar), 128.0 (Ar), 127.9 (Ar), 127.8 (Ar), 112.1 (*C*$(Me)_2$), 87.2 (quat. *C*CO_2Me), 82.1 (*C*HCO_2Me), 82.0 (*C*HCO_2Me), 69.5 (SiOCH), 51.9 (CO_2*Me*), 51.8 (CO_2*Me*), 34.2 (CH_2), 34.1 (CH_2), 33.0 (CH_2), 32.8 (CH_2), 27.3 (Me), 27.2 (Me), 27.1 (Me), 23.3 (Me), 19.6 (Me).

Dimethyl (5*R*)-4-hydroxy-2,2-dimethyl-5-((3-*tert*–butyldiphenylsilyl)oxy)butyl)-1,3-dioxolane-4,5-dicarboxylate (19a).

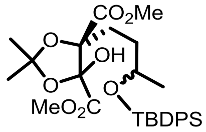

Alkylated acetonide (*R*,*R*)-**17** (100 mg, 0.188 mmol) in THF (2 mL) was added dropwise over 15 min to a stirring LDA solution [0.5 mL, 0.57 M, 0.286 mmol, prepared from iPr_2NH (40 μL), THF (0.5 mL) and *n*-BuLi (0.133 mL of 1.5 M in hexane)] at –78 °C. After 15 min at –78 °C, freshly prepared MoOPH [6] (130 mg, 0.3 mmol) was added in one portion and the reaction mixture warmed to –50 °C. After 3 h at –50 °C, the reaction mixture was quenched with sat. aq Na_2SO_3 (1 mL) and warmed to rt. Et_2O (20 mL) and H_2O (5 mL) were added and the layers were separated and the aq layer extracted with Et_2O (2 × 10 mL). The combined organic layers were washed with sat. aq $CuSO_4$ (10 mL), dried ($MgSO_4$) and evaporated under reduced pressure. Purification of the residue by column chromatography (10–50% Et_2O in light petrol) gave hydroxy acetonide **19a** (95 mg, 92%, a mixture of 4 diastereomers) as a colourless viscous oil. R_f 0.35 and 0.27 (50% Et_2O in light petrol); ν_{max}/cm^{-1}($CHCl_3$) 3493 m, 3018 m, 2956 s, 2953 s, 2859 m, 1753 s; δ_H(400 MHz, $CDCl_3$) 7.70–7.64 (16H, m, Ar–H), 7.46–7.34 (24H, m, Ar–H), 4.65–4.45 (3H, bs, OH), 3.93–3.67 (28H, m, 4 x SiOCH and 8 x CO_2Me), 2.20–0.81 (52H, complex m, 4 x CH_2CH_2, 4 x $C(Me)_2$ and 4 x CH*Me*), 1.05 (36H, s, 4 x $C(Me)_3$); δ_C(100 MHz, $CDCl_3$): 171.7 (*C*O_2Me), 171.6 (*C*O_2Me), 170.3 (*C*O_2Me), 170.2 (*C*O_2Me), 170.1 (*C*O_2Me), 169.3 (*C*O_2Me), 169.2 (*C*O_2Me), 136.0 (Ar), 135.9 (Ar), 134.8 (Ar), 134.6 (Ar), 134.3 (Ar), 134.2 (Ar), 129.7 (Ar), 129.6 (Ar), 129.5 (Ar), 127.7 (Ar), 127.6 (Ar), 127.5 (Ar), 114.2 (*C*$(Me)_2$), 113.5 (*C*$(Me)_2$), 102.1 (quat. O*C*CO_2Me), 101.9 (quat.

O*C*CO_2Me), 101.8 (quat. O*C*CO_2Me), 93.0 (quat. *C*CO_2Me), 92.7 (quat. *C*CO_2Me), 91.4 (quat. *C*CO_2Me), 91.2 (quat. *C*CO_2Me), 69.2 (SiOCH), 69.1 (SiOCH), 54.0 (CO_2*Me*), 53.9 (CO_2*Me*), 52.6 (CO_2*Me*), 52.5 (CO_2*Me*), 33.9, 33.7, 31.8, 31.4, 29.3, 29.2, 28.6, 28.4, 28.3, 27.5, (CH_2 and C(*Me*)$_2$), 27.1 [C(*Me*)$_3$], 23.4, 23.3, 23.2 (Me), 19.4 (Si*C*(Me_3)); HRMS *m/z* ($M+NH_4^+$) found: 562.2836, $C_{29}H_{44}NO_8Si$ requires 562.2833.

Dimethyl (5*R*)-4-hydroxy-2,2-dimethyl-5-propyl-1,3-dioxolane-4,5-dicarboxylate (19b).

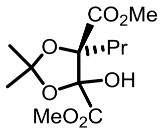

Following the procedure for hydroxy acetonide **19a**, but using propylated tartrate **33a** [7] (219 mg, 0.841 mmol) gave after column chromatography (50% Et_2O in light petrol) hydroxylated tartrate **19b** (2.54 g, 96%, 3:1 dr) as a yellow oil; R_f 0.48 and 0.35 (50% Et_2O in light petrol); ν_{max}/cm^{-1}(neat) 3487 br, 2959 s, 2876 s, 1757 s, 1439 m, 1383 m, 1267 s, 1144 s, 1101 s, 1020 m, 904 m, 864 w, 740 s, 705 s; δ_H(400 MHz, $CDCl_3$) 4.65 (1H, s, OH), 4.58 (1H, s, OH), 3.86 (3H, s, CO_2Me), 3.75 (3H, s, CO_2Me), 3.74 (3H, s, CO_2Me), 3.66 (3H, s, CO_2Me), 1.94−1.63 (4H, m, C*H*$_2$$CH_2CH_3$), 1.53 (6H, s, C(Me)$_2$), 1.51 (6H, s, C(Me)$_2$), 1.14−1.01 (4H, m, CH_2C*H*$_2$$CH_3$), 0.90−0.84 (6H, m, CH_2C*H*$_3$); δ_C(100 MHz, $CDCl_3$) 171.7 (*C*O_2Me), 170.2 (*C*O_2Me), 170.1 (*C*O_2Me), 169.0 (*C*O_2Me), 114.0 (*C*$CH_2CH_2CH_3$), 113.3 (*C*$CH_2CH_2CH_3$), 102.1 (quat. COH), 101.7 (quat. COH), 92.7 (*C*(Me)$_2$), 91.3 (*C*(Me)$_2$), 53.7 (CO_2*Me*), 52.5 (CO_2*Me*), 52.4 (CO_2*Me*), 52.3 (CO_2*Me*), 37.8 (C*C*$H_2CH_2CH_3$), 37.5 (C*C*$H_2CH_2CH_3$), 29.1 (C(*Me*)$_2$), 28.5 (C(*Me*)$_2$), 28.2 (C(*Me*)$_2$), 27.3 (C(*Me*)$_2$), 17.6 (CCH_2*C*H_2CH_3), 17.3 (CCH_2*C*H_2CH_3), 14.2 (CCH_2CH_2*C*H_3), 14.1

($CCH_2CH_2CH_3$); HRMS m/z ($M+NH_4^+$) found: 294.1546, $C_{12}H_{24}NO_7$ requires 294.1547.

Dimethyl (2*R*,*Z*)-2-((*tert*-butyldimethylsilyl)oxy)-2-(3-((*tert*–butyldiphenyl sily)oxy)butyl)-3-(2 tosylhydrazinylidene)succinate (22).

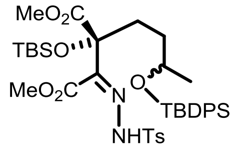

Hydroxy acetonide **19a** (370 mg, 0.68 mmol) in CH_2Cl_2 (5 mL) was added to a solution of $AlCl_3$ (182 mg, 1.37 mmol) in CH_2Cl_2 (5 mL) at –78 °C, and the mixture then warmed to –50 °C. After 7 h at –50 °C, sat. aq. $NaHCO_3$ (10 mL) was added and the mixture then warmed to rt. Et_2O (100 mL) was added and the organic layer was dried ($MgSO_4$) and evaporated under reduced pressure to give crude α-ketoester **20** (276 mg). TBSOTf (0.31 mL, 1.36 mmol) was added to a stirred mixture of 2,6-lutidine (0.23 mL, 2.05 mmol) in CH_2Cl_2 (0.5 mL) at –10 °C and after 30 min the above crude α-ketoester **20** (270 mg) in CH_2Cl_2 (0.5 mL) was added at –10 °C. The mixture was warmed to rt for 2 days before H_2O (0.2 mL) and Et_2O (10 mL) was added. The organic layer was dried ($MgSO_4$) and evaporated under reduced pressure to give crude tertiary TBS ether **21** (300 mg). A mixture of this crude tertiary TBS ether **21** and $TsNHNH_2$ (186 mg, 1.0 mmol) in THF (5 mL) was heated at 70 °C. After 30 h, the reaction mixture was evaporated under reduced pressure and the residue purified by column chromatography (10% Et_2O in light petrol) to give *Z*-hydrazone **22** [8] (266 mg, 51% overall yield from hydroxy acetonide **19a**) as a colourless viscous oil (a mixture of diastereomers). R_f 0.36 (50% Et_2O in light petrol); ν_{max}/cm^{-1}($CHCl_3$) 3180 w, 3156 w, 2956 s, 2931 s, 2858 s, 1762 m, 1704 m, 1598 w; δ_H(400 MHz, $CDCl_3$) 11.71–11.62 (2H, m, 2 x NH), 7.86–7.76 (4H, m, 4 x Ar–H),

7.71–7.65 (8H, m, 2 x Ar–H), 7.47–7.29 (12H, m, 2 x Ar–H), 7.25–7.19 (4H, m, 2 x Ar–H), 3.84–3.77 (2H, m, 2 x SiOCH), 3.75–3.59 (12H, m, 4 x CO_2Me), 2.44–2.36 (6H, m, 2 x Ar–*Me*), 2.10–0.70 (32H, m, 2 x CH_2CH_2, 2 x $C(Me)_3$ and 2 x CH*Me*), 1.06 (18H, s, 2 x $C(Me)_3$), –0.07 to –0.27 (12H, m, 2 x $Si(Me)_2$); δ_C(100 MHz, $CDCl_3$) 172.3 (*C*O_2Me), 161.7 (*C*O_2Me), 144.5, 136.9, 135.8, 135.3, 135.2, 134.8, 134.1, 129.7, 129.5, 129.4, 128.1, 127.9, 127.6, 127.4 (Ar, ArMe), 80.5 (quat. *C*CO_2Me), 80.4 (quat. *C*CO_2Me), 69.5 (SiOCH), 69.4 (SiOCH), 52.3 (CO_2*Me*), 52.1 (CO_2*Me*), 36.9, 33.3, 33.1, 32.8 (CH_2), 27.0, 25.6, 25.5, 23.2, 23.1, 22.9, 21.6, 19.3, 18.4, 18.3, 13.9 (Me, $C(Me)_3$), –3.2, –3.3, –3.4 (SiMe); HRMS *m*/*z* ($M+NH_4^+$) found: 786.3634, $C_{39}H_{60}N_3O_8SSi$ requires 786.3640.

Dimethyl (2*R*)-2-((*tert*-butyldimethylsilyl)oxy)-2-(3-((*tert*–butyldiphenyl silyl)oxy)butyl)-3-diazosuccinate (23).

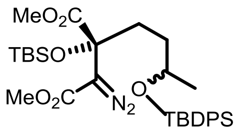

A 2:1 mixture of CH_2Cl_2/Et_3N (3 mL) was added to hydrazone **22** (200 mg, 0.26 mmol) at rt. After 4 h, the reaction mixture was evaporated under reduced pressure and the residue purified through a short column of silica gel (10% Et_2O in light petrol) to give α-diazo ester **23** (142 mg, 88%) as a yellow oil. R_f 0.76 (50% Et_2O in light petrol); ν_{max}/cm^{-1}($CHCl_3$) 2955 s, 2931 s, 2858 s, 2097 s, 1747 m, 1708 s; δ_H(400 MHz, $CDCl_3$) 7.65 (8H, d, *J* 7.5, 2 x Ar–H), 7.45–7.34 (12H, m, 2 x Ar–H), 3.86–3.80 (2H, m, 2 x SiOCH), 3.74–3.72 (12H, m, 4 x CO_2Me), 2.14–0.68 (32H, m, 2 x CH_2CH_2, 2 x $C(Me)_3$ and 2 x CH*Me*), 1.04 (18H, s, 2 x $C(Me)_3$), 0.09–0.01 (12H, m, 2 x $SiMe_2$); δ_C(100 MHz, $CDCl_3$) 171.5 (*C*O_2Me), 135.8 (Ar), 134.7 (Ar), 129.5 (Ar), 129.4 (Ar), 127.5 (Ar), 127.4 (Ar), 76.1 (quat. *C*CO_2Me), 76.0 (quat. *C*CO_2Me), 69.2

(SiOCH), 69.1 (SiOCH), 52.6 (CO_2*Me*), 51.9 (CO_2*Me*), 34.7 (CH_2), 34.3 (CH_2), 33.1 (CH_2), 27.0 (C(*Me*)$_3$), 25.7 (C(*Me*)$_3$), 23.4, 19.2, 18.4 (Me and Si*C*Me_3), –3.6 (SiMe), –4.1 (SiMe); HRMS *m/z* (M+Na^+) found: 635.2958, $C_{32}H_{48}N_2NaO_6Si_2$ requires 635.2949.

Dimethyl (4*R*,5*R*)-4-(3-((triethylsilyl)oxy)butyl)-2,2-dimethyl-1,3-dioxolane-4,5-dicarboxylate (24).

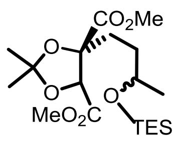

To a stirred solution of dimethyl (*R*,*R*)-tartrate acetonide (**7**) (2.85 mL, 16 mmol), triethyl((4-iodobutan-2-yl)oxy)silane [9] (5.02 g, 16 mmol) and HMPA (10 mL) in THF (50 mL) at –78 °C was added over 2 h a precooled (–78 °C) solution of LDA [prepared from iPr_2NH (2.8 mL, 20 mmol) and *n*-BuLi (12.7 mL, 1.57 M in hexane, 20 mmol) in THF (50 mL)]. After 4.5 h at –78 °C, sat. aq NH_4Cl (10 mL) was added to the mixture and the solution was extracted with Et_2O (200 mL). The organic layer was dried ($MgSO_4$), filtered through a short pad of silica, evaporated in vacuo and the residue (>90:10 dr in the acetonide portion) purified by column chromatography (10% Et_2O in petrol) to give alkylated acetonide **24** (2.1 g, 32%) as a colourless oil. R_f 0.55 (50% Et_2O in light petrol); ν_{max}/cm^{-1}($CHCl_3$) 2956 s, 1754 s, 1458 m, 1438 m; δ_H(200 MHz, $CDCl_3$) 4.88 (1H, s, C*H*CO_2Me), 4.86 (1H, s, C*H*CO_2Me), 3.75 (6H, s, 2 x CO_2Me), 3.73 (6H, s, 2 x CO_2Me), 3.71–3.59 (2H, m, 2 x SiOC*H*Me), 2.00–1.09 (20H, m, 2 x C(Me)$_2$ and 2 x CH_2CH_2), 1.06 (6H, d, *J* 6 , 2 x CH*Me*), 0.89 (18 H, t, *J* 8, 6 x $SiCH_2$*Me*), 0.57–0.44 (12H, m, 6 x $SiCH_2$); δ_C(50 MHz, $CDCl_3$) 172.4 (*C*O_2Me), 168.9 (*C*O_2Me), 168.7 (*C*O_2Me), 112.6 (*C*(Me)$_2$), 86.0 (quat. *C*CO_2Me), 85.7 (quat. *C*CO_2Me), 80.1 (*C*HCO_2Me), 68.2 (SiOCH), 68.1 (SiOCH), 52.8 (CO_2*Me*), 52.2

(CO_2Me), 33.8 (CH_2), 33.6 (CH_2), 30.9 (CH_2), 30.5 (CH_2), 27.7 (Me), 27.6 (Me), 26.1 (Me), 26.0 (Me), 23.9 (Me), 23.8 (Me), 6.9 ($SiCH_2Me$), 5.0 ($SiCH_2$); HRMS *m*/*z* ($M+H^+$) found: 405.2307, $C_{19}H_{37}O_7Si$ requires 405.2308.

Dimethyl (5*R*)-4-hydroxy-2,2-dimethyl-5-((3-(triethylsilyl)oxy)butyl)-1,3-dioxolane-4,5-dicarboxylate (25).

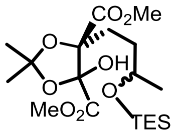

Following the procedure for hydroxy acetonide **19a**, but using alkylated acetonide **24** (1.27 g, 3.14 mmol) gave after column chromatography (10–50% Et_2O in light petrol) hydroxy acetonide **25** (1.07 g, 81%, a mixture of 4 diastereomers), as a colourless viscous oil. R_f 0.33 and 0.24 (50% Et_2O in light petrol); ν_{max}/cm^{-1} ($CDCl_3$) 3684 w, 3514 w, 3020 s, 1743 m, 1522 w; δ_H(400 MHz, $CDCl_3$) 4.59 (1H, s, OH), 4.57 (1H, s, OH), 4.48 (1H, s, OH), 3.92–3.68 (28H, m, 8 x CO_2Me and 4 x SiOCH), 2.17–1.05 (52H, complex m, 4 x CMe_2, 4 x CH_2CH_2 and 4 x CH*Me*), 0.94 (36H, td, *J* 7.5, 1.5, 4 x $SiCH_2Me$), 0.62–0.49 (24H, m, 4 x $SiCH_2$); δ_C(100 MHz, $CDCl_3$) 171.8 (CO_2Me), 171.6 (CO_2Me), 170.4 (CO_2Me), 170.3 (CO_2Me), 170.1 (CO_2Me), 169.3 (CO_2Me), 114.3 (CMe_2), 114.2 (CMe_2), 113.5 (CMe_2), 102.1 (quat. $OCCO_2Me$), 101.9 (quat. $OCCO_2Me$), 93.0 (quat. CCO_2Me), 92.7 (quat. CCO_2Me), 91.4 (quat. CCO_2Me), 91.2 (quat. CCO_2Me), 68.3 (SiOCH), 68.2 (SiOCH), 68.0 (SiOCH), 54.1 (CO_2Me), 54.0 (CO_2Me), 52.7 (CO_2Me), 52.5 (CO_2Me), 34.1, 33.8, 32.3, 31.9, 29.3, 29.2, 28.7, 28.6, 28.5, 28.4, 27.6, 27.5, 24.1, 24.0, 23.9, 23.7, (CH_2CH_2, CH*Me*, CMe_2), 7.0 ($SiCH_2Me$), 5.1 ($SiCH_2$), 5.0 ($SiCH_2$); HRMS *m*/*z* ($M+H^+$) found: 421.2262, $C_{19}H_{37}O_8Si$ requires 421.2257.

Dimethyl (2*R*)-3-diazo-2-(3-hydroxybutyl)-2-((triethylsilyl)oxy)succinate (27).

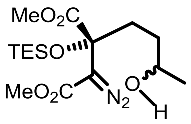

A mixture of hydroxy acetonide **25** (800 mg, 1.90 mmol) and $ZnCl_2$ (2.0 mL, 2.2 M in Et_2O, 4.4 mmol) in CH_2Cl_2 (3 mL) was stirred at rt. After 5 h, sat. aq $NaHCO_3$ (20 mL) was added, the layers were separated and the aq layer was extracted with EtOAc (3 × 100 mL). The combined organic layers were dried ($MgSO_4$) and evaporated under reduce pressure to give the crude diol (500 mg). TESCl (1.15 g, 7.6 mmol) was added to a stirred solution of this crude diol and imidazole (613 mg, 9.0 mmol) in DMF (4 mL) at –10 °C. The reaction mixture was slowly warmed to rt and kept at rt for 4 h. H_2O (10 mL) was then added and the mixture extracted with Et_2O (2 × 50 mL). The combined organic layers were dried ($MgSO_4$) and evaporated under reduced pressure to give crude bis-TES ketone **26** (1.4 g). This crude bis-TES ketone **26** and $TsNHNH_2$ (400 mg) in THF (5 mL) was stirred at reflux. After 24 h, the reaction mixture was evaporated under reduced pressure to give the corresponding hydrazone (700 mg). Et_3N (1.0 mL) was added to a solution of this hydrazone (698 mg) in THF (2 mL) at rt. After 4 h, Et_2O (5 mL) was added, the mixture was filtered through a short pad of silica and evaporated under reduced pressure. A mixture of AcOH, THF and H_2O (5 mL, 1:2:1) was added to the residue and the mixture was stirred at rt. After 4 h, the mixture was diluted with sat. aq $NaHCO_3$ (20 mL) and Et_2O (100 mL). The layers were separated and the organic layer dried ($MgSO_4$), evaporated under reduced pressure and purified by column chromatography (10–50% Et_2O in petrol) to give diazo alcohol **27** (260 mg, 37% from hydroxy acetonide **25**, as a 1:1 mixture of diastereomers), as a yellow oil. R_f 0.28 (50% Et_2O in light

petrol); ν_{max}/cm^{-1}(neat) 3434 br, 2957 s, 2878 s, 2098 s, 1747 s, 1710 s, 1438s; δ_H(400 MHz, $CDCl_3$) 3.81–3.75 (2H, m, 2 x C*H*(OH)Me), 3.74 (6H, s, 2 x CO_2Me), 3.73 (6H, s, 2 x CO_2Me), 2.12–2.08 (2H, m, 2 x CH*H*), 1.99–1.94 (2H, m, 2 x CH*H*), 1.60–1.38 (4H, m, 2 x CH_2), 1.19 (6H, d, *J* 6, 2 x CH(OH)*Me*), 0.93 (18H, t, *J* 8, 6 x $SiCH_2$*Me*), 0.64–0.55 (12H, m, 6 x $SiCH_2$); δ_C(100 MHz, $CDCl_3$) 171.8 (*C*O_2Me), 171.7 (*C*O_2Me), 165.2 (*C*O_2Me), 75.9 (quat. C), 67.7 (*C*H(OH)Me), 52.7 (CO_2*Me*), 51.9 (CO_2*Me*), 34.6 (CH_2), 33.0 (CH_2), 23.6 (Me), 6.8 ($SiCH_2$*Me*), 5.6 ($SiCH_2$); HRMS *m*/*z* ($M+NH_4^+$) found: 392.2218, $C_{16}H_{34}N_3O_6Si$ requires 392.2217.

Dimethyl (*R*)-3-diazo-2-(3-oxobutyl)-2-((triethylsilyl)oxy)succinate (28).

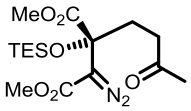

Dess-Martin periodinane (61.1 mg, 0.14 mmol) was added to a stirred solution of diazo alcohol **27** (20 mg, 0.05 mmol) in CH_2Cl_2 (1 mL) at rt. After 2 h, the reaction mixture was diluted with Et_2O (30 mL), filtered through a short pad of silica gel and evaporated under reduced pressure. Purification of the residue by column chromatography (20–40% Et_2O in light petrol) gave diazoketone **28** (19 mg, 96%) as a yellow oil. R_f 0.42 (50% Et_2O in light petrol). $[\alpha]_D^{26}$ +6.3 (*c* 1.0, $CHCl_3$); ν_{max}/cm^{-1}($CHCl_3$) 3019 s, 2955 m, 2877 w, 2102 s, 1742 m, 1703 s, 1438 s; δ_H(400 MHz, $CDCl_3$) 3.76 (3H, s, CO_2Me), 3.75 (3H, s, CO_2Me), 2.65–2.57 (1H, m, C*H*HCH_2CO), 2.49–2.42 (1H, m, C*H*HCH_2CO), 2.30–2.20 (2H, m, CH_2CO), 2.16 (3H, s, CH_2CO*Me*), 0.93 (9H, t, *J* 8, $SiCH_2$*Me*), 0.66–0.56 (6H, m, $SiCH_2$); δ_C(100 MHz, $CDCl_3$) 207.2 (CO), 171.3 (*C*O_2Me), 165.0 (NC*C*O_2Me), 75.3 ($C=N_2$), 52.8 (CO_2*Me*), 51.9 (CO_2*Me*), 37.9 (CH_2), 32.2 (CH_2), 30.0 (CH_2CO*Me*), 6.8 ($SiCH_2$*Me*), 5.6 ($SiCH_2$); HRMS *m*/*z* ($M+NH_4^+$) found: 390.2060, $C_{16}H_{32}N_3O_6Si$ requires 390.2053.

Trimethyl (1*S*,2*R*,5*S*,7*S*)-5-methyl-2-((triethylsilyl)oxy)-6,8-dioxabicyclo[3.2.1] octane-1,2,7-tricarboxylate (29).

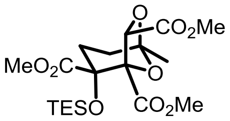

$Rh_2(OAc)_4$ (~1 mg, cat.) was added to a stirred solution of diazoketone **28** (40 mg, 0.106 mmol) in toluene (0.3 mL) and freshly distilled methyl glyoxylate [10] (43.9 mg, 0.5 mmol) at 110 °C. After 4 h, the reaction mixture was cooled, diluted with Et_2O (5 mL), filtered through celite and evaporated under reduced pressure. Purification of the residue by column chromatography (20% Et_2O in light petrol) gave cycloadduct **29** (29 mg, 63%) as a colourless oil. R_f 0.36 (50% Et_2O in light petrol); $[\alpha]_D^{26}$ +46.0 (*c* 1.0, $CHCl_3$); ν_{max}/cm^{-1}($CHCl_3$) 2955 m, 1753 s, 1298 m; δ_H(400 MHz, $CDCl_3$) 5.54 (1H, s, C*H*CO_2Me), 3.80 (3H, s, CO_2Me), 3.70 (3H, s, CO_2Me), 3.67 (3H, s, CO_2Me), 2.44–2.34 (1H, m, TESO(CO_2Me)CH*H*), 1.95–1.83 (1H, m, TESO(CO_2Me)CH_2CH*H*), 1.75–1.64 (2H, m, TESO(CO_2Me)C*H*H, TESO(CO_2Me)CH_2C*H*H and 3H, s, Me), 0.93 (9H, t, *J* 9, SiCH_2*Me*), 0.73–0.55 (6H, m, SiCH_2); δ_c(100 MHz, $CDCl_3$) 173.5 (*C*O_2Me), 169.7 (*C*O_2Me), 166.8 (*C*O_2Me), 110.4 (O–C–O), 90.5 (quat.C), 79.7 (quat. C), 77.6 (*C*HCO_2Me), 52.8 (CO_2*Me*), 52.6 (CO_2*Me*), 52.6 (CO_2*Me*), 30.9 (CH_2), 30.1 (CH_2), 24.0 (Me), 7.1 (SiCH_2*Me*), 6.7 (SiCH_2); HRMS *m*/*z* ($M+H^+$) found: 433.1903, $C_{19}H_{33}O_9Si$ requires 433.1894.

Trimethyl (1*S*,2*R*,5*S*,7*S*)-2-hydroxy-5-methyl-6,8-dioxabicyclo [3.2.1]octane-1,2,7-tricarboxylate (30) and **trimethyl (1*S*,3*S*,4*S*,5*R*)-4-hydroxy-1-methyl-2,8-dioxabicyclo[3.2.1]octane-3,4,5-tricarboxylate (31).**

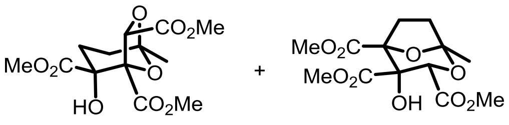

A solution of cycloadduct **29** (30 mg, 0.07 mmol) in a mixture of CH_2Cl_2, TFA and H_2O (1 mL, 20:10:1) was stirred at rt. After 48 h, the reaction mixture was evaporated under reduced pressure. Analysis of the residue by ^{1}H NMR indicated a 40:60 ratio of alcohols **30**:**31**, by data comparison with lit [11].

Dimethyl (4*R*,5*R*)-2,2,4-trimethyl-1,3-dioxolane-4,5-dicarboxylate (32).

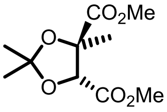

A solution of dimethyl (*R*,*R*)-tartrate acetonide (**7**) (1.0 mL, 5.45 mmol), MeI (1.0 mL, 16.4 mmol) and anhydrous LiCl (1.39 g, 32.7 mmol) in THF (6 mL) was cooled to –78 °C. To this mixture was added dropwise a pre-cooled (–78 ºC) solution of LiHMDS (prepared by adding *n*-BuLi (2.6 mL, 2.5 M in hexanes, 6.54 mmol) dropwise to freshly distilled HMDS (1.5 mL, 7.09 mmol) in THF (4 mL) at 0 °C). The reaction mixture was slowly warmed to rt and stirred for 16 h. The mixture was then poured into EtOAc (20 mL) and washed with H_2O (3 × 20 mL), brine (30 mL), dried (Na_2SO_4), filtered, evaporated under reduced pressure and purified by column chromatography (20% EtOAc in petrol) to give methylated tartrate **32** (0.48 g, 39%) as colourless oil. R_f 0.47 (20 % EtOAc in petrol); $[\alpha]_D^{25}$ −66.6 (*c* 1.0, $CHCl_3$); ν_{max}/cm$^-$

[1](neat) 2993 w, 2956 w, 1739 s, 1116 s; δ_H(400 MHz, $CDCl_3$) 5.11 (1H, s, CH), 3.82 (3H, s, CO_2Me), 3.79 (3H, s, CO_2Me), 1.60 (3H, s, CO_2MeCCH_3), 1.41 (3H, s, one of $C(CH_3)_2$, 1.40 (3H, s, one of $C(CH_3)_2$); δ_c(100 MHz, $CDCl_3$) 172.7 (CO_2Me), 169.2 (CO_2Me), 112.5 ($C(CH_3)_2$), 83.0 (CO_2MeCCH_3), 79.7 (CO_2MeCH), 53.2 (CO_2Me), 52.4 (CO_2Me), 27.9 (CO_2MeCCH_3), 26.1 (CCH_3), 20.8 (CCH_3); HRMS *m/z* ($M+Na^+$) found: 255.0839, $C_{10}H_{16}NaO_6$ requires 255.0839.

Dimethyl (4*R*,5*R*)-2,2-dimethyl-4-propyl-1,3-dioxolane-4,5-dicarboxylate (33a) and **dimethyl (4*RS*,5*RS*)-2,2-dimethyl-4,5-dipropyl-1,3-dioxolane-4,5-dicarboxylate (*rac*-34a).**

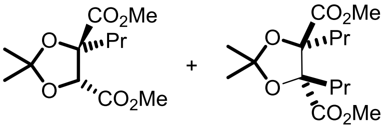

A stirred solution of dimethyl (*R*,*R*)-tartrate acetonide (**7**) (4.76 g, 21.8 mmol), 1-iodopropane (2.00 mL, 20.5 mmol) and freshly distilled HMPA (20 mL) in THF (100 mL) was cooled to –78 °C. To this mixture was added dropwise over 1 h a pre-cooled (–78 °C) solution of LDA (freshly prepared by adding *n*-BuLi (17 mL, 1.6 M in hexanes, 27 mmol) dropwise to a solution of freshly distilled iPr_2NH (3.2 g, 30 mmol) in THF (100 mL) at 0 °C. The reaction mixture was further stirred for 48 h at –78 °C, then quenched at that temperature with sat. aq NH_4Cl (50 mL) and extracted with EtOAc (3 × 100 mL). The combined organic layers were washed with sat. aq $CuSO_4$ (100 mL), dried ($MgSO_4$), evaporated under reduced pressure and purified by column chromatography to give propylated tartrate **33a** (3.51 g, 66%) as a colourless oil. The er was determined to be 97:3 by HPLC [(Chiralcel OD-H, 99:1 hexane–iPrOH, 1.0 mL/min, λ = 220 nm, 25 μL injection) t_R(minor) 8.43 min, t_R(major) 10.92

min]. R_f 0.4 (10% Et_2O in petrol); $[\alpha]_D^{25}$ –54.6 (*c* 1.0, $CHCl_3$); ν_{max}/cm^{-1}(neat) 2963 s, 2859 s, 1736 s, 1655 m, 1561 m, 1459 s, 1375 m, 1214 s, 1104 s, 1023 s, 849 m; δ_H(500 MHz, $CDCl_3$) δ 4.83 (1H, s, C*H*CO_2Me), 3.71 (3H, s, CO_2Me), 3.70 (3H, s, CO_2Me), 1.66 (1H, dt, *J* 12, 5, CH*H*CH_2CH_3), 1.49 (3H, s, on of $C(CH_3)_2$), 1.48–1.43 (1H, m, CH*H*CH_2CH_3), 1.40–1.33 (1H, m, CH_2CH*H*CH_3), 1.31 (3H, s, one of $C(CH_3)_2$), 1.20–1.08 (1H, m, CH_2CH*H*CH_3), 0.79 (3H, t, *J* 7.0, CH_2*CH_3*); δ_c(125 MHz, $CDCl_3$) 172.5 (*C*O_2Me), 168.9 (*C*O_2Me), 112.4 (*C*$(CH_3)_2$), 85.9 (CO_2Me*C*$CH_2CH_2CH_3$), 79.9 (*C*HCO_2Me), 52.6 (CO_2*Me*), 52.2 (CO_2*Me*), 36.3 (*C*$H_2CH_2CH_3$), 27.6 and 25.9 (C(*C*$H_3)_2$), 17.2 (CH_2*C*H_2CH_3), 14.1 (CH_2CH_2*C*H_3); HRMS *m/z* ($M+H^+$), found 261.1335, $C_{12}H_{21}O_6$ requires 261.1333.

Also isolated was *trans*-dipropylated tartrate ***rac*-34a** (209 mg, 7%) as a yellow liquid; R_f 0.18 (10% Et_2O in petrol); ν_{max}/cm^{-1}(KBr) 2932 s, 2862 s, 1763 s, 1658 s, 1443 s, 1381 s, 1321 s, 1221 s, 1143 s, 1059 s, 933 s, 850 s; δ_H(400 MHz, $CDCl_3$) 3.78 (6H, s, 2 × CO_2Me), 1.89 (2H, ddd, *J* 13, 12 and 4.5, 2 x CH*H*CH_2CH_3), 1.63–1.45 (10H, m, 2 x C*H*HCH_2CH_3, 2 x CH_2C*H*HCH_3 and $C(CH_3)_2$), 1.12–1.02 (2H, m, 2 × CH*H*), 0.86 (6H, t, *J* 7, 2 × CH_2CH_2C*H_3*); δ_C(100 MHz, $CDCl_3$) 171.0 (*C*O_2Me), 112.0 (*C*$(CH_3)_2$), 90.1 (*C*$CH_2CH_2CH_3$), 52.5 (CO_2*Me*), 37.6 (C*C*$H_2CH_2CH_3$), 29.2 (C(*C*$H_3)_2$), 17.5 (CCH_2*C*H_2CH_3), 14.2 (CH_2*C*H_3); HRMS *m/z* ($M+H^+$) found: 303.1807, $C_{15}H_{27}O_6$ requires 303.1802.

Dimethyl (4*R*,5*R*)-2,2-dimethyl-4,5-dipropyl-1,3-dioxolane-4,5-dicarboxylate ((*R*,*R*)-34a).

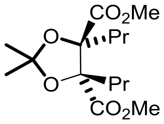

A stirred solution of mono-propylated tartrate **33a** (228 mg, 0.87 mmol), 1-iodopropane (100 µL, 1.04 mmol) and HMPA (0.7 mL) in THF (3.5 mL) was cooled to –78 °C. To this reaction mixture was added dropwise a pre-cooled (–78 °C) solution of LDA [freshly prepared by adding dropwise *n*-BuLi (0.45 µL, 2.5 M in hexanes, 1.13 mmol) to a solution of freshly distilled iPr_2NH (170 µL, 1.21 mmol) in THF (3.5 mL) at 0 °C]. The reaction mixture was warmed to –50 °C and stirred for 62 h, then quenched at that temperature with sat. aq NH_4Cl (30 mL) and extracted with EtOAc (3 × 40 mL). The combined organic layers were washed with sat. aq $CuSO_4$ (20 mL), dried ($MgSO_4$), evaporated under reduced pressure and purified by column chromatography to give *trans*-dipropylated tartrate **(*R*,*R*)-34a** (90 mg, 34%) as a colourless oil. The er was determined to be 98:2 by HPLC [(Chiralpak IC-3, 99:1 heptane–iPrOH, 1.0 mL/min, λ = 230 nm, 10 μL injection) t_R(minor) 4.19 min, t_R(major) 4.61 min]; $[\alpha]_D^{25}$ −1.1 (*c* 1.4, $CHCl_3$). Other data as above for ***rac*-34a**.

Dimethyl (4*R*,5*R*)-4-butyl-2,2-dimethyl-1,3-dioxolane-4,5-dicarboxylate (33b) and **dimethyl (4*RS*,5*RS*)-4,5-dibutyl-2,2-dimethyl-1,3-dioxolane-4,5-dicarboxylate (34b).**

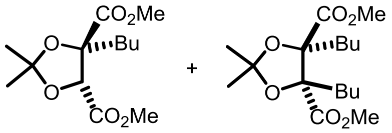

33b **34b**

A solution of LDA (prepared from iPr_2NH (420 μL, 3.0 mmol) and *n*-BuLi (1.9 mL, 1.6 M in hexanes, 3.0 mmol) in THF (7.0 mL) at −78 ºC) was added dropwise over 3 h to a solution of dimethyl (*R*,*R*)-tartrate acetonide (**7**) (500 μL, 2.7 mmol) and BuI (470 mL, 4.1 mmol) in a mixture THF/HMPA (9.5 mL, 3/1 (v/v)) at −78 ºC. The reaction mixture was stirred at −78 °C. After 24 h, sat. aq NH_4Cl was added. The mixture was warmed to rt and diluted with Et_2O and H_2O. The aq layer was extracted with Et_2O and the organic layer dried ($MgSO_4$) and concentrated under reduced pressure. Purification of the residue by column chromatography (10–20% Et_2O in petrol) gave butylated tartrate **33b** (311 mg, 42%) as a yellow oil; R_f 0.29 (20% Et_2O in petrol); $[\alpha]_D^{26}$ –43.8 (*c* 2.5, $CHCl_3$); ν_{max}/cm^{-1}(neat) 2958 w, 1753 s, 1439 w, 1384 w, 1214 m; δ_H(400 MHz, $CDCl_3$) 4.93 (1H, s, C*H*CO_2Me), 3.80 (3H, s, CO_2Me), 3.79 (3H, s, CO_2Me), 1.77 (1H, ddd, *J* 13, 12 and 4.5, (CO_2Me)CCH*H*), 1.66–1.56 (4H, m, (CO_2Me)CC*H*H and one of $(CH_3)_2$), 1.41 (3H, s, one of $(CH_3)_2$), 1.34–1.14 (4H, m. C*H*$_2$C*H*$_2$$CH_3$), 0.86 (3H, t, *J* 7.5, CH_2C*H*$_3$); δ_C(100 MHz, $CDCl_3$) 172.8 (*C*O_2Me), 169.1 (*C*O_2Me), 112.6 (*C*$(CH_3)_2$), 86.1 ((CO_2Me)*C*CH_2), 80.1 (*C*HCO_2Me), 52.9 (CO_2*Me*), 52.4 (CO_2*Me*), 34.0 ((CO_2Me)C*C*H_2), 27.8 and 26.1 (C(*C*H_3)$_2$), 26.1 (CH_2), 22.9 (CH_2), 14.0 (CH_2*C*H_3); HRMS *m*/*z* ($M+H^+$) found: 275.1514, $C_{13}H_{23}O_6$ requires 275.1495.

Also isolated was *trans*-dibutylated tartrate **34b** (90 mg, 10%) as a yellow oil; R_f 0.51 (20% Et_2O in petrol); ν_{max}/cm^{-1}(neat) 2960 w, 1748 m, 1460 w, 1383 w, 1217 w; δ_H(400 MHz, $CDCl_3$) 3.79 (6H, s, 2 x CO_2Me), 1.92 (2H, ddd, *J* 13, 12 and 4.5, 2 x (CO_2Me)CC*H*H), 1.66–1.61 (2H, ddd, *J* 13, 12 and 4.5, 2 x (CO_2Me)CCH*H*), 1.55 (6H, s, $(CH_3)_2$), 1.52–1.42 (2H, m, 2 x CH*H*), 1.29–123 (4H, m, 2 x CH_2), 1.10–0.99 (2H, m, 2 x CH*H*), 0.85 (6H, t, *J* 7.5, CH_2CH_3); δ_C(100 MHz, $CDCl_3$) 171.0 (2 x *C*O_2Me), 112.0 (*C*$(CH_3)_2$), 90.1 (2 x (CO_2Me)*C*CH_2), 52.5 (2 x CO_2*Me*), 35.2 (2 x (CO_2Me)C*C*H_2), 29.2 (2 x CH_2), 26.3 (C(*C*H_3)$_2$), 22.8 (2 x CH_2), 14.0 (2 x CH_2*C*H_3); HRMS *m*/*z* (M+Na^+) found: 353.1934, $C_{17}H_{30}O_6Na$ requires 353.1934.

Dimethyl (4*R*,5*R*)-4-hexyl-2,2-dimethyl-1,3-dioxolane-4,5-dicarboxylate (33c) and **dimethyl (4*RS*,5*RS*)-4,5-dihexyl-2,2-dimethyl-1,3-dioxolane-4,5-dicarboxylate (34c).**

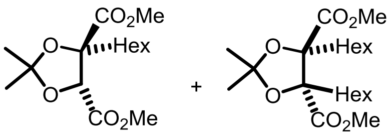

33c **34c**

A solution of LDA (prepared from iPr_2NH (2.00 mL, 14.3 mmol) and *n*-BuLi (6.20 mL, 1.6 M in hexanes, 9.92 mmol) in THF (50 mL) at −78 ºC) was added dropwise over 4 h to a mixture of dimethyl (*R*,*R*)-tartrate acetonide (**7**) (1.40 mL, 7.62 mmol) and 1-iodohexane (1.00 mL, 6.78 mmol) in THF (50 mL) and HMPA (10 mL) at −78 ºC. After 12 h, sat. aq NH_4Cl (50 mL) was added, the organic layer was separated, and the aq layer was extracted with Et_2O (3 × 100 mL). The combined organic layers were dried ($MgSO_4$) and concentrated under reduced pressure. Purification of the residue by column chromatography (10% Et_2O in petrol) gave monohexylated tartrate **33c** (967 mg, 47%) as a yellow oil; R_f 0.14 (10% Et_2O in petrol); $[\alpha]_D^{23}$ −27.5

(c 1.0, $CHCl_3$) ; ν_{max}/cm^{-1}(neat) 2956 s, 2859 s, 1761 s, 1438 m, 1374 m, 1209 s, 1105 s, 995 m, 864 m; δ_H(400 MHz, $CDCl_3$) 4.93 (1H, s, C*H*CO_2Me), 3.81 (3H, s, CO_2Me), 3.80 (3H, s, CO_2Me), 1.82–1.73 (1H, m, (CO_2Me)CC*H*H), 1.67–1.62 (1H, m, (CO_2Me)CCH*H*), 1.60 (3H, s, one of $C(CH_3)_2$), 1.41 (3H, s, one of $C(CH_3)_2$), 1.29–1.17 (8H, m, $CH_2CH_2CH_2CH_2CH_2CH_3$), 0.86 (3H, t, J 7, CH_2CH_3); δ_C(100 MHz, $CDCl_3$) 172.8 (*C*O_2Me), 169.2 (*C*O_2Me), 112.7 (*C*$(CH_3)_2$), 86.2 ((CO_2Me)*C*CH_2), 80.1 (*C*HCO_2Me), 52.9 (CO_2*Me*), 52.4 (CO_2*Me*), 34.4 ((CO_2Me)C*C*H_2), 31.7 (CH_2), 29.5 (CH_2), 27.8 and 26.2 (C(*C*$H_3)_2$), 23.9 (CH_2), 22.6 (CH_2), 14.1 (CH_2*C*H_3); HRMS *m*/*z* (M+H$^+$) found: 303.1803, $C_{15}H_{27}O_6$ requires 303.1802.

Also isolated was *trans*-dihexylated tartrate **34c** (236 mg, 9%) as a yellow oil; R_f 0.47 (10% Et_2O in petrol); ν_{max}/cm^{-1}(neat) 2932 s, 2862 s, 1756 s, 1658 s, 1459 s, 1380 s, 1321 s, 1242 s, 1143 s, 1058 s, 910 s, 859 s; δ_H(400 MHz, $CDCl_3$) 3.75 (6H, s, 2 × CO_2Me), 1.88 (1H, ddd, J 13, 12 and 4.5, (CO_2Me)CCH*H*), 1.62−1.41 (10H, m, 2 × (CO_2Me)CC*H*H, CH_2 and C(C*H*$_3)_2$), 1.29−1.13 (12H, m, 5 × CH_2 and 2 × CH*H*), 1.07−0.94 (2H, m, 2 × CH*H*), 0.81 (6H, t, J 7, 2 × CH_2CH_3); δ_C(100 MHz, $CDCl_3$) 171.0 (2 x *C*O_2Me), 112.0(*C*$(CH_3)_2$), 90.1 (2 x (CO_2Me)*C*CH_2), 52.4 (2 x CO_2*Me*), 35.5 ((CO_2Me)C*C*H_2), 31.6 (2 x CH_2), 29.3 (C(*C*$H_3)_2$), 29.1 (2 x CH_2), 24.0 (2 x CH_2), 22.5 (2 x CH_2), 14.0 (2 x CH_2*C*H_3); HRMS *m*/*z* (M+H$^+$) found: 387.2741, $C_{21}H_{39}O_6$ requires 387.2741.

Dimethyl (4*R*,5*R*)-4-benzyl-2,2-dimethyl-1,3-dioxolane-4,5-dicarboxylate (33d) and **dimethyl (4*RS*,5*RS*)-4,5-dibenzyl-2,2-dimethyl-1,3-dioxolane-4,5-dicarboxylate (34d).**

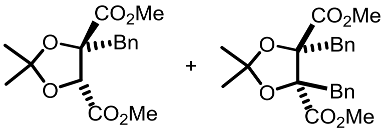

33d **34d**

A solution of LDA [prepared from iPr_2NH (0.700 mL, 4.99 mmol) and *n*-BuLi (2.80 mL, 1.6 M in hexanes, 4.48 mmol) in THF (15 mL) at −78 ºC] was added dropwise over 3 h to a mixture of dimethyl (*R*,*R*)-tartrate acetonide (**7**) (1.10 mL, 4.11 mmol) and BnBr (0.50 mL, 4.20 mmol) in THF (15 mL) and HMPA (3 mL) at −78 ºC. After 12 h, sat. aq NH_4Cl (20 mL) was added, the organic layer was separated and the aq layer was extracted with Et_2O (3 × 20 mL). The combined organic layers were dried ($MgSO_4$) and concentrated under reduced pressure. Purification of the residue by column chromatography (10% Et_2O in petrol) gave monobenzylated tartrate **33d** [12,13] (781 mg, 60%) as a yellow oil; R_f 0.12 (10% Et_2O in petrol); $[\alpha]_D^{23}$ −38.3 (*c*=1.0, $CHCl_3$); ν_{max}/cm^{-1}(neat) 2993 s, 2955 s, 1757 s, 1605 s, 1497 s, 1439 s, 1375 s, 1230 s, 922 m, 858 m; δ_H(500 MHz, $CDCl_3$) 7.29−7.16 (5H, m, ArH), 4.95 (1H, s, C*H*CO_2Me), 3.86 (3H, s, CO_2Me), 3.66 (3H, s, CO_2Me), 3.12 (1H, d, *J* 14.5, CH*H*Ph), 2.91 (1H, d, *J* 14.5, C*H*HPh), 1.71 (3H, s, one CH_3 of C(CH_3)$_2$), 1.42 (3H, s, one CH_3 of C(CH_3)$_2$); δ_C(125 MHz, $CDCl_3$) 172.1 (*C*O_2Me), 168.9 (*C*O_2Me), 134.9 (quat. ArC), 130.4 (ArC), 128.4 (ArC), 127.2 (ArC), 113.3 (*C*(CH_3)$_2$), 86.3 (*C*CH_2Ph), 80.4 (*C*HCO_2Me), 52.7 (CO_2*Me*), 52.7 (CO_2*Me*), 40.6 (*C*H_2Ph), 27.9 (C(*C*H_3)$_2$), 25.9 (C(*C*H_3)$_2$); HRMS *m*/*z* (M+H^+) found: 309.1334, $C_{16}H_{21}O_6$ requires 309.1332.

Also isolated was *trans*-dibenzylated tartrate **34d** (211 mg, 13%) as a white solid; R_f 0.14 (10% Et_2O in petrol); mp 40−43 °C; ν_{max}/cm^{-1}(KBr) 3055 m, 2987 m, 2306 w, 1753 s, 1643 s, 1422 m, 1266 s, 1092 w, 896 m; δ_H(400 MHz, $CDCl_3$) 7.29−7.18 (10H, m, ArH), 3.73 (6H, s, 2 × CO_2Me), 3.41 (2H, d, *J* 13.5, 2 × CH*H*Ph), 2.90 (2H, d, *J* 13.5, 2 × C*H*HPh), 1.75 (6H, s, 2 × $C(CH_3)_2$); δ_C(100 MHz, $CDCl_3$) 170.5 (CO_2Me), 135.1 (quat. ArC), 130.6 (ArC), 128.4 (ArC), 127.2 (ArC), 113.7 ($C(CH_3)_2$), 91.1 (CCH_2Ph), 52.5 (CO_2Me), 41.9 (CH_2Ph), 29.6 ($C(CH_3)_2$); HRMS *m*/*z* ($M+NH_4^+$) found: 416.2070, $C_{23}H_{30}NO_6$ requires 416.2068.

Dimethyl (4*R*,5*R*)-4-allyl-2,2-dimethyl-1,3-dioxolane-4,5-dicarboxylate (33e) and **dimethyl (4*RS*,5*RS*)-4,5-diallyl-2,2-dimethyl-1,3-dioxolane-4,5-dicarboxylate (34e).**

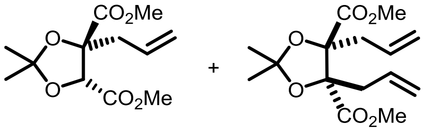

33e **34e**

A solution of LDA (prepared from iPr_2NH (1.0 mL, 7.14 mmol) and *n*-BuLi (4.00 mL, 1.6 M in hexanes, 6.40 mmol) in THF (20 mL) at −78 ºC) was added dropwise over 3 h to a mixture of dimethyl (*R*,*R*)-tartrate acetonide (**7**) (1.50 mL, 5.60 mmol) and allyl bromide (0.50 mL, 5.78 mmol) in THF (20 mL) and HMPA (4 mL) at −78 ºC. After 12 h, sat. aq NH_4Cl (20 mL) was added, the organic layer was separated and the aq layer was extracted with Et_2O (3 × 20 mL). The combined organic layers were dried ($MgSO_4$) and concentrated under reduced pressure. Purification of the residue by column chromatography (10% Et_2O in petrol) gave monoallylated tartrate **33e** [12,14] (511 mg, 35%) as a yellow oil; R_f 0.1 (10% Et_2O in petrol); $[\alpha]_D^{23}$ −35.1 (*c* 1.0, $CHCl_3$); ν_{max}/cm^{-1}(neat) 3081 m, 2985 s, 2867 s, 1762 s, 1643 s, 1438 s, 1383 s, 1216 s, 996

s, 923 s; δ_H(400 MHz, $CDCl_3$) 5.80−5.67 (1H, m, CH_2CH=CH_2), 5.12−5.03 (2H, m, CH_2CH=CH_2), 4.97 (1H, s, $CHCO_2Me$), 3.78 (3H, s, CO_2Me), 3.77 (3H, s, CO_2Me), 2.56 (1H, dd, *J* 14 and 7, $CHHCH$=CH_2), 2.43 (1H, dd, *J* 14 and 7, $CHHCH$=CH_2), 1.60 (3H, s, one CH_3 of $C(CH_3)_2$), 1.40 (3H, s, one CH_3 of $C(CH_3)_2$); δ_C(100 MHz, $CDCl_3$) 172.0 (CO_2Me), 168.8 (CO_2Me), 131.4 (CH_2=*C*H), 119.5 (*C*H_2=CH), 112.8 (*C*$(CH_3)_2$), 85.5 (*C*CH_2CH=CH_2), 79.6 (*C*HCO_2Me), 52.9 (CO_2*Me*), 52.4 (CO_2*Me*), 39.0 (*C*H_2CH=CH_2), 27.6 ($C(CH_3)_2$), 26.0 ($C(CH_3)_2$) ; HRMS *m*/*z* ($M+NH_4^+$) found: 276.1442, $C_{12}H_{22}NO_6$ requires 276.1442.

Also isolated was *trans*-diallylated tartrate **34e** (201 mg, 14%) as a yellow oil; R_f 0.2 (10% Et_2O in petrol); ν_{max}/cm^{-1}(neat) 3081 m, 2988 s, 2953 s, 1756 s, 1643 s, 1437 s, 1372 s, 1321 s, 1221 s, 1141 s, 1057 s, 997 s, 924 s; δ_H(400 MHz, $CDCl_3$) 5.71−5.10 (2H, m, 2 × CH_2CH=CH_2), 5.07−5.00 (4H, m, 2 × CH_2CH=CH_2), 3.72 (6H, s, 2 × CO_2Me), 2.67 (2H, dd, *J* 13.5 and 8, 2 × $CHHCH$=CH_2), 2.35 (2H, dd, *J* 13.5 and 6, 2 × $CHHCH$=CH_2), 1.53 (6H, s, 2 × $C(CH_3)_2$); δ_C(100 MHz, $CDCl_3$) 170.3 (CO_2Me), 131.6 (CH_2=*C*H), 119.7 (*C*H_2=CH), 112.9 (*C*$(CH_3)_2$), 89.8 (*C*CH_2CH=CH_2), 52.5 (CO_2*Me*), 40.3 (*C*H_2CH=CH_2), 29.0 ($C(CH_3)_2$); HRMS *m*/*z* ($M+H^+$) found: 299.1488, $C_{15}H_{23}O_6$ requires 299.1489.

Dimethyl (4*R*,5*R*)-2,2-dimethyl-4-(3-methylbut-2-enyl)-1,3-dioxolane-4,5-dicarboxylate (33f) and **dimethyl (4*RS*,5*RS*)-2,2-dimethyl-4,5-bis(3-methylbut-2-enyl)-1,3-dioxolane-4,5-dicarboxylate (34f).**

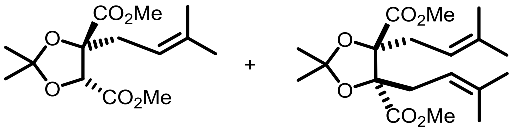

33f **34f**

A solution of LDA (prepared from iPr_2NH (2.00 mL, 14.3 mmol) and *n*-BuLi (7.00 mL, 1.6 M in hexanes, 11.2 mmol) in THF (40 mL) at −78 ºC) was added dropwise over 1 h to a mixture of dimethyl (*R*,*R*)-tartrate acetonide (**7**) (1.70 mL, 9.26 mmol) and 4-bromo-2-methyl-2-butene (1.0 mL, 8.68 mmol) in THF (50 mL) and HMPA (10 mL) at −78 ºC. After 12 h, sat. aq NH_4Cl (40 mL) was added, the organic layer was separated and the aq. layer was extracted with Et_2O (3 × 100 mL). The combined organic layers were dried ($MgSO_4$) and concentrated under reduced pressure. Purification of the residue by column chromatography (10% Et_2O in petrol) gave monoprenylated tartrate **33f** [12,15] (1.67 g, 67%) as a yellow oil; R_f 0.11 (10% Et_2O in petrol); $[\alpha]_D^{23}$ −38.8 (*c* 1.0, $CHCl_3$), lit [15] $[\alpha]_D^{20}$ −30.2 (*c* 9.5, $CHCl_3$); ν_{max}/cm^{-1}(neat) 2992 s, 2954 s, 1761 s, 1764 s, 1438 m, 1383 m, 1210 s, 1108 s, 1015 m, 854 m; δ_H(400 MHz, $CDCl_3$) 5.06 (1H, t, *J* 7, $CH_2CH{=}C(CH_3)_2$), 4.97 (1H, s, $CHCO_2Me$), 3.77 (3H, s, CO_2Me), 3.76 (3H, s, CO_2Me), 2.52 (1H, dd, *J* 14.5 and 7, $CHHCH{=}C(CH_3)_2$), 2.39 (1H, dd, *J* 14.5 and 7, $CHHCH{=}C(CH_3)_2$), 1.67 (3H, s, one CH_3 of $CH{=}C(CH_3)_2$), 1.60 (3H, s, one CH_3 of $C(CH_3)_2$), 1.56 (3H, s, one CH_3 of $CH{=}C(CH_3)_2$), 1.39 (3H, s, one CH_3 of $C(CH_3)_2$); δ_C(100 MHz, $CDCl_3$) 172.5 (CO_2Me), 169.1 (CO_2Me), 136.1 ($CH{=}C(CH_3)_2$), 116.6 ($CH{=}C(CH_3)_2$), 112.7 ($C(CH_3)_2$), 86.0 ($CCH_2CH{=}C(CH_3)_2$), 79.5 ($CHCO_2Me$), 52.8 (CO_2Me), 52.3

(CO_2*Me*), 33.4 (*C*H_2CH=(CH_3)$_2$), 27.6 (C(*C*H_3)$_2$), 26.1 (CH=C(*C*H_3)$_2$), 26.0 (C(*C*H_3)$_2$), 18.1 (CH=C(*C*H_3)$_2$); HRMS *m*/*z* ($M+H^+$) found: 287.1489, $C_{14}H_{23}O_6$ requires 287.1489.

Also isolated was *trans*-diprenylated tartrate **34f** [15] (443 mg, 14%) as a yellow crystalline solid; R_f 0.27 (10% Et_2O in petrol); mp 36−38 °C; ν_{max}/cm^{-1}(KBr) 2937 s, 2801 s, 1752 s, 1455 s, 1385 s, 1316 s, 1233 s, 1180 s, 1080 s, 945 m, 848 m; δ_H(400 MHz, $CDCl_3$) 5.02 (2H, t, *J* 7, 2 × CH_2C*H*=C(CH_3)$_2$), 3.70 (6H, s, 2 × CO_2Me), 2.71 (2H, dd, *J* 14 and 8, 2 × CH*H*CH=C(CH_3)$_2$), 2.18 (2H, dd, *J* 14 and 6, 2 × C*H*HCH=C(CH_3)$_2$), 1.60 (6H, s, C(C*H*$_3$)$_2$), 1.52 (12H, s, 2 x CH_2CH=C(C*H*$_3$)$_2$); δ_C(100 MHz, $CDCl_3$) 170.9 (*C*O_2Me), 135.8 (CH=*C*(CH_3)$_2$), 117.1 (*C*H=C(CH_3)$_2$), 112.5 (*C*(CH_3)$_2$), 89.9 (*C*CH_2CH=C(CH_3)$_2$), 52.2 (CO_2*Me*), 34.8 (*C*H_2CH=C(CH_3)$_2$), 28.9 (CH=C(*C*H_3)$_2$), 25.9 (C(*C*H_3)$_2$), 18.1 (CH=C(*C*H_3)$_2$); HRMS *m*/*z* ($M+H^+$) found: 355.2115, $C_{19}H_{31}O_6$ requires 355.2115.

Dimethyl (4*R*,5*R*)-2,2-dimethyl-4-(but-3-enyl)-1,3-dioxolane-4,5-dicarboxylate (38a).

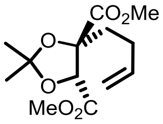

A solution of LDA (prepared from iPr_2NH (2.00 mL, 14.3 mmol) and *n*-BuLi (9.0 mL, 1.6 M in hexane, 14.4 mmol) in THF (40 mL) at −78 ºC) was added dropwise over 1 h to a mixture of dimethyl (*R*,*R*)-tartrate acetonide (**7**) (2.00 mL, 10.9 mmol) and 4-bromo-1-butene (1.00 mL, 9.85 mmol) in THF (50 mL) and HMPA (10 mL) at −78 ºC. After 12 h, saturated aq NH_4Cl (40 mL) was added, the organic layer was separated and the aqueous layer was extracted with Et_2O (3 × 100 mL). The combined organic

layers were dried ($MgSO_4$) and concentrated under reduced pressure. Purification of the residue by column chromatography (10% Et_2O in petrol) gave monoalkylated tartrate **38a** (341 mg, 13%) as a yellow oil; R_f 0.06 (10% Et_2O in petrol); $[\alpha]_D^{23}$ −28.2 (*c* 1.0, $CHCl_3$); ν_{max}/cm^{-1}(neat) 3079 m, 2993 s, 2955 s, 1760 s, 1643 s, 1459 s, 1383 s, 1212 s, 995 s, 916 s; δ_H(400 MHz, $CDCl_3$) 5.74–5.62 (1H, m, $CH_2CH{=}CH_2$), 4.97–4.89 (2H, m, $CH_2CH{=}CH_2$), 4.87 (1H, s, $CHCO_2Me$), 3.75 (3H, s, CO_2Me), 3.74 (3H, s, CO_2Me), 2.17–2.07 (1H, m, $CH_2CHHCH{=}CH_2$), 2.01–1.90 (1H, m, $CH_2CHHCH{=}CH_2$), 1.84 (1H, ddd, *J* 13.5, 11.5 and 5, $CHHCH_2CH{=}CH_2$), 1.66 (1H, ddd, *J* 13.5, 11.5 and 5, $CHHCH_2CH{=}CH_2$), 1.54 (3H, s, one CH_3 of $C(CH_3)_2$), 1.37 (3H, s, one CH_3 of $C(CH_3)_2$); δ_C(100 MHz, $CDCl_3$) 172.3 (CO_2Me), 168.7 (CO_2Me), 137.3 ($CH{=}CH_2$), 115.1 ($CH{=}CH_2$), 112.6 ($C(CH_3)_2$), 85.4 ($CCH_2CH_2CH{=}CH_2$), 80.1 ($CHCO_2Me$), 52.8 (CO_2Me), 52.3 (CO_2Me), 33.4 ($CH_2CH_2CH{=}CH_2$), 28.1 ($CH_2CH_2CH{=}CH_2$), 27.6 ($C(CH_3)_2$), 26.0 ($C(CH_3)_2$); HRMS *m*/*z* [$M+H^+$] found: 273.1332, $C_{13}H_{21}O_6$ requires 273.1333.

(((3-Methylenepent-4-en-1-yl)oxy)methyl)benzene (39).

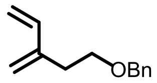

A stirred solution of dimethyl (*R*,*R*)-tartrate acetonide (**7**) (6.90 g, 31.6 mmol, 2 equiv.), iodide **37b** (5.00 g, 15.8 mmol, 1 equiv) and freshly distilled HMPA (21.2 mL) in THF (50 mL) was cooled to –78 °C. To this reaction mixture was added dropwise a pre-cooled (–78 °C) solution of LDA (prepared from iPr_2NH (3.30 g, 31.6 mmol) and *n*-BuLi (20.3 mL, 1.6 M in hexanes, 31.6 mmol) in THF (50 mL) at 0 ºC) over 1 h. The reaction mixture was further stirred for 48 h at –78 °C, and then quenched at the same temperature with sat. aq NH_4Cl (100 mL), extracted with EtOAc (3 × 100 mL),

washed with sat. aq $CuSO_4$ (100 mL) and the combined organic layers were dried ($MgSO_4$), filtered, evaporated under reduced pressure and purified by column chromatography (40% Et_2O in petrol). First eluted diene **39** (1.07g, 36%) as a colourless liquid. R_f 0.54 (10% Et_2O in petrol); ν_{max}/cm^{-1}(neat) 3087 w, 3030 w, 2857 m, 1595 m, 1495 m, 1454 s, 1204 s, 1101 s, 897 s, 734 s; ^{1}H NMR (400 MHz; $CDCl_3$) δ 7.39–7.26 (5H, m, ArCH), 6.40 (1H, dd, *J* 18 and 11, CC*H*=CH_2), 5.27 (1H, d, *J* 18, 1H of C=CH_2), 5.10 (3H, d, *J* 11, 1H of C=CH_2 and CH=C*H_2*), 4.55 (2H, s, C*H_2*Ph), 3.65 (2H, t, *J* 7, CH_2OBn), 2.58 (2H, t, *J* 7, C*H_2*CH_2OBn); ^{13}C NMR (100 MHz; $CDCl_3$) δ 143.2 (*C*=CH_2), 138.8 (*C*H=CH_2), 138.6 (ArC), 128.5 (2xArCH), 127.8 (2xArCH), 127.7 (ArCH), 117.2 (C=*C*H_2), 113.6 (CH=*C*H_2), 73.1 (*C*H_2Ph), 69.2 (*C*H_2OBn), 31.8 (*C*$H_2$$CH_2$OBn); HRMS *m*/*z* [M+H$^+$] found: 189.1275, $C_{13}H_{17}O$ requires 189.1273. Second eluted a mixture of alkylated tartrate **38b** with tartrate **7** (~12 g), which was distilled under reduced pressure (0.2 mbar at ~160 °C) to give alkylated tartrate **38b** (3.85 g, 60%) as a colourless oil. Data as lit [7].

Dimethyl (4*R*,5*S*)-2,2-dimethyl-4-(3-methylbut-2-enyl)-1,3-dioxolane-4,5-dicarboxylate (*epi*-33f).

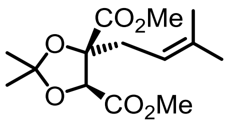

Prenylated tartrate **33f** (25 mg, 0.08 mmol) and NaOMe (5.5 mg, 0.1 mmol) in MeOH (1.3 ml) was heated at reflux for 30 h. H_2O (0.5 mL) was then added and the reaction mixture extracted with Et_2O (2 × 5 mL). The combined organic layers were washed with brine (2 mL), dried (Na_2SO_4) and evaporated under reduced pressured. ^{1}H NMR analysis of the residue indicated 75:25 *epi*-**33f**:**33f**. Purification of the residue by gradient column chromatography (0–10% Et_2O in petrol) gave the epimeric

prenylated tartrate *epi*-**33f** [15] (14.5 mg, 58%) as a colourless liquid. R_f 0.21 (20% Et_2O in petrol); $[\alpha]_D^{25}$ −12.8 (*c* 0.6, $CHCl_3$); ν_{max}/cm^{-1}(neat) 2989 m, 2954 m, 2917 m, 1850 w, 11764 s, 1738 s, 1437 m, 1381 m, 1220 s, 1107 s, 867 w; δ_H(400 MHz, $CDCl_3$) 5.22 (1H, t, *J* 7.5, $CH_2CH{=}C(CH_3)_2$), 4.50 (1H, s, C*H*CO_2Me), 3.80 (3H, s, CO_2Me), 3.71 (3H, s, CO_2Me), 2.75 (1H, dd, *J* 15 and 7, CH*H*CH=$C(CH_3)_2$), 2.63 (1H, dd, *J* 15 and 8, C*H*HCH=$C(CH_3)_2$), 1.74 (3H, s, one CH_3 of $C(CH_3)_2$), 1.66 (3H, s, one CH_3 of $C(CH_3)_2$), 1.60 (3H, s, one CH_3 of CH=C(C*H*$_3$)$_2$), 1.40 (3H, s, one CH_3 of CH=C(*CH*$_3$)$_2$); δ_C(100 MHz, $CDCl_3$) 171.8 (*C*O_2Me), 168.2 (*C*O_2Me), 137.3 (CH=*C*$(CH_3)_2$), 116.8 (*C*H=$C(CH_3)_2$), 111.8 (*C*$(CH_3)_2$), 86.8 (*C*$CH_2CH{=}C(CH_3)_2$), 79.5 (*C*HCO_2Me), 52.7 (CO_2*Me*), 52.6 (CO_2*Me*), 33.6 (*C*H_2CH=$(CH_3)_2$), 26.9 (CH=C(*C*H_3)$_2$), 26.6 (CH=C(*C*H_3)$_2$), 26.2 (C(*C*H_3)$_2$), 18.2(C(*C*H_3)$_2$); HRMS *m*/*z* ($M+H^+$) found: 287.1489, $C_{14}H_{23}O_6$ requires 287.1489.

Dimethyl (4*R*,5*S*)-2,2-dimethyl-4-propyl-1,3-dioxolane-4,5-dicarboxylate (*epi*-33a).

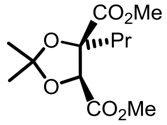

Following the procedure for *epi*-**33f** above, but using propylated tartrate **33a** (56 mg, 0.21 mmol), gave a residue (72:28, *epi*-**33a**:**33a**) which was purified by gradient column chromatography (0–10% Et_2O in petrol), to give *epi*-**33a** (30 mg, 54%) as a colourless liquid. R_f 0.13 (20% Et_2O in petrol); $[\alpha]_D^{25}$ −38.5 (*c* 1.0, $CHCl_3$); ν_{max}/cm^{-1}(neat) 2959 m, 2876 w, 1764 s, 1737 s, 1438 m, 1381 m, 1218 s, 1136 s, 1102 s, 1023 m, 959 w; δ_H(400 MHz, $CDCl_3$) δ 4.45 (1H, s, C*H*CO_2Me), 3.79 (3H, s, CO_2Me),

3.70 (3H, s, CO_2Me), 2.10 (1H, ddd, *J* 14, 12 and 4.5, $CHHCH_2CH_3$), 1.76 (1H, ddd, *J* 14, 12 and 5, $CHHCH_2CH_3$), 1.60 (3H, s, one CH_3 of $C(CH_3)_2$), 1.56–1.45 (1H, m, CH_2CHHCH_3), 1.41 (3H, s, one CH_3 of $C(CH_3)_2$), 1.38–1.28 (1H, m, CH_2CHHCH_3), 0.94 (3H, t, *J* 7.5, CH_2CH_3); δ_c(100 MHz, $CDCl_3$) 171.9 (CO_2Me), 168.1 (CO_2Me), 111.8 ($C(CH_3)_2$), 86.9 ($CCH_2CH_2CH_3$), 80.9 ($CHCO_2Me$), 52.6 (CO_2Me), 52.6 (CO_2Me), 38.4 ($CH_2CH_2CH_3$), 26.9 (2 x $C(CH_3)_2$), 17.5 ($CH_2CH_2CH_3$), 14.3 ($CH_2CH_2CH_3$); HRMS *m/z* ($M+H^+$) found 261.1332, $C_{12}H_{21}O_6$ requires 261.1332.

Dimethyl (4*R*,5*S*)-4-benzyl-2,2-dimethyl-1,3-dioxolane-4,5-dicarboxylate (*epi*-33d)

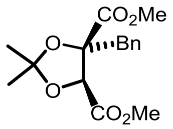

Following the procedure for *epi*-**33f** above, but using benzylated tartrate **33d** (49 mg, 0.16 mmol), gave a residue (83:17, *epi*-**33d**:**33d**) which was purified by gradient column chromatography (0–10% Et_2O in petrol), to give *epi*-**33d** (33 mg, 67%) as a colourless liquid. R_f 0.22 (20% Et_2O in petrol); $[\alpha]_D^{25}$ −4.1 (*c* 1.0, $CHCl_3$); ν_{max}/cm^{-1}(film) 2988 w, 2953 w, 1765 s, 1735 s, 1437 m, 1382 m, 1209 s, 1140 s, 1139 w, 774 w, 701 m; δ_H(400 MHz, $CDCl_3$) 7.37−7.23 (5H, m, ArH), 4.39 (1H, s, $CHCO_2Me$), 3.87 (3H, s, CO_2Me), 3.76 (3H, s, CO_2Me), 3.36 (1H, d, *J* 14, CH*H*Ph), 3.22 (1H, d, *J* 14, C*H*HPh), 1.56 (3H, s, one CH_3 of $C(CH_3)_2$), 1.00 (3H, s, one CH_3 of $C(CH_3)_2$); δ_C(100 MHz, $CDCl_3$) 171.8 (CO_2Me), 168.0 (CO_2Me), 134.5 (quat. ArC), 131.4 (ArC), 128.4 (ArC), 127.4 (ArC), 111.7 ($C(CH_3)_2$), 86.2 (CCH_2Ph), 78.4 ($CHCO_2Me$), 52.8 (CO_2Me), 52.7 (CO_2Me), 39.9 (CH_2Ph), 26.9 ($C(CH_3)_2$), 26.3 ($C(CH_3)_2$); HRMS *m*/*z* [$M+H^+$] found: 309.1333, $C_{16}H_{21}O_6$ requires 309.1332.

Dimethyl (4*R*,5*S*)-2,2-dimethyl-4-(but-3-enyl)-1,3-dioxolane-4,5-dicarboxylate (*epi*-38a).

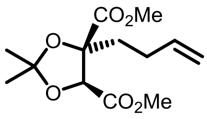

Following the procedure for *epi*-**33f** above, but using butenylated tartrate **38a** (49 mg, 0.18 mmol), gave a residue (75:25, *epi*-**38a**:**38a**) which was purified by gradient column chromatography (0–10% Et_2O in petrol), to give *epi*-**38a** (30 mg, 61%) as a colourless liquid. R_f 0.21 (20% Et_2O in petrol); $[\alpha]_D^{25}$ −24.6 (*c* 1.0, $CHCl_3$); ν_{max}/cm^{-1}(neat) 2985 m, 2954 m, 1767 s, 1736 s, 1438 m, 1382 m, 1247 m, 1210 s, 1107 s, 668 m; δ_H(400 MHz, $CDCl_3$) 5.86–5.74 (1H, m, $CH_2CH{=}CH_2$), 5.08–4.95 (2H, m, $CH_2CH{=}CH_2$), 4.48 (1H, s, $CHCO_2Me$), 3.79 (3H, s, CO_2Me), 3.71 (3H, s, CO_2Me), 2.31–2.20 (2H, m, $CHHCH_2CH{=}CH_2$ and $CH_2CHHCH{=}CH_2$), 2.13–2.03 (1H, m, $CH_2CHHCH{=}CH_2$), 1.94–1.84 (1H, m, $CHHCH_2CH{=}CH_2$), 1.61 (3H, s, one CH_3 of $C(CH_3)_2$), 1.42 (3H, s, one CH_3 of $C(CH_3)_2$); δ_C(100 MHz, $CDCl_3$) 171.6 (CO_2Me), 168.0 (CO_2Me), 137.3 ($CH{=}CH_2$), 115.4 ($CH{=}CH_2$), 112.0 ($C(CH_3)_2$), 86.4 ($CCH_2CH_2CH{=}CH_2$), 80.9 ($CHCO_2Me$), 52.7 (CO_2Me), 52.6 (CO_2Me), 35.5 ($CH_2CH_2CH{=}CH_2$), 28.3 ($CH_2CH_2CH{=}CH_2$), 26.9 ($C(CH_3)_2$), 26.9 ($C(CH_3)_2$); HRMS *m*/*z* ($M+H^+$) found: 273.1332, $C_{13}H_{21}O_6$ requires 273.1332.

References

1. Pangborn, A. B.; Giardello, M. A.; Grubbs, R. H.; Rosen, R. K.; Timmers, F. J. *Organometallics* **1996,** *15*, 1518–1520.

2. Fegheh-Hassanpour, Y.; Ebrahim, F.; Arif, T.; Sintim, H. O.; Claridge, T. D. W.; Amin, N. T.; Hodgson, D. M. *Org. Biomol. Chem.* **2018,** *16*, 2876–2884.

3. Snider, B. B.; Shi, Z. *J. Am. Chem. Soc*. **1994,** *116*, 549–557.

4. Louwrier, S.; Ostendorf, M.; Boom, A.; Hiemstra, H.; Speckamp, W. N. *Tetrahedron* **1996,** *52*, 2603–2628.

5. Baggelaar, M. P.; Huang, Y.; Feringa, B. L; Dekker, F. J.; Minnaard, A. J. *Bioorg. Med. Chem*. **2013**, *21*, 5271–5274.

6. Vedejs, E.; Engler, D. A.; Telschow, J. E. *J. Org. Chem*. **1978,** *43*, 188–196.

7. Fegheh-Hassanpour, Y.; Arif, T.; Sintim, H. O.; Al-Mamari, H. A.; Hodgson, D. M. *Org. Lett.* **2017**, *19*, 3540–3543; *corrigendum* **2018**, *20*, 5528.

8. Vinczer, P.; Novak, L.; Szántay, C. *Synth. Commun*. **1984,** *14*, 281–288. See for ^{1}H NMR shift for NH of δ ~12 being indicative of *Z*–hydrazone configuration.

9. Grzywacz, P.; Chiellini, G.; Plum, L. A.; Clagett-Dame, M.; DeLuca, H. F. *J. Med. Chem.* **2010**, *53*, 8642–8649.

10. Hook, J. M. *Synth. Commun*. **1984**, *14*, 83–87.

11. Hodgson, D. M.; Villalonga–Barber, C.; Goodman, J. M.; Pellegrinet, S. C. *Org. Biomol. Chem.* **2010,** *8*, 3975–3984.

12. Naef, R. Chiral enolates, Dissertation No. 7442, ETH Zürich, Switzerland, 1983.

13. Mukhopadhyay, T.; Seebach, D. *Helv. Chim. Acta* **1982**, *65*, 385–391.

14. Molander, G. A.; Harris, C. R. *J. Am. Chem. Soc*. **1996**, *118*, 4059–4071.

15. Zhang, G.-L.; Li, S.-B.; Li, Y.-L. *Acta. Chim. Sin.* **1989,** *47*, 1087–1092.

^{1}H and ^{13}C NMR Spectra

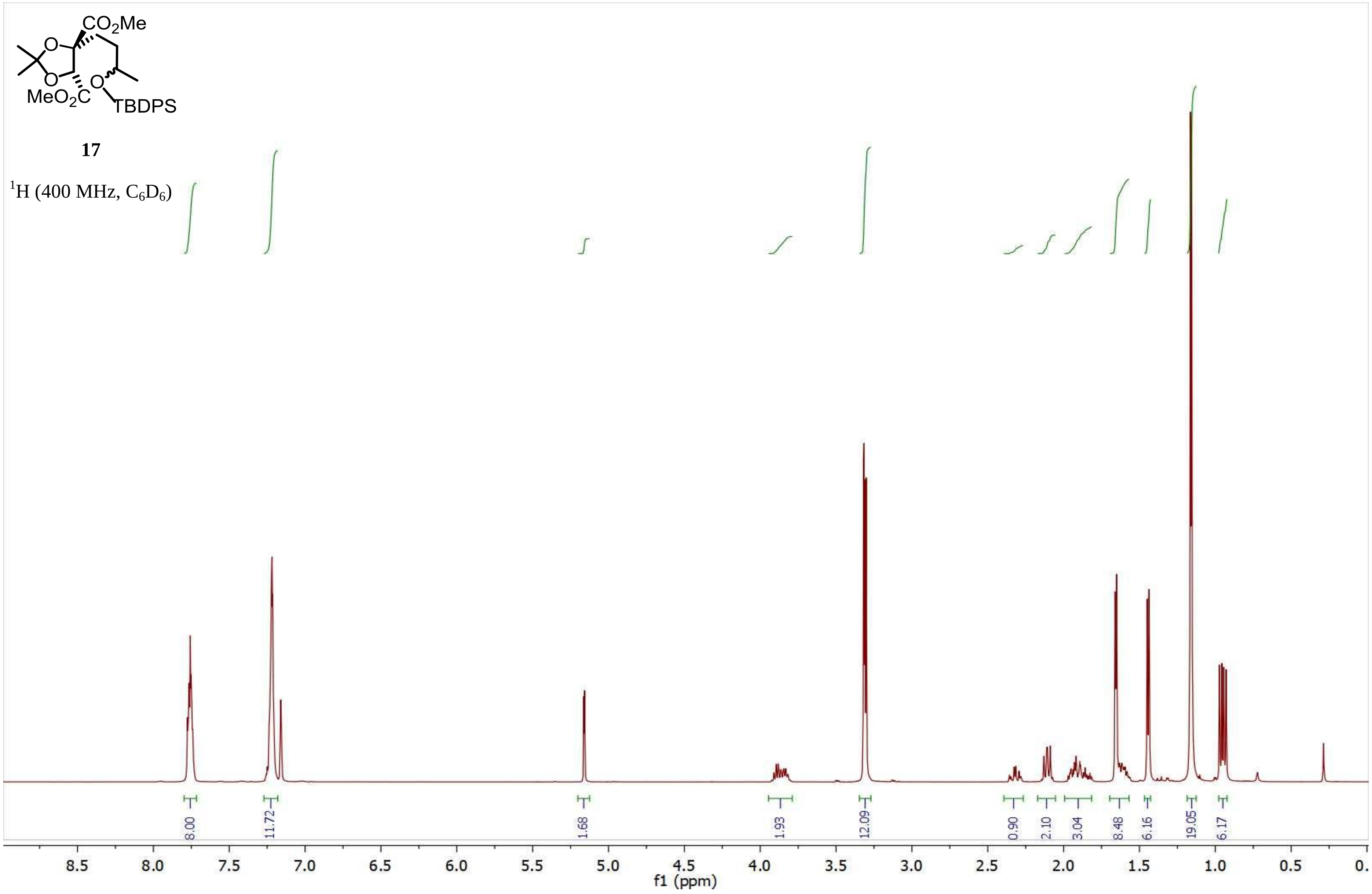

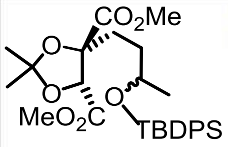

17

^{13}C (100 MHz, C_6D_6)

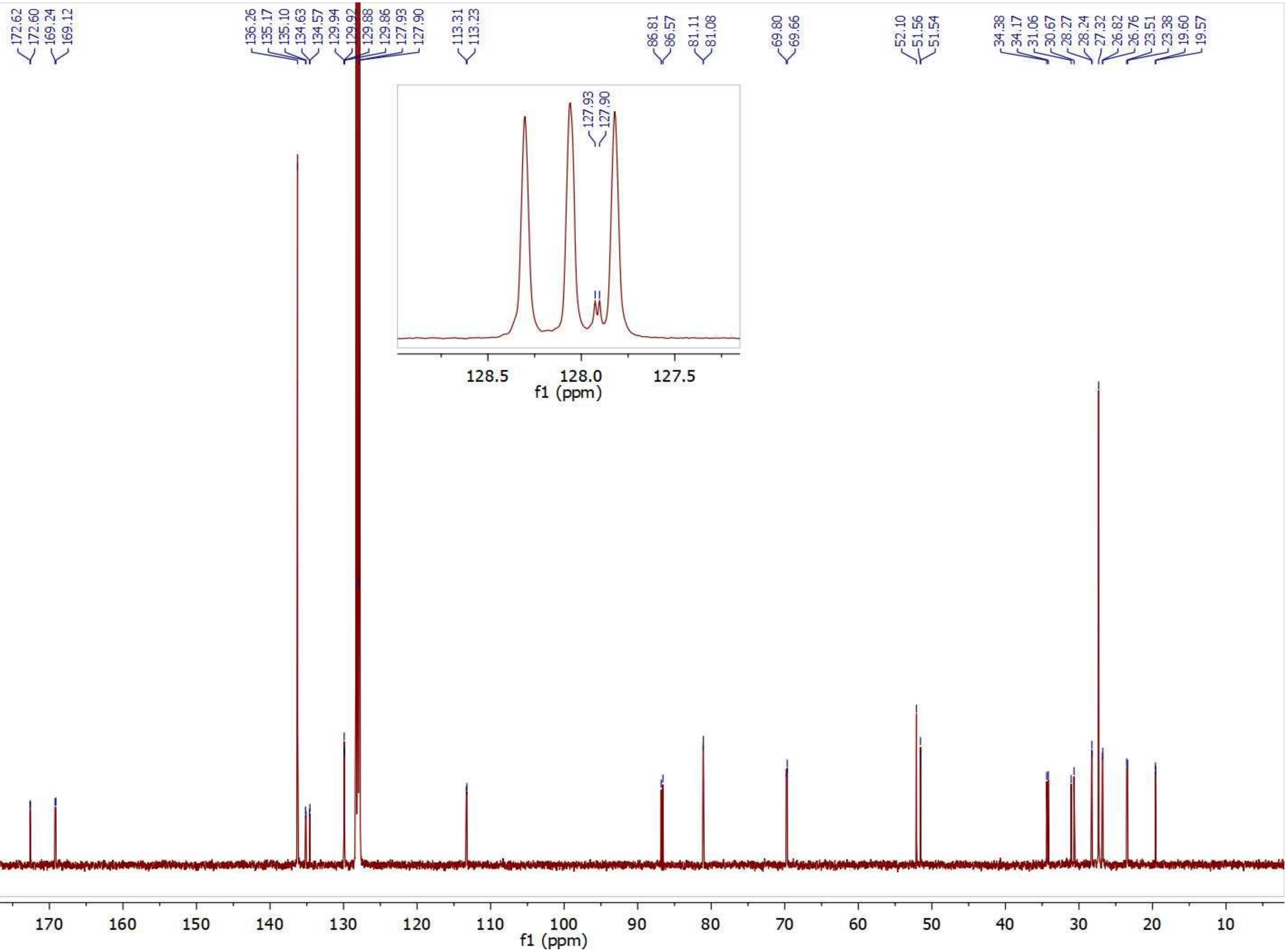

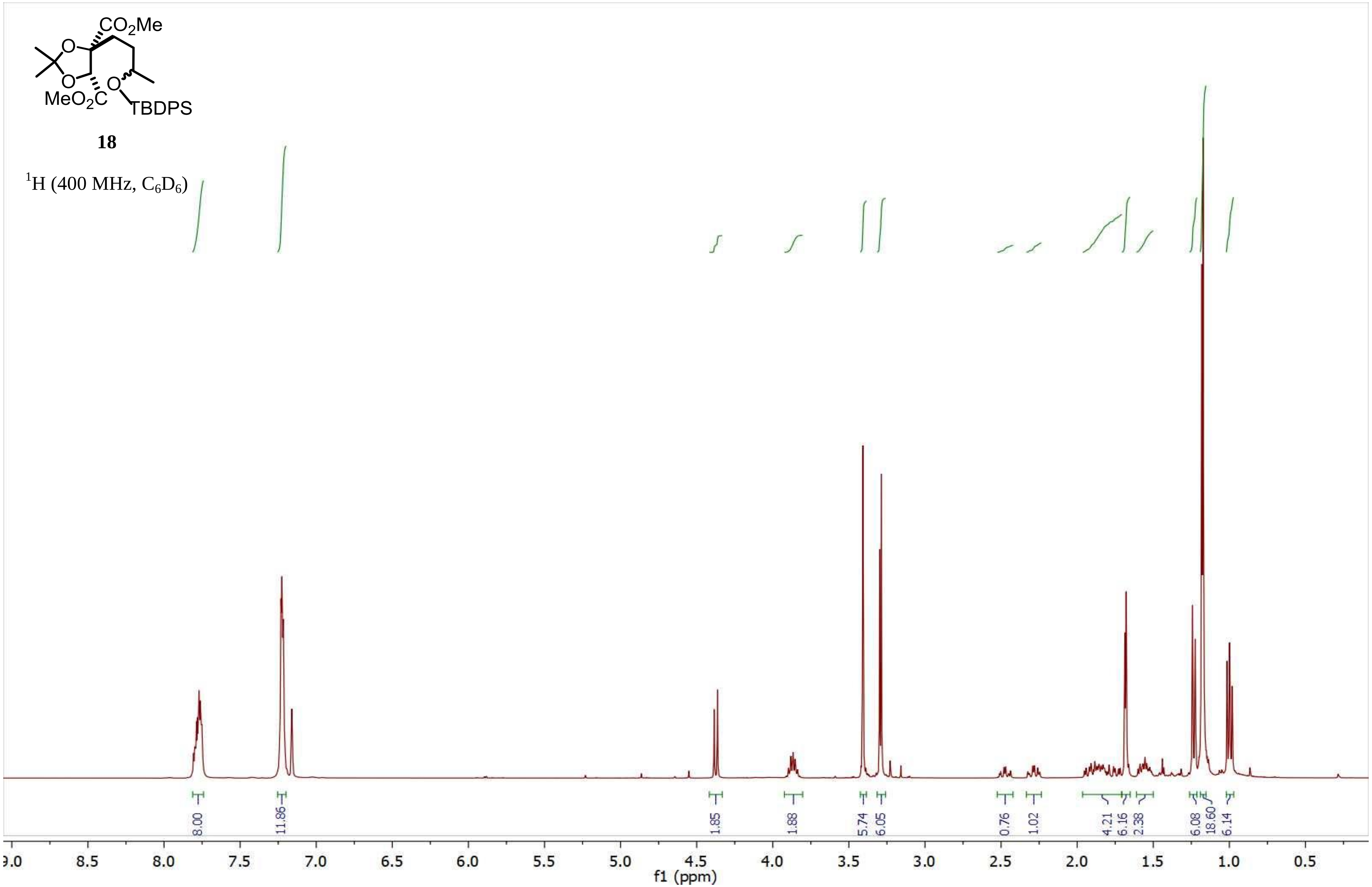
CO2Me
MeO2C
TBDPS
18
1H (400 MHz, C6D6)
8.00
11.86
1.85
1.88
5.74
6.05
0.76
1.02
4.21
6.16
2.38
6.08
18.60
6.14
9.0 8.5 8.0 7.5 7.0 6.5 6.0 5.5 5.0 4.5 4.0 3.5 3.0 2.5 2.0 1.5 1.0 0.5
f1 (ppm)

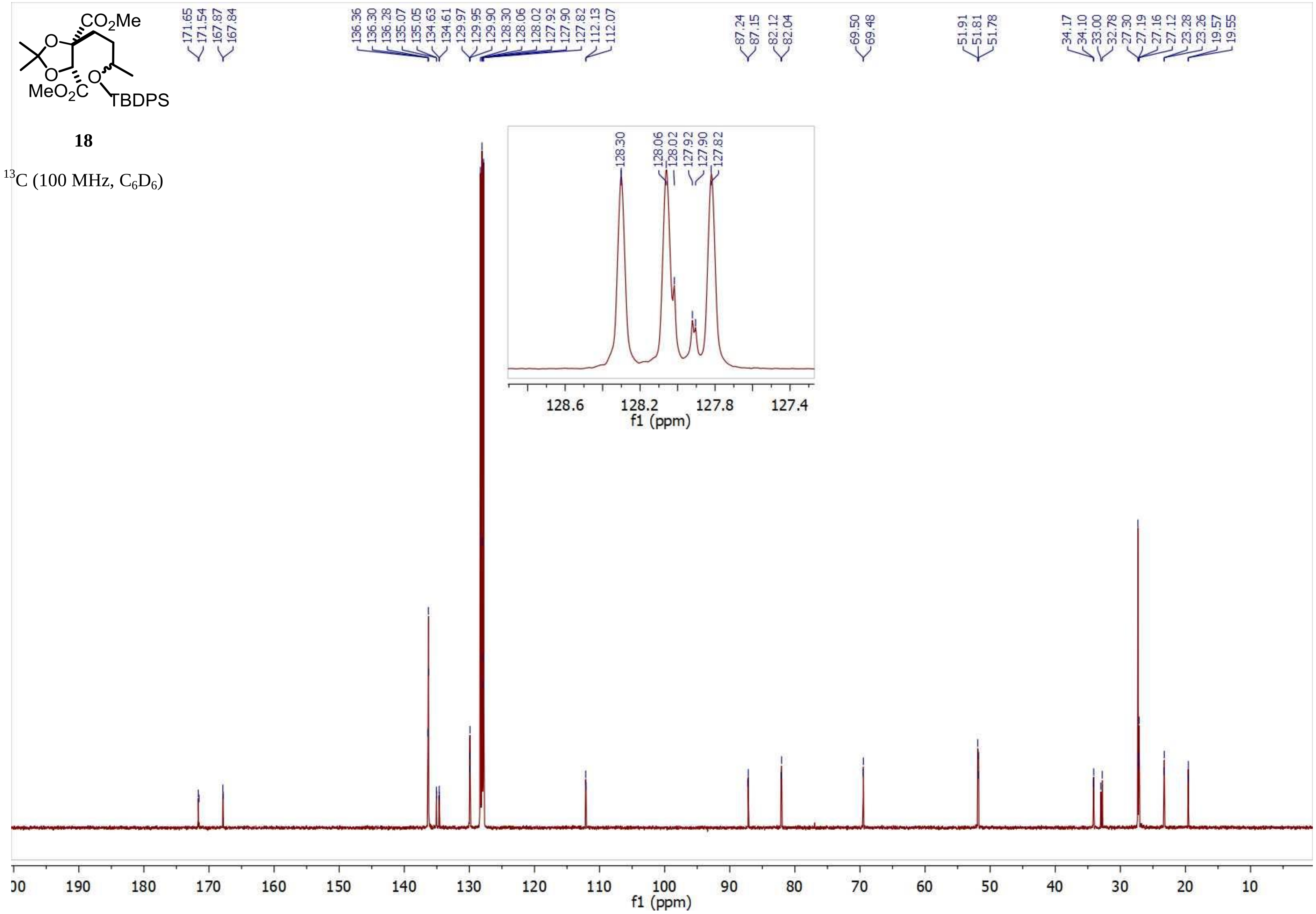

CO2Me
MeO2C
O
TBDPS
18
13C (100 MHz, C6D6)
171.65
171.54
167.87
167.84
136.36
136.30
136.28
135.07
135.05
134.63
134.61
129.97
129.95
129.90
128.30
128.06
128.02
127.92
127.90
127.82
112.13
112.07
87.24
87.15
82.12
82.04
69.50
69.48
51.91
51.81
51.78
34.17
34.10
33.00
32.78
27.30
27.19
27.16
27.12
23.28
23.26
19.57
19.55
128.6
128.2
127.8
127.4
f1 (ppm)
190
180
170
160
150
140
130
120
110
100
90
80
70
60
50
40
30
20
10
f1 (ppm)

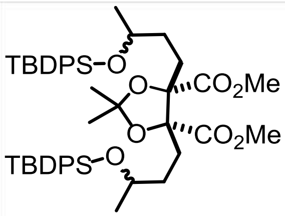

E-1

^{1}H (200 MHz, $CDCl_3$)

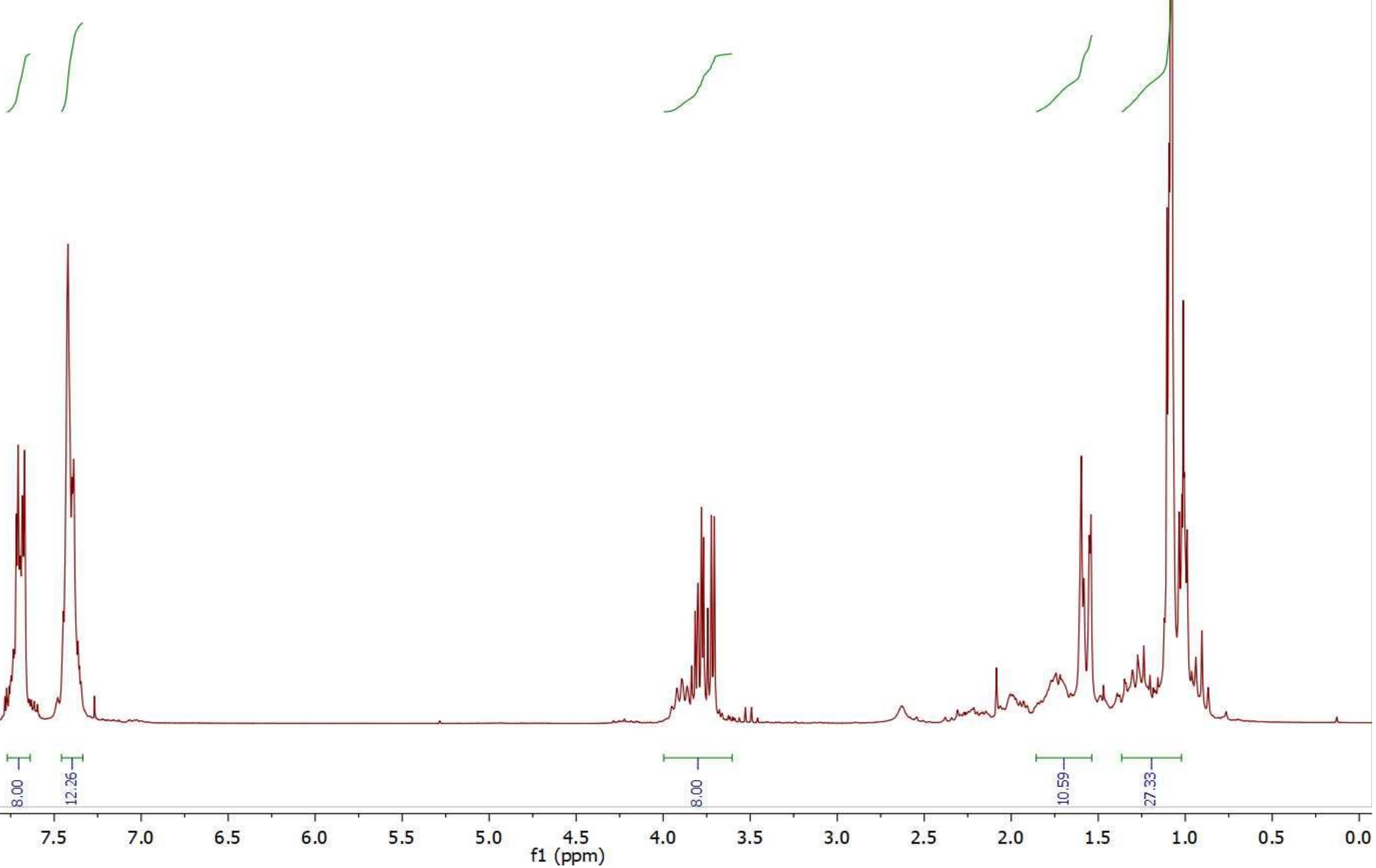

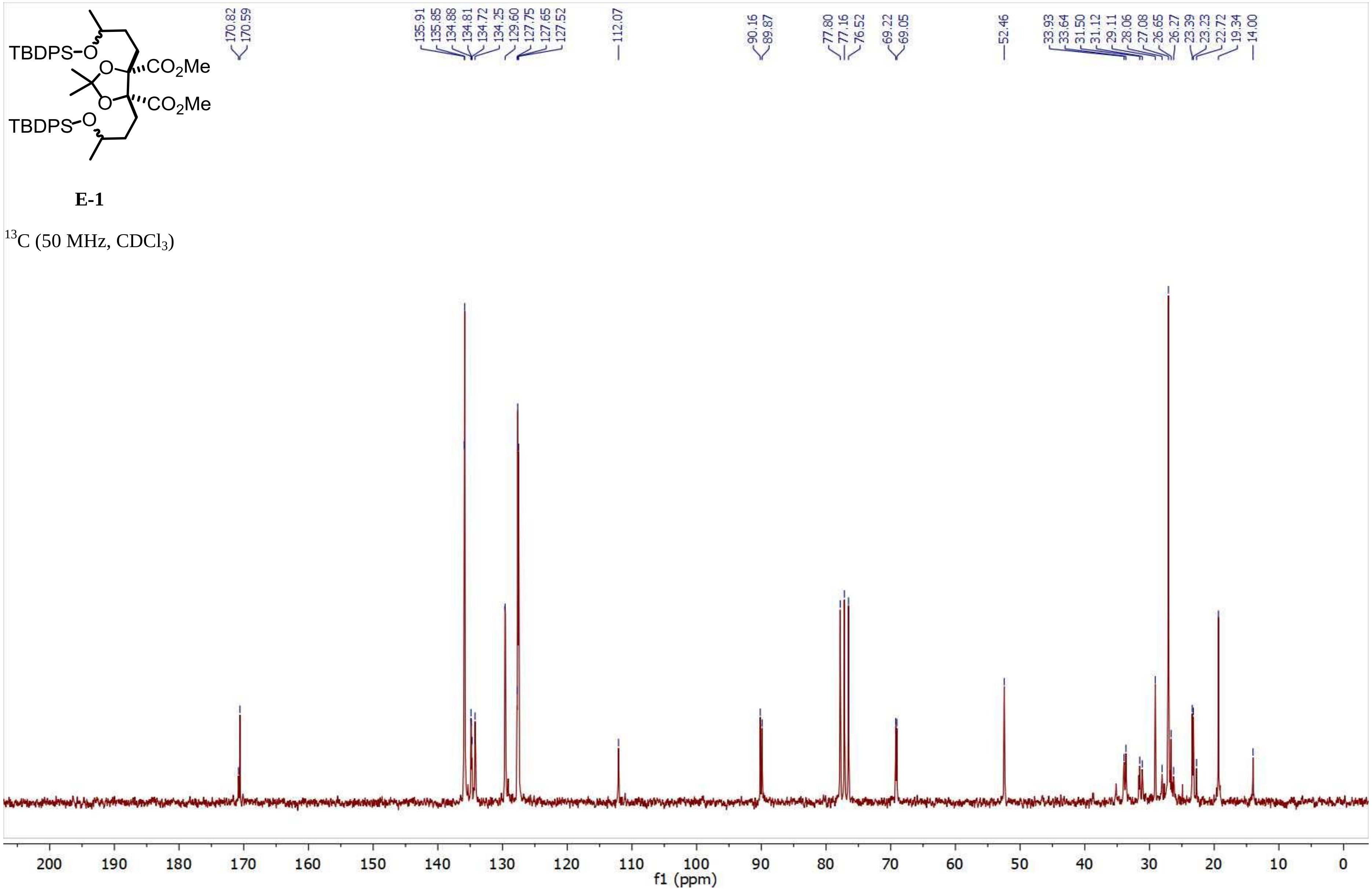

E-1

^{13}C (50 MHz, $CDCl_3$)

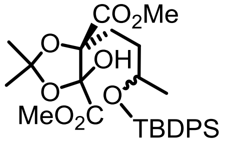

19a

^{1}H (400 MHz, $CDCl_3$)

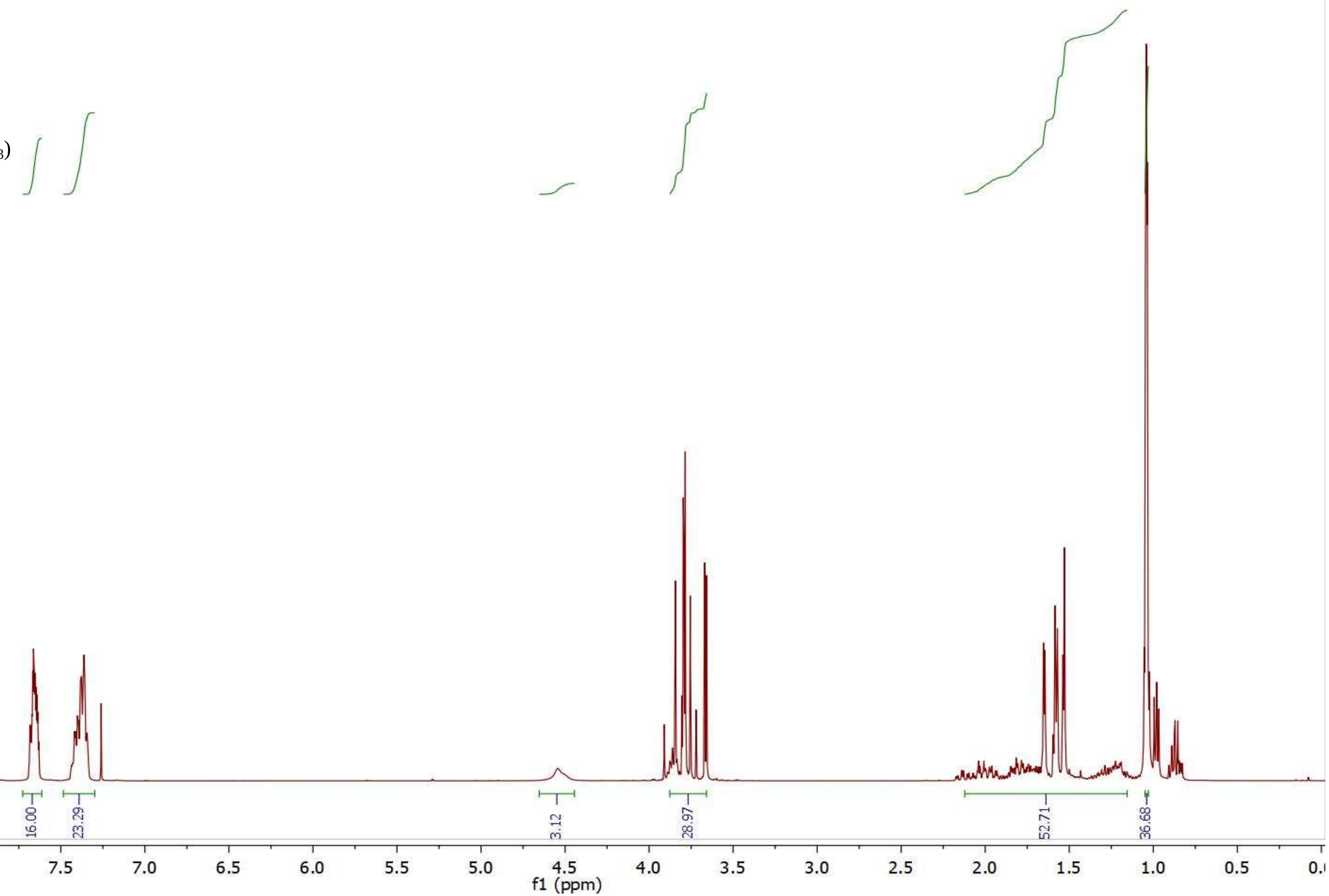

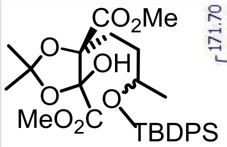

19a

^{13}C (100 MHz, $CDCl_3$)

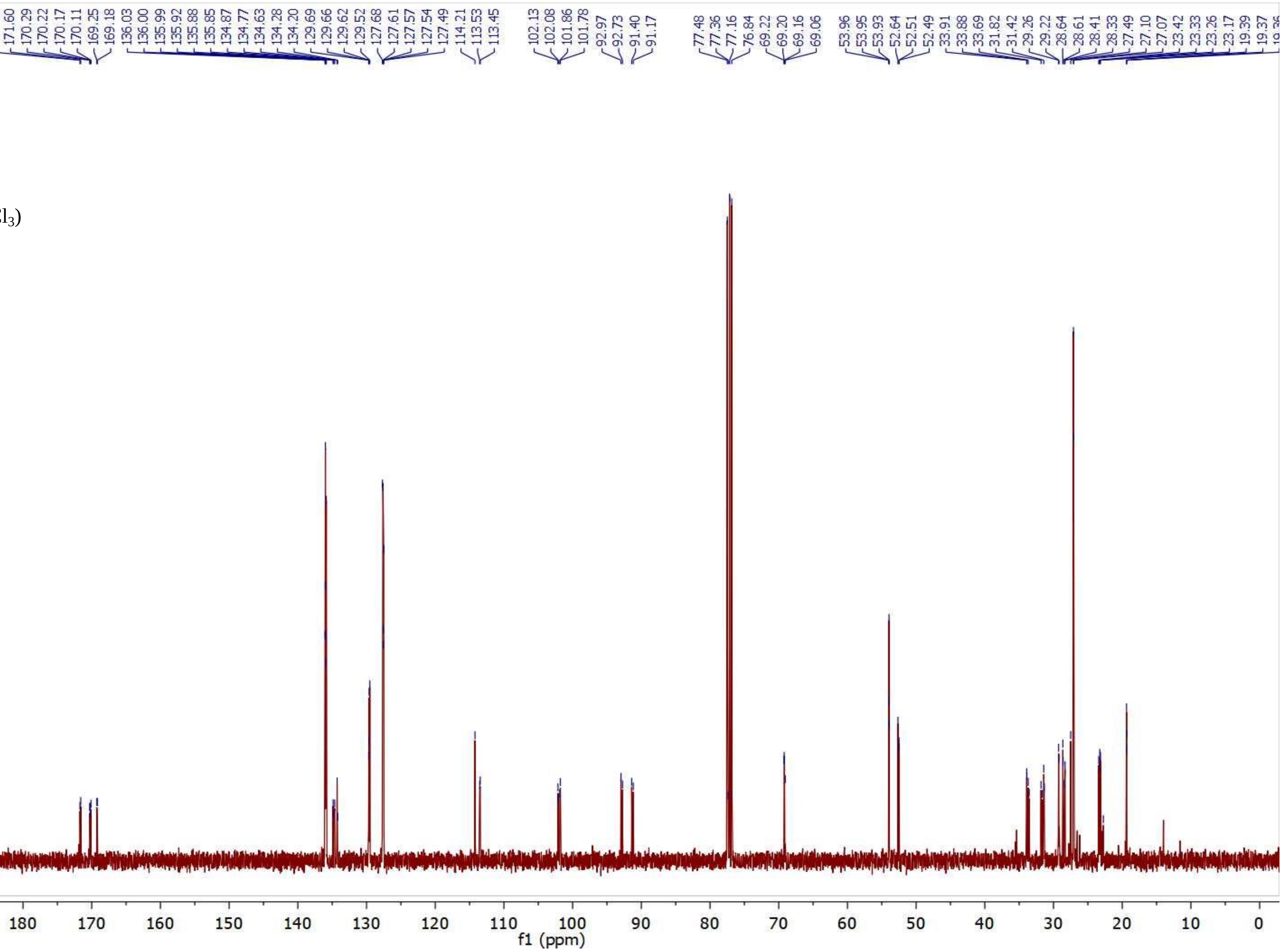

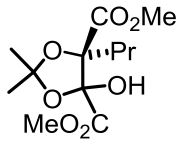

19b

^{1}H (400 MHz, $CDCl_3$)

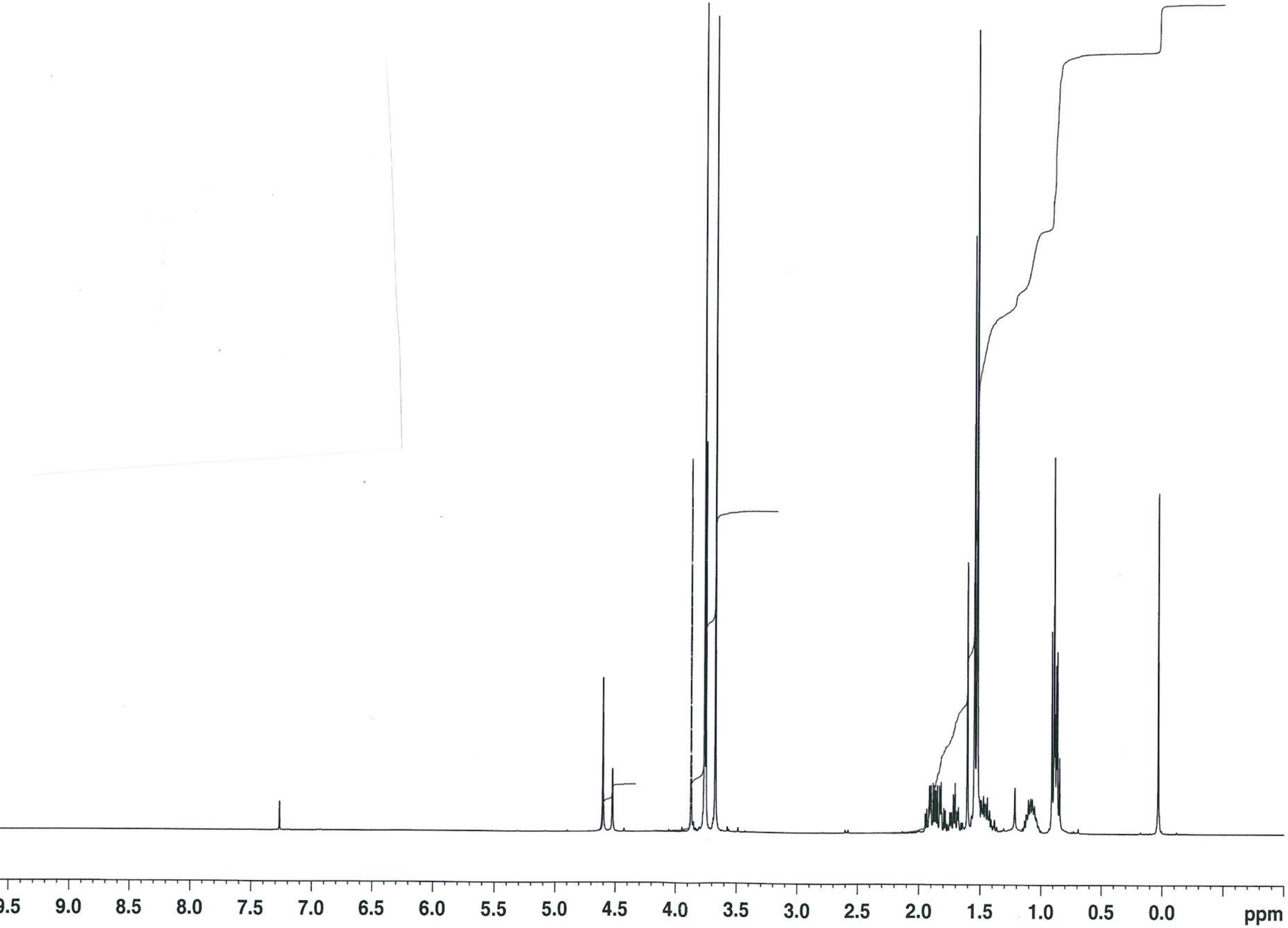

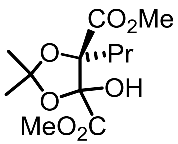

19b

^{13}C (100 MHz, $CDCl_3$)

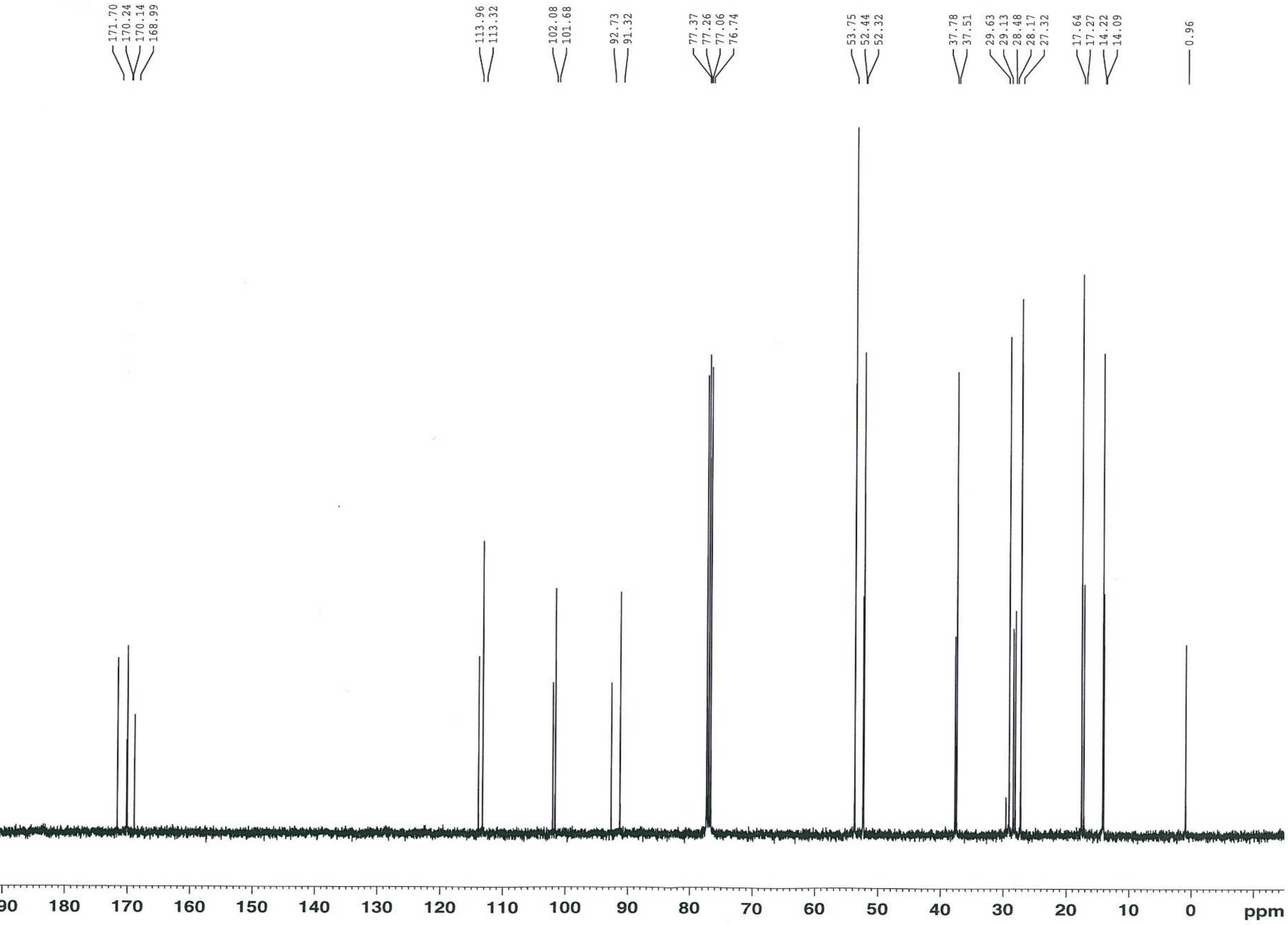

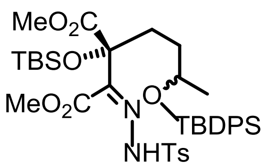

22

^{1}H (400 MHz, $CDCl_3$)

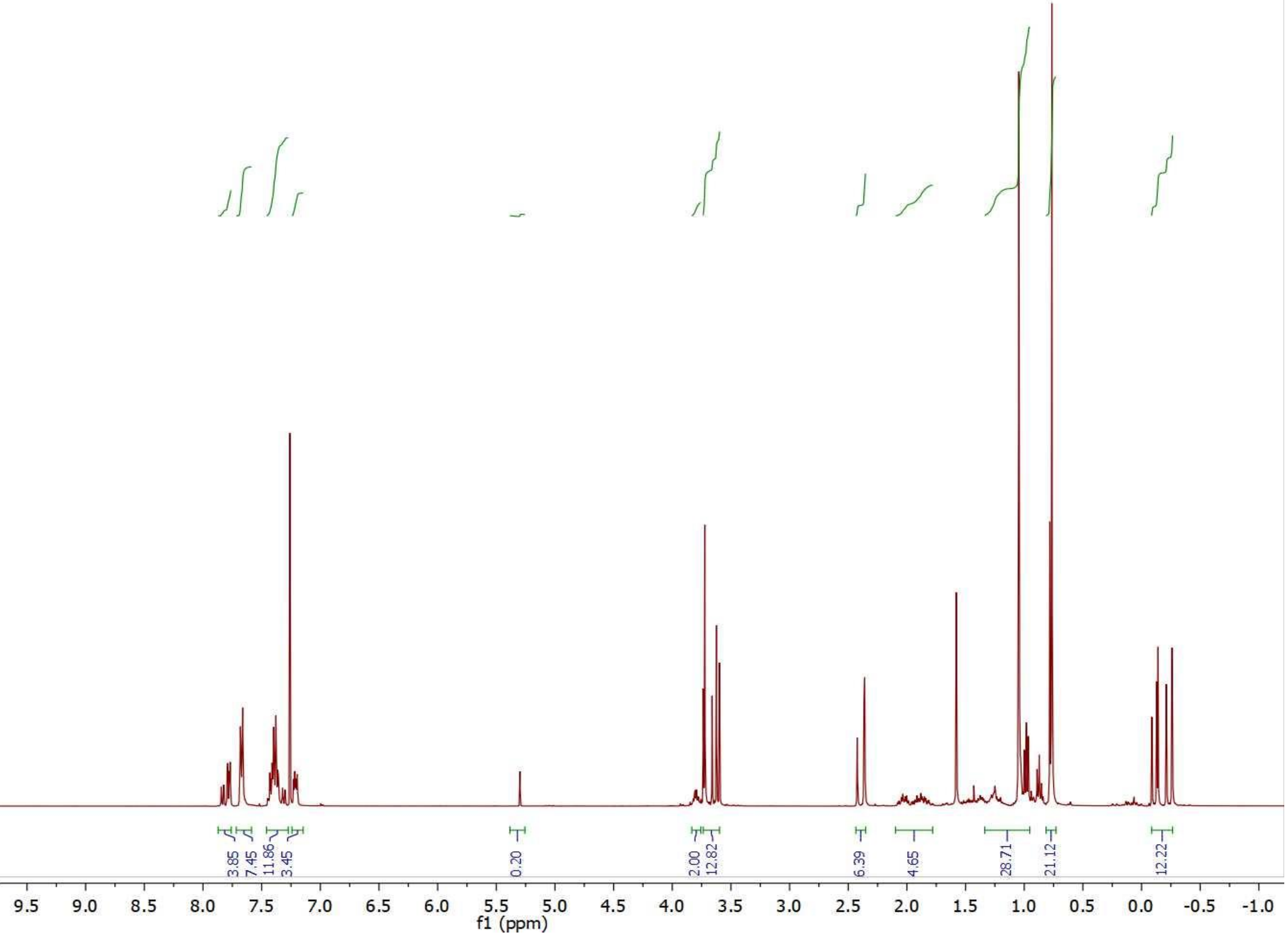

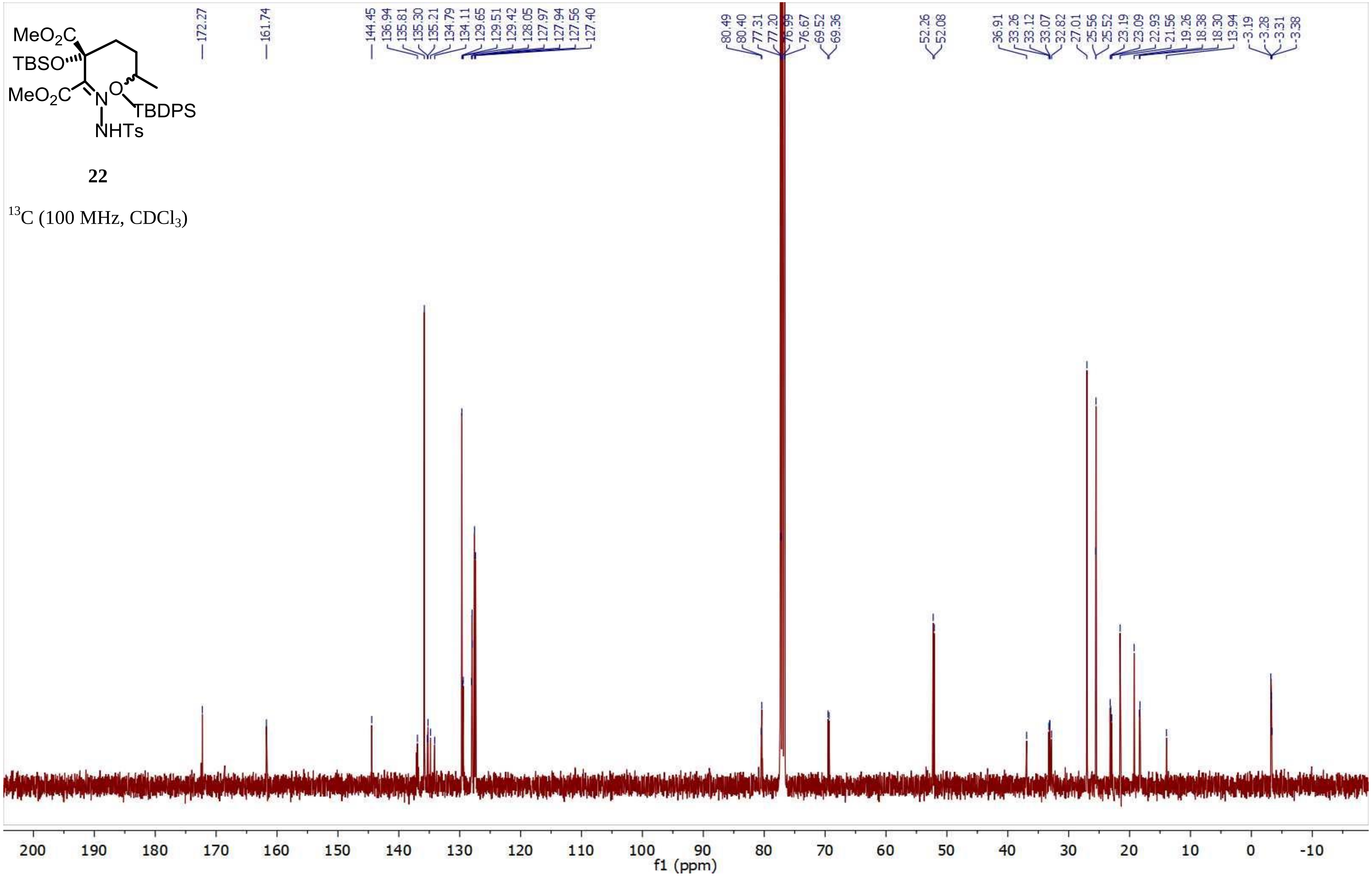

MeO2C
TBSO
MeO2C
N
O
TBDPS
NHTs
22
13C (100 MHz, CDCl3)
172.27
161.74
144.45 136.94 135.81 135.30 135.21 134.79 134.11 129.65 129.51 129.42 128.05 127.97 127.94 127.56 127.40
80.49 80.40 77.31 77.20 76.99 76.67 69.52 69.36
52.26 52.08
36.91 33.26 33.12 33.07 32.82 27.01 25.56 25.52 23.19 23.09 22.93 21.56 19.26 18.38 18.30 13.94 -3.19 -3.28 -3.31 -3.38
200 190 180 170 160 150 140 130 120 110 100 90 80 70 60 50 40 30 20 10 0 -10
f1 (ppm)

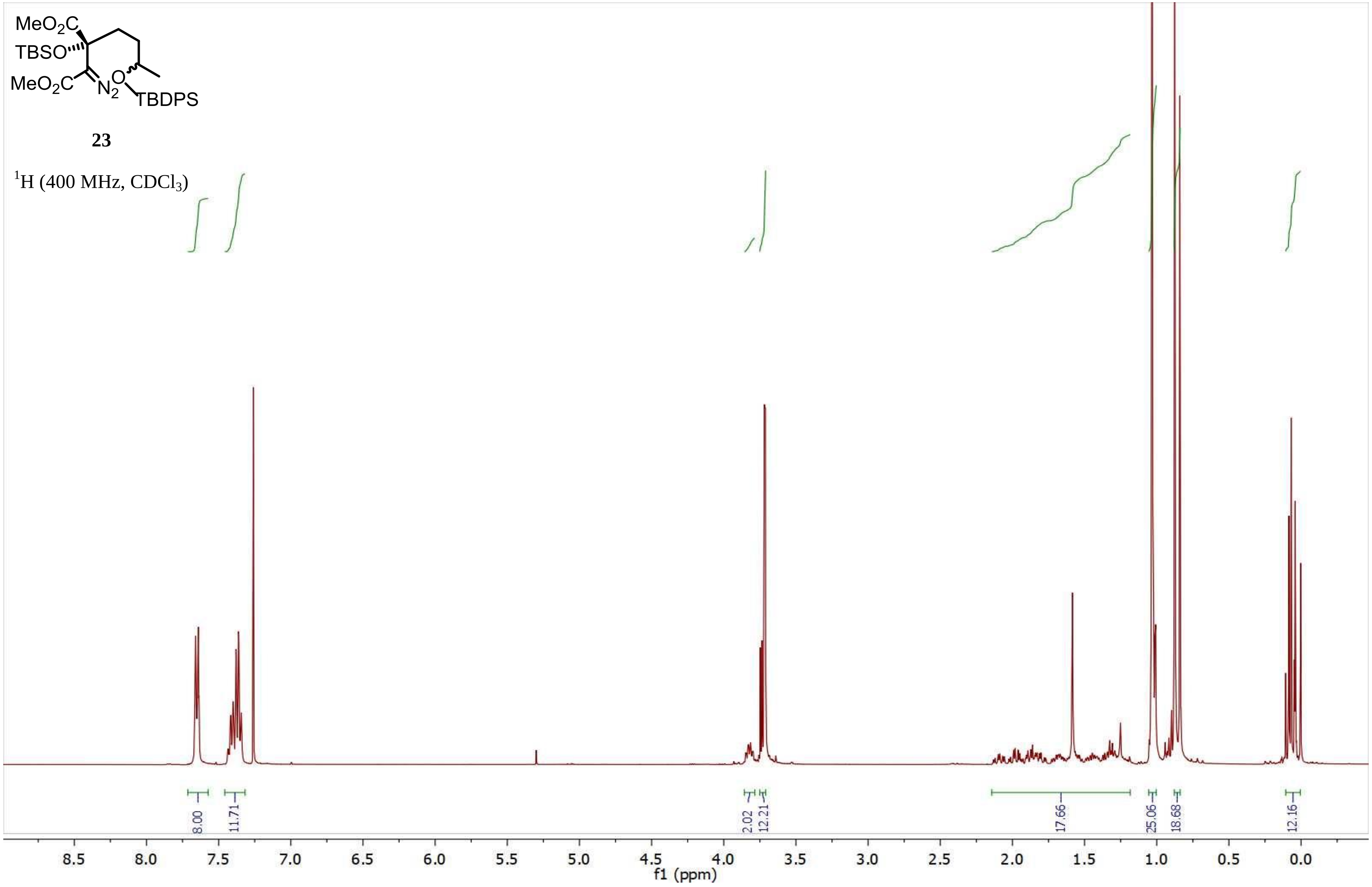
MeO2C
TBSO
MeO2C
N2
O
TBDPS
23
1H (400 MHz, CDCl3)
8.00
11.71
2.02
12.21
17.66
25.06
18.68
12.16
8.5
8.0
7.5
7.0
6.5
6.0
5.5
5.0
4.5
4.0
3.5
3.0
2.5
2.0
1.5
1.0
0.5
0.0
f1 (ppm)

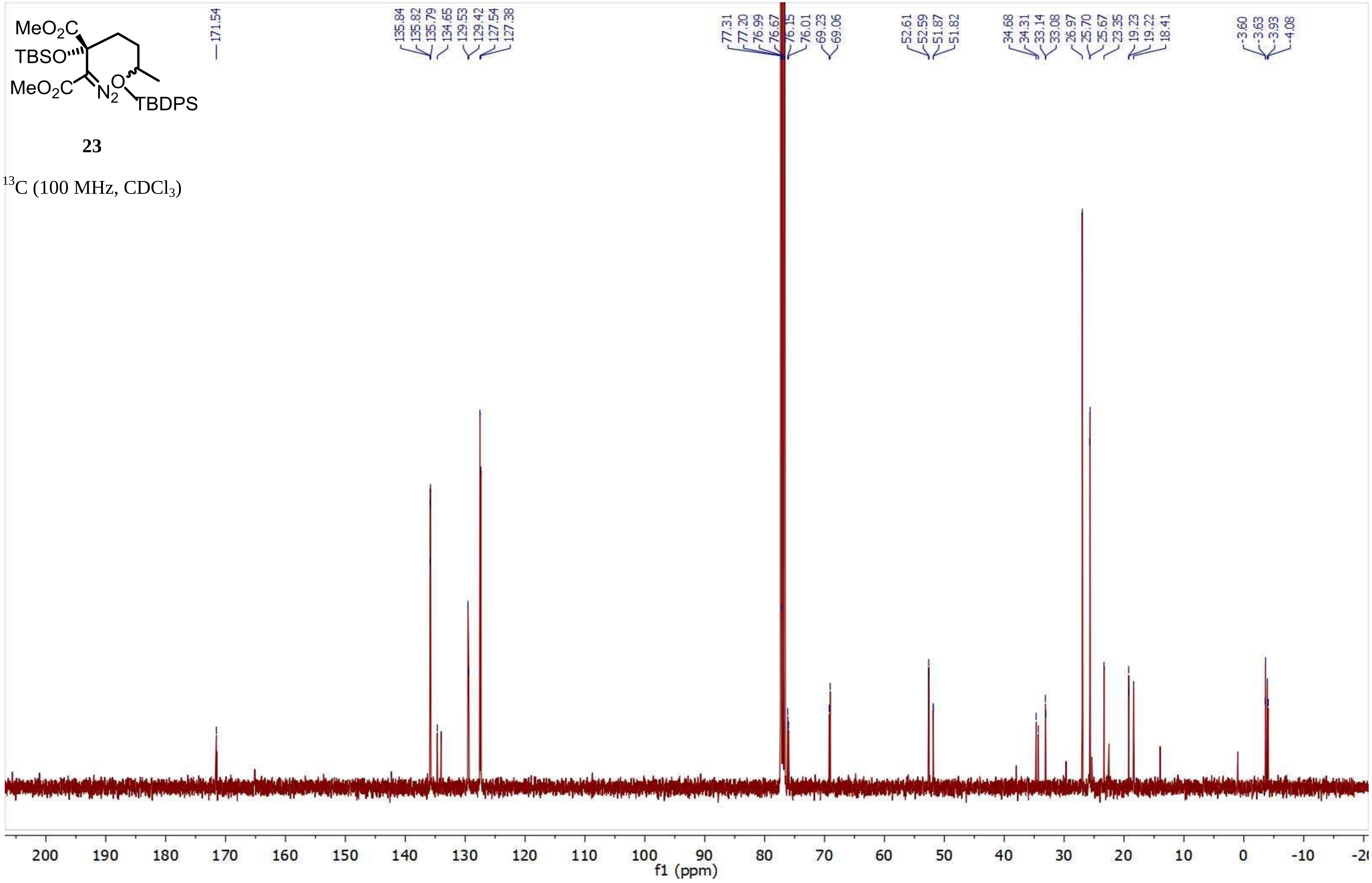

MeO2C
TBSO
MeO2C
N2
O
TBDPS
23
13C (100 MHz, CDCl3)
171.54
135.84
135.82
135.79
134.65
129.53
129.42
127.54
127.38
77.31
77.20
76.99
76.67
76.15
76.01
69.23
69.06
52.61
52.59
51.87
51.82
34.68
34.31
33.14
33.08
26.97
25.70
25.67
23.35
19.23
19.22
18.41
-3.60
-3.63
-3.93
-4.08
f1 (ppm)

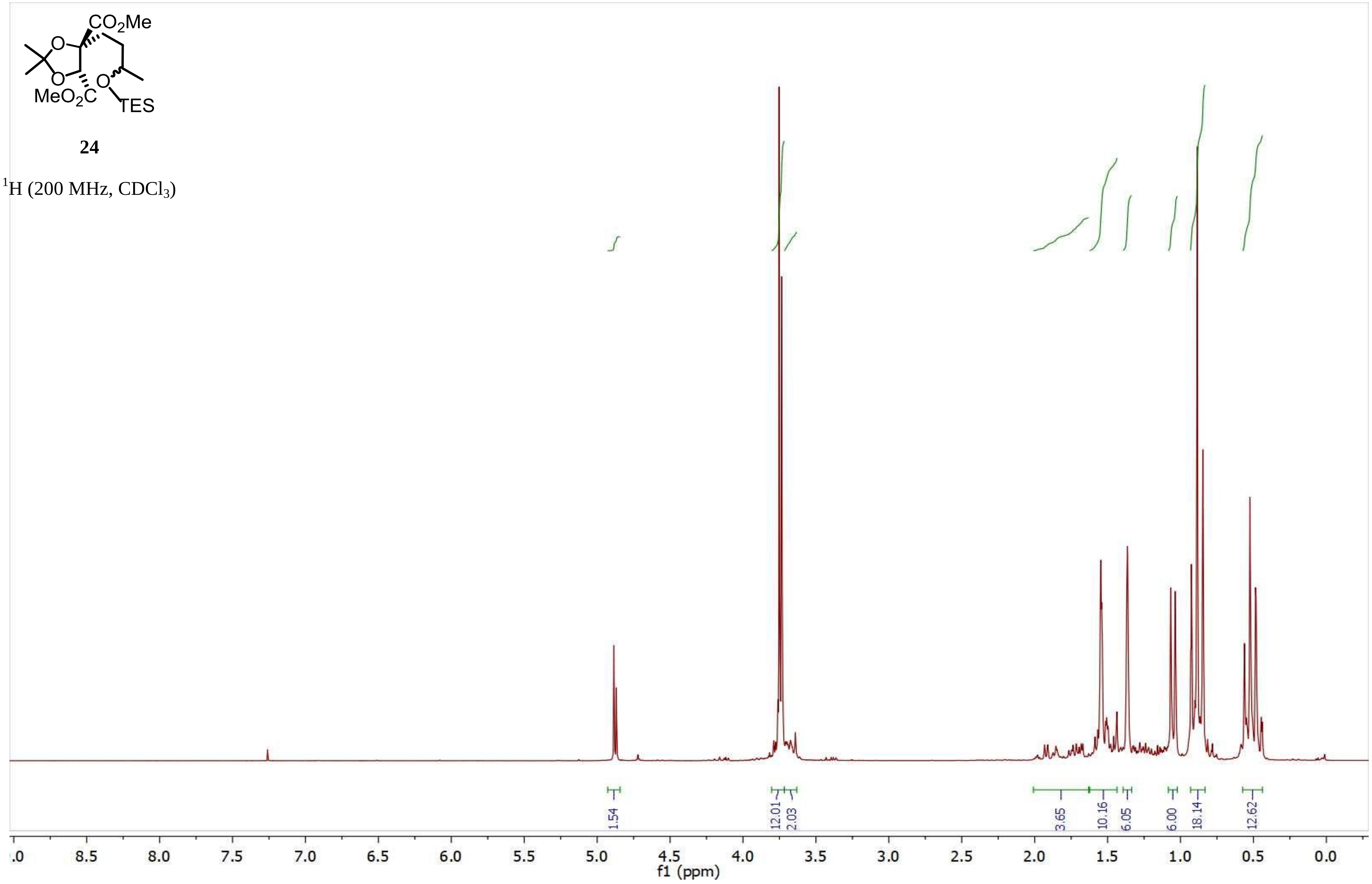

CO2Me
MeO2C
O
O
TES
24
1H (200 MHz, CDCl3)
1.54
12.01
2.03
3.65
10.16
6.05
6.00
18.14
12.62
.0
8.5
8.0
7.5
7.0
6.5
6.0
5.5
5.0
4.5
4.0
3.5
3.0
2.5
2.0
1.5
1.0
0.5
0.0
f1 (ppm)

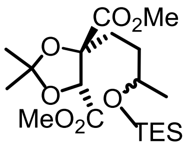

24

^{13}C (50 MHz, $CDCl_3$)

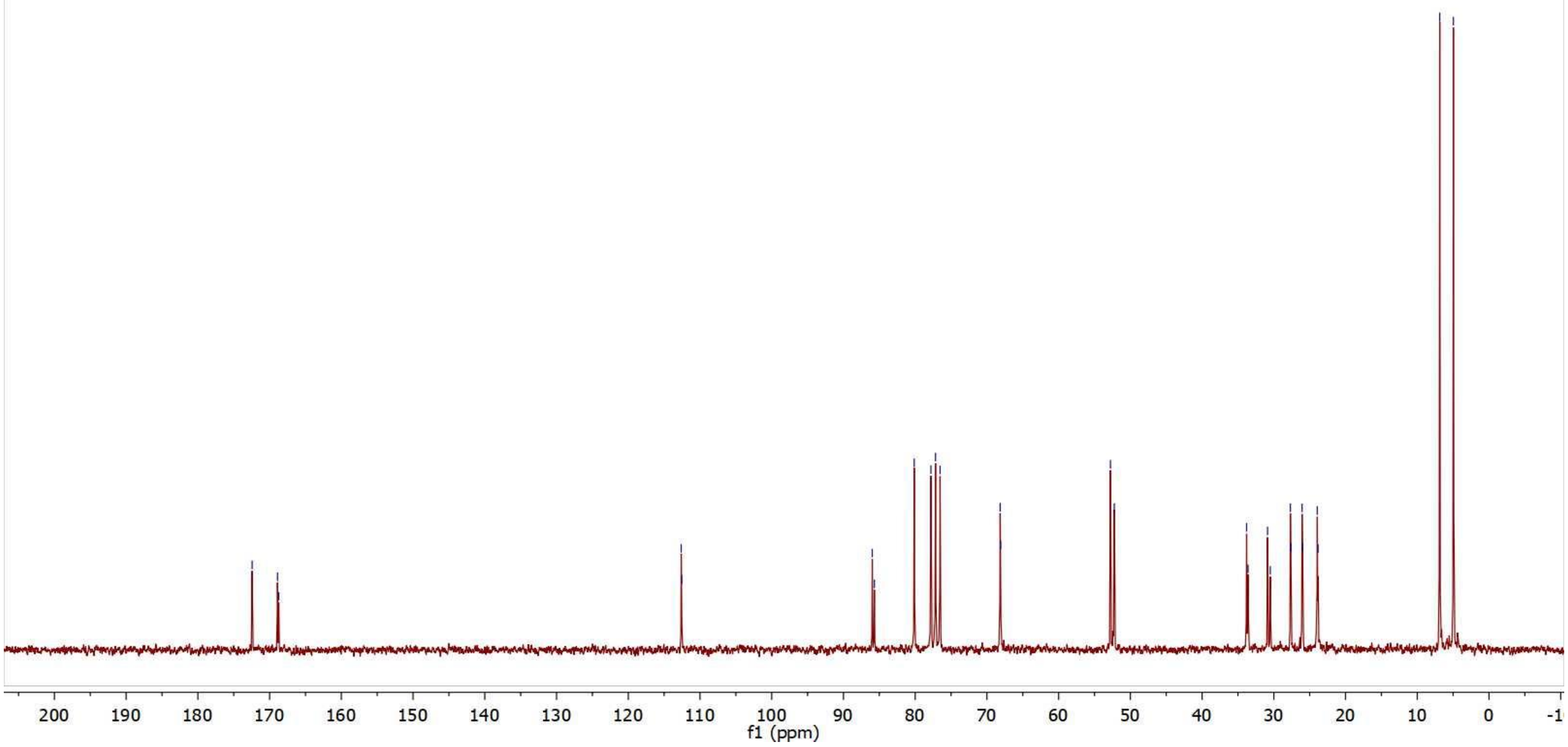

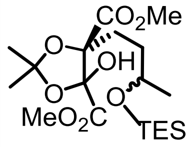

25

^{1}H (400 MHz, $CDCl_3$)

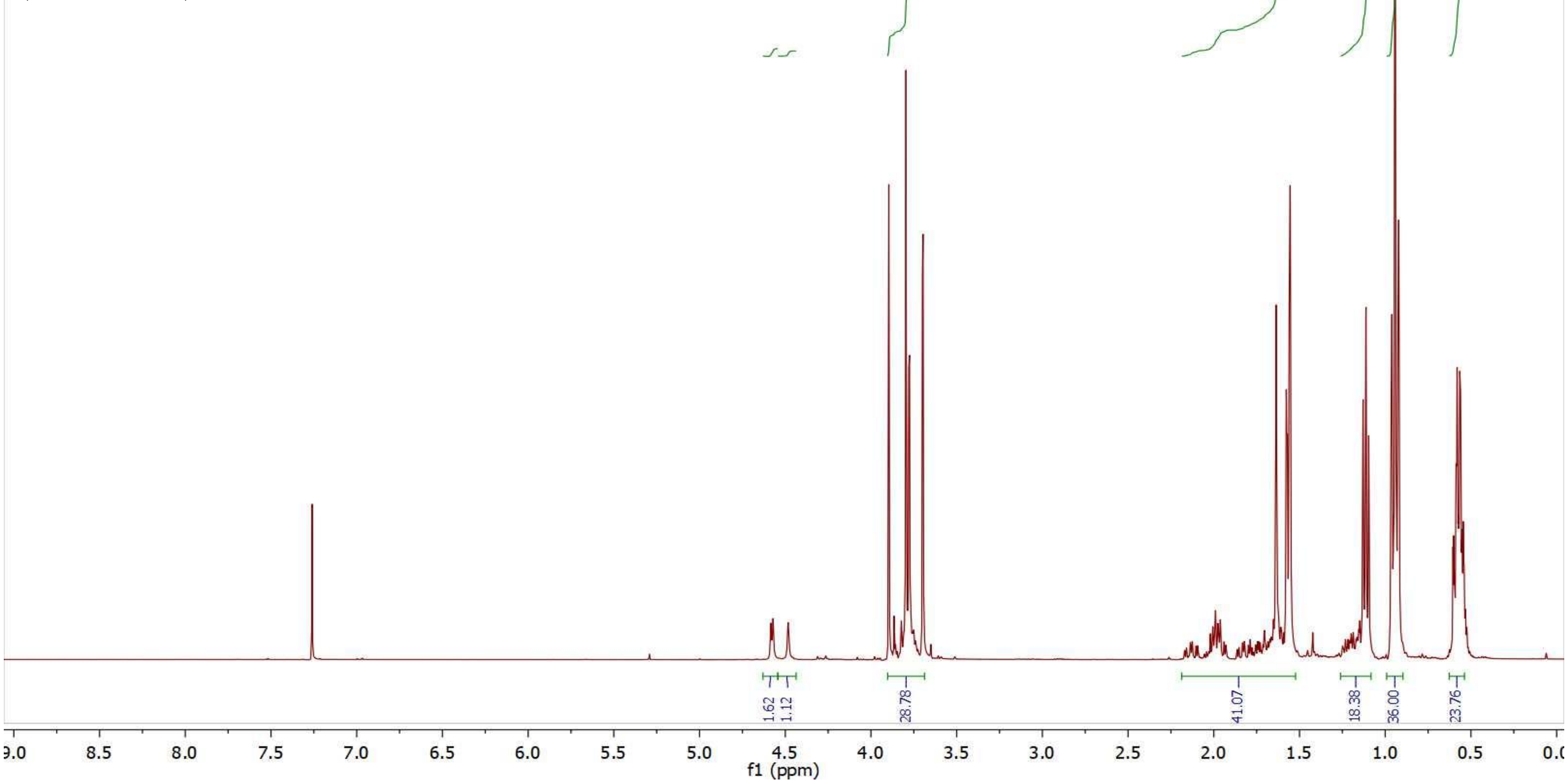

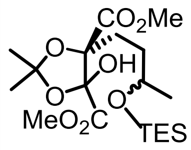

25

^{13}C (100 MHz, $CDCl_3$)

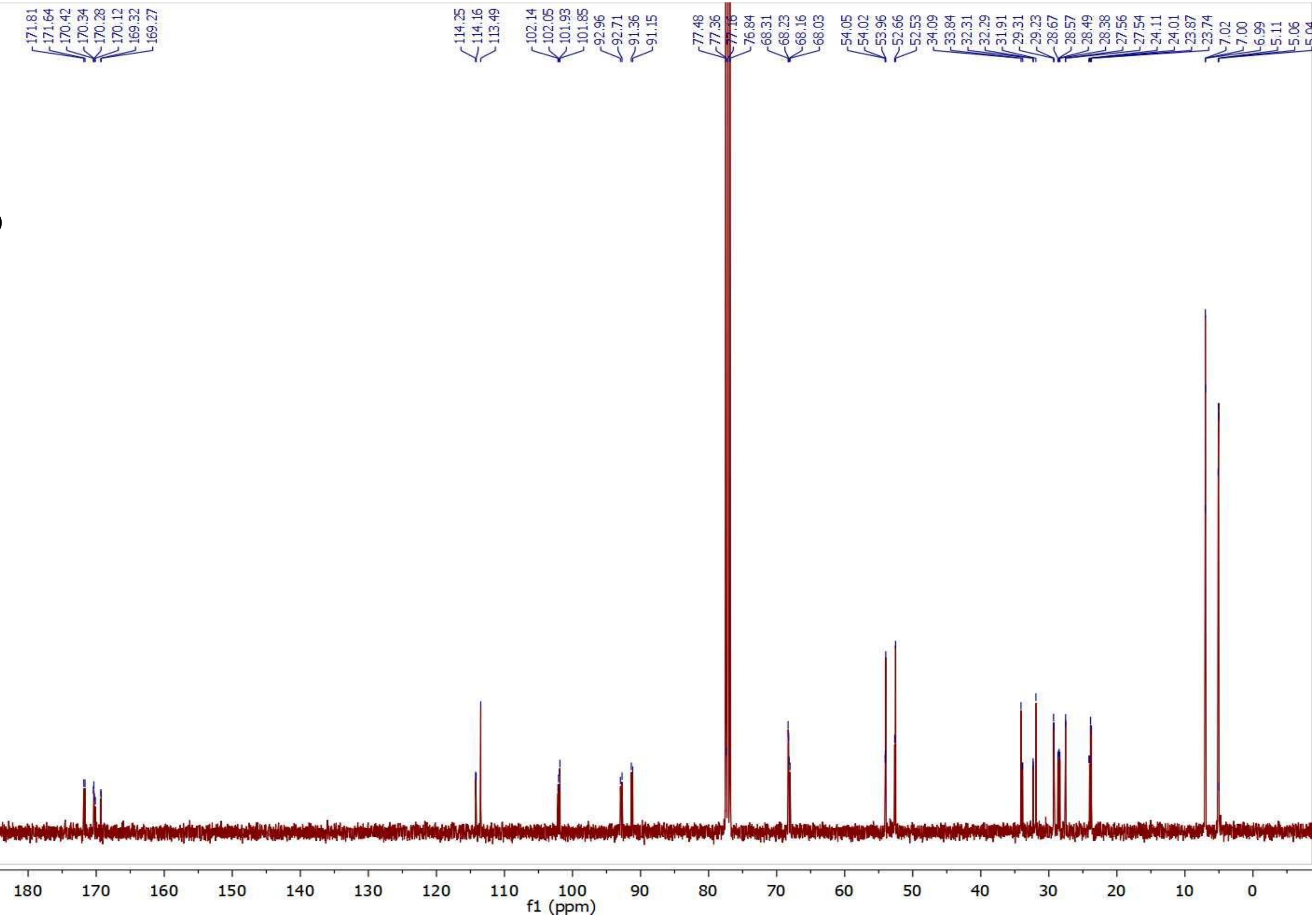

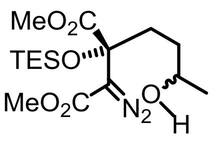

27

^{1}H (400 MHz, $CDCl_3$)

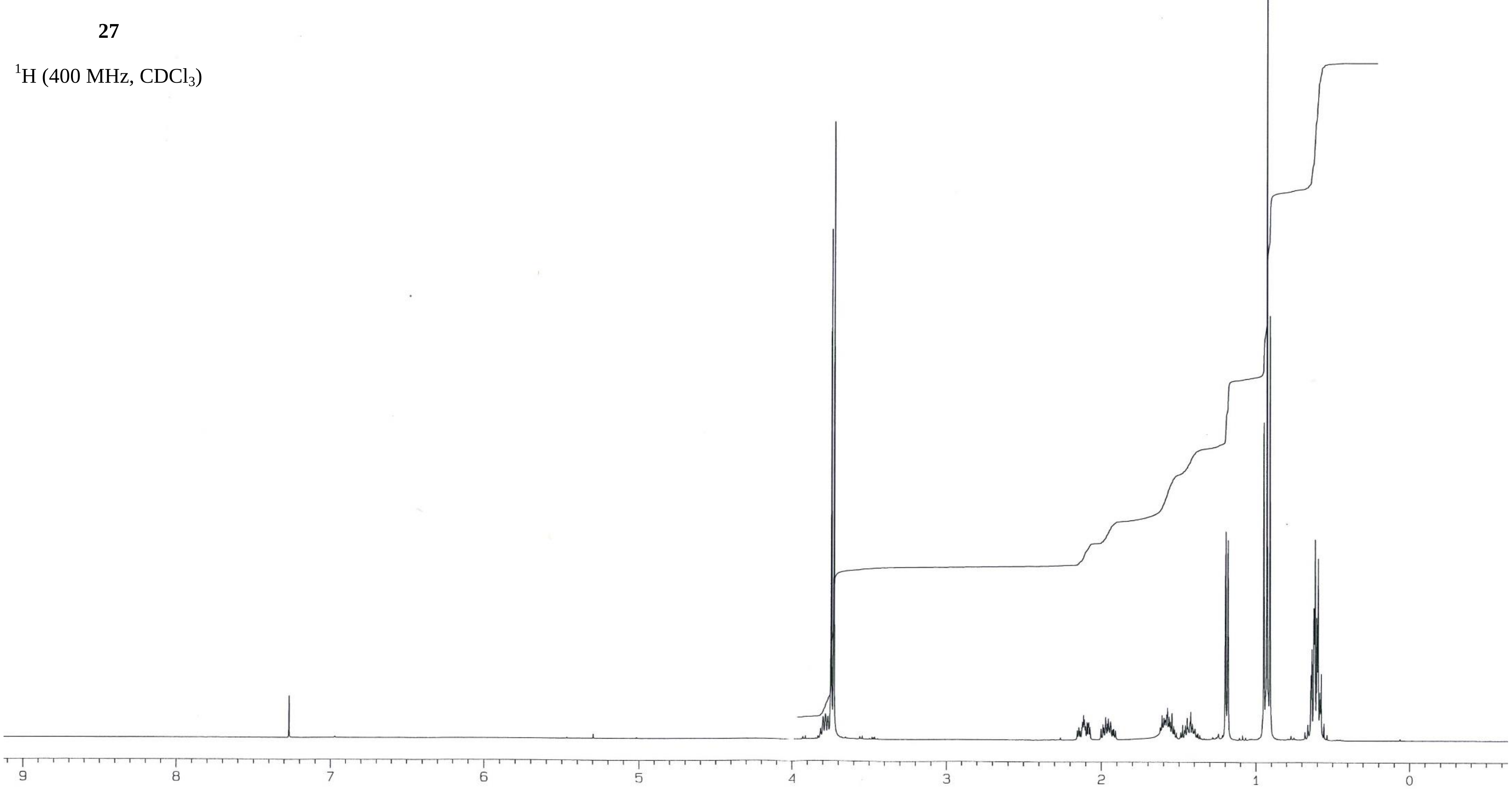

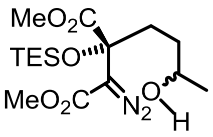

27

^{13}C (100 MHz, $CDCl_3$)

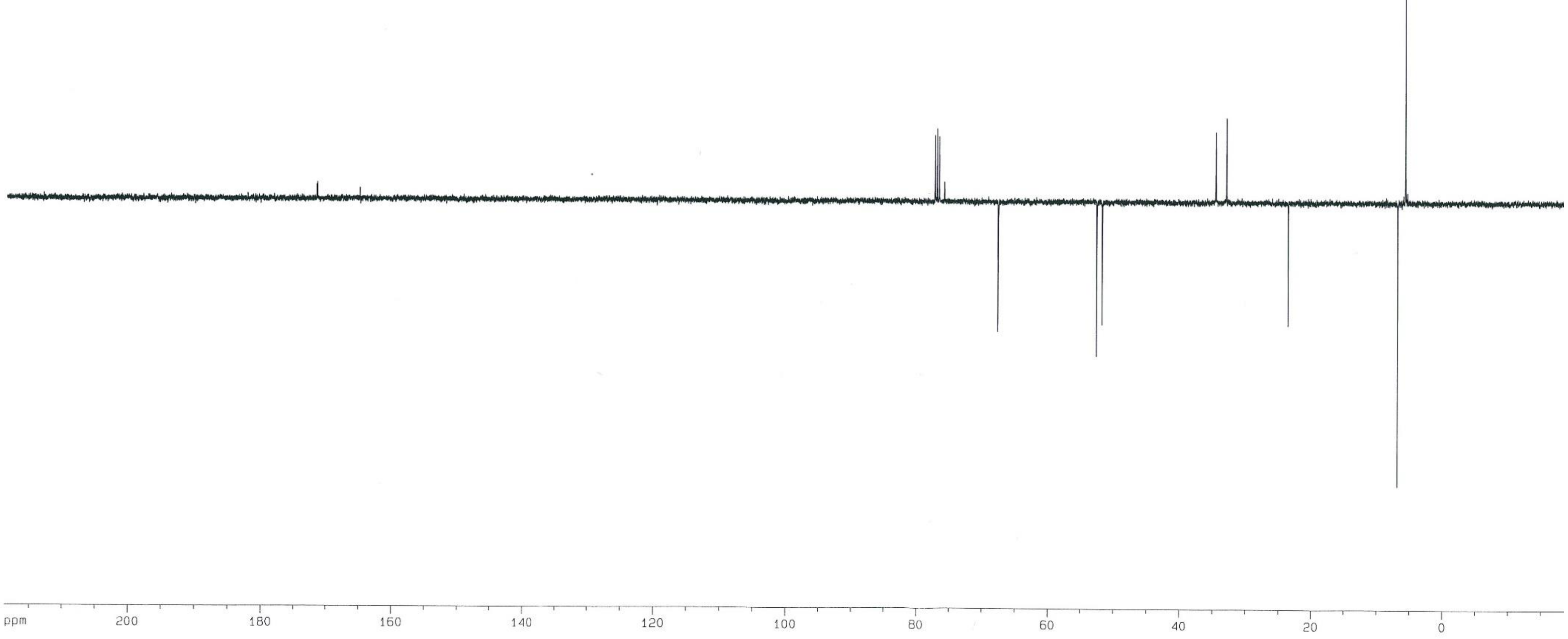

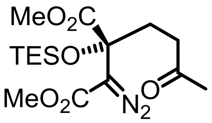

28

^{1}H (400 MHz, $CDCl_3$)

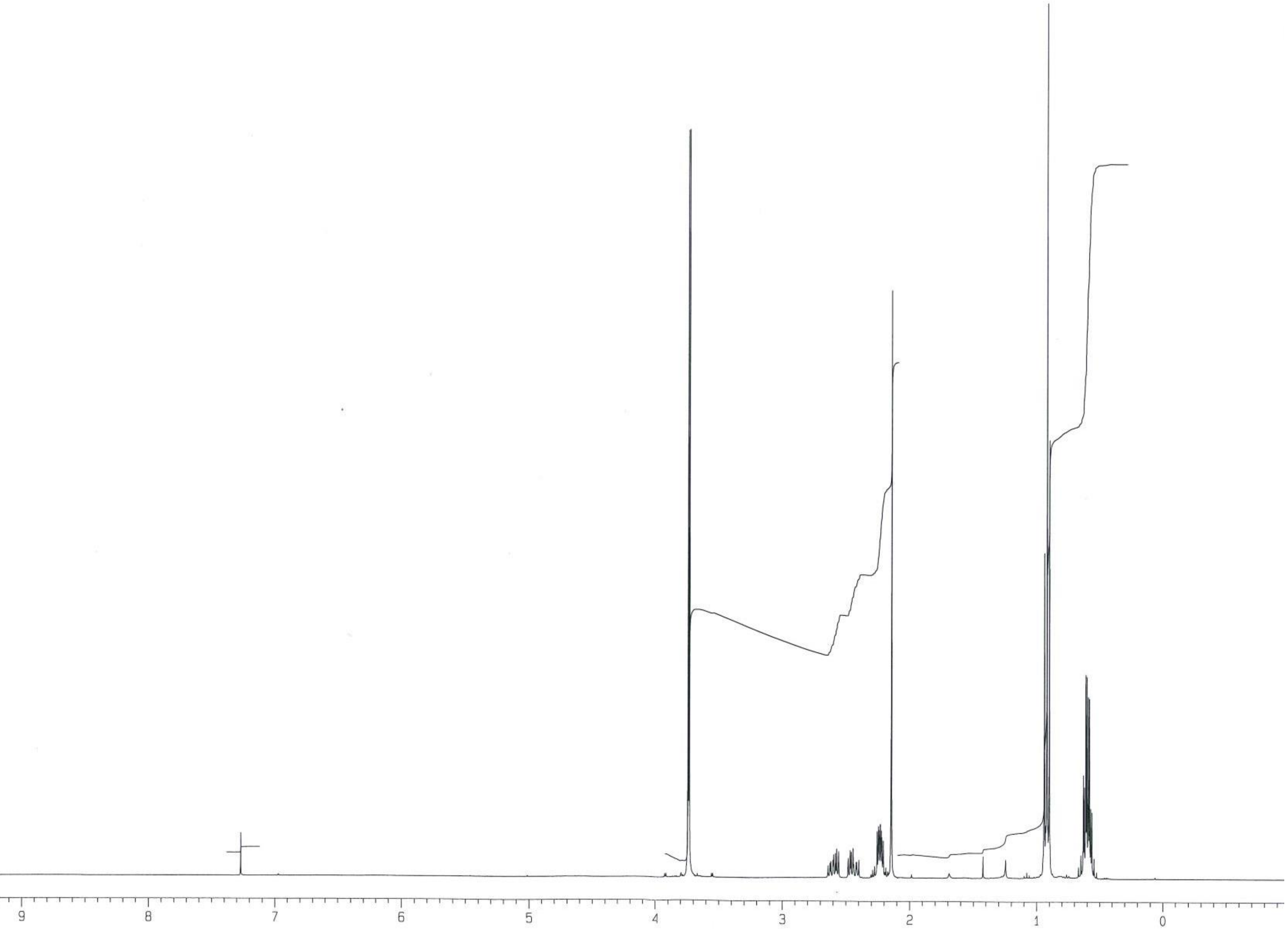

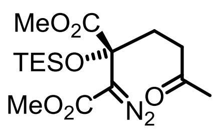

28

^{13}C (100 MHz, $CDCl_3$)

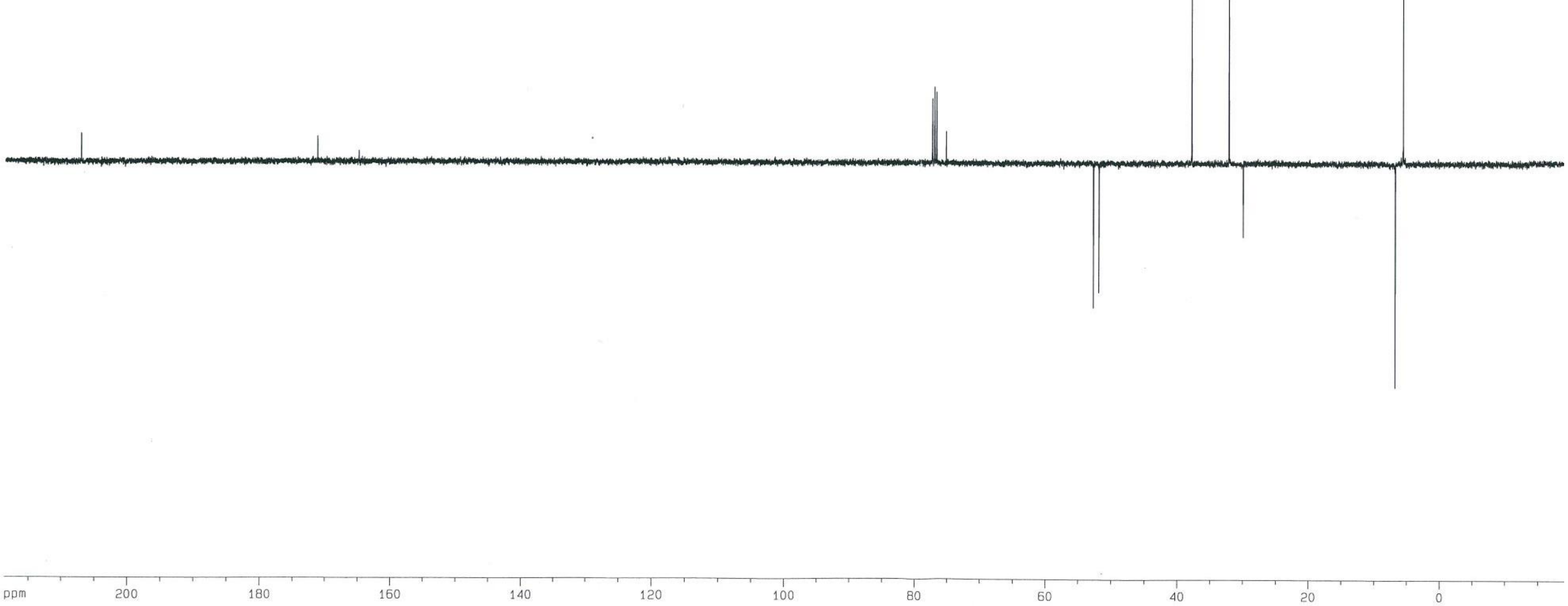

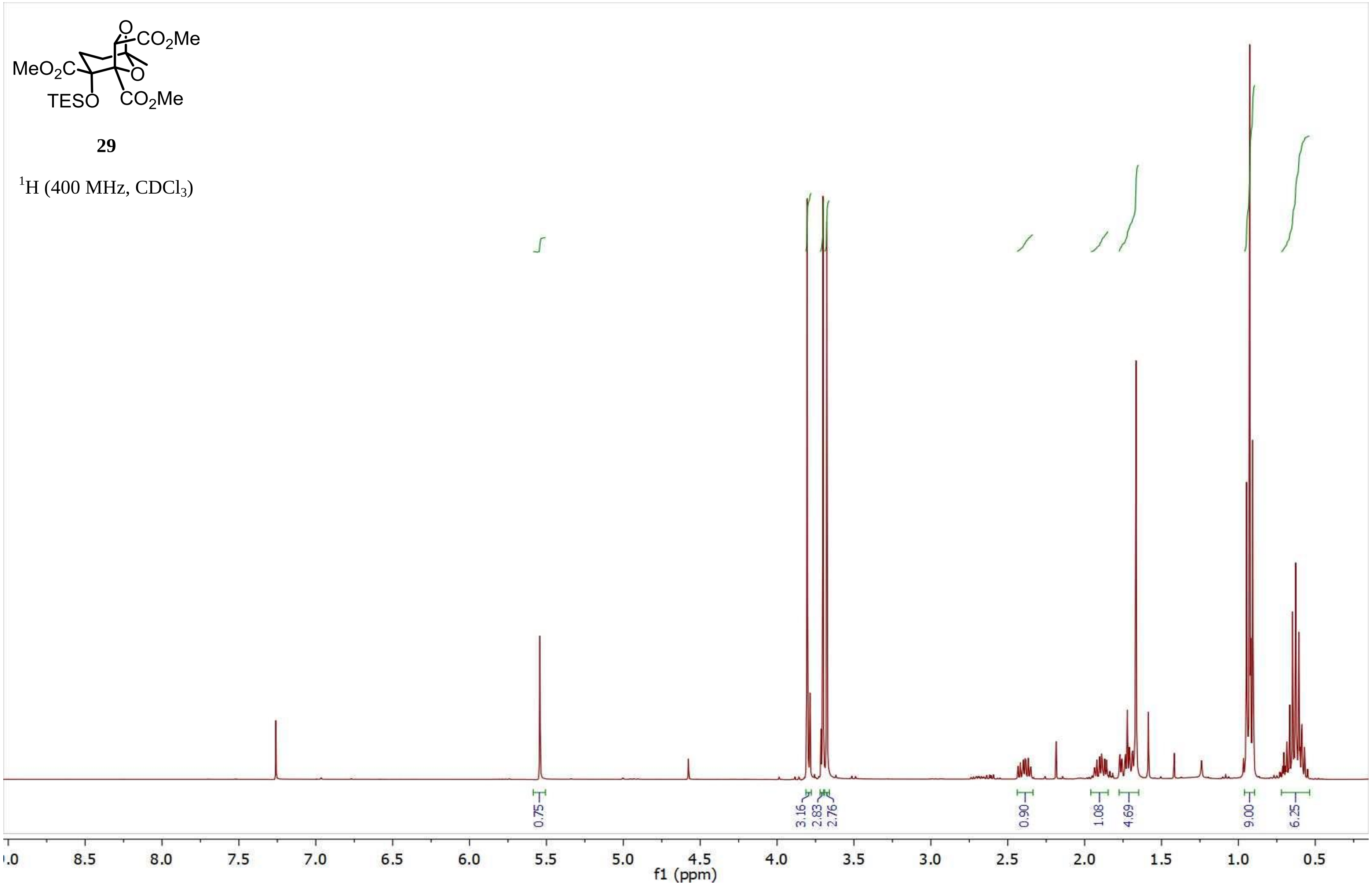

29

^{1}H (400 MHz, $CDCl_3$)

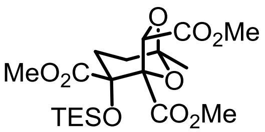

29

^{13}C (100 MHz, $CDCl_3$)

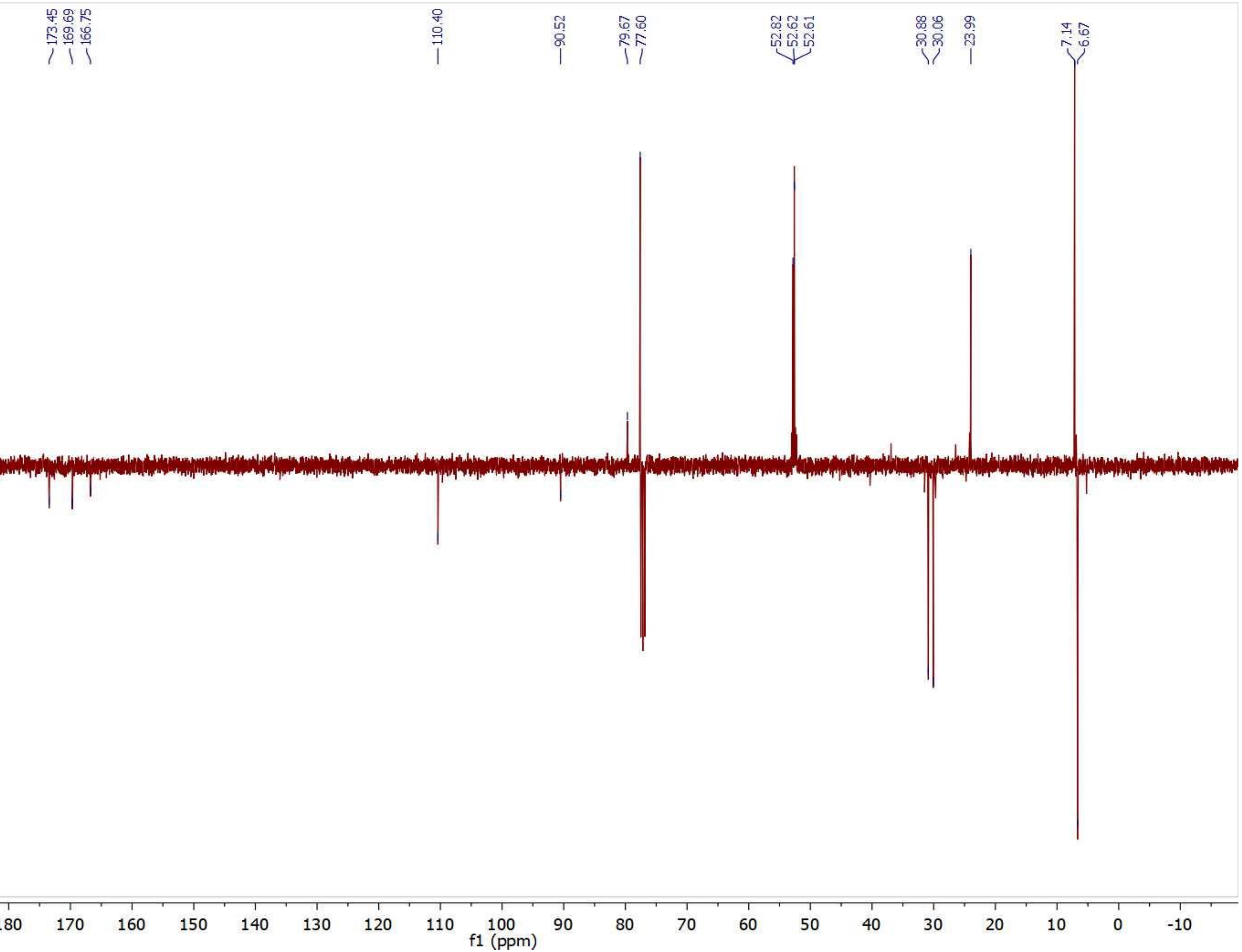

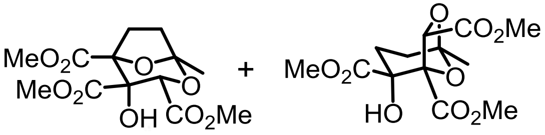

31 **30**

^{1}H (400 MHz, $CDCl_3$)

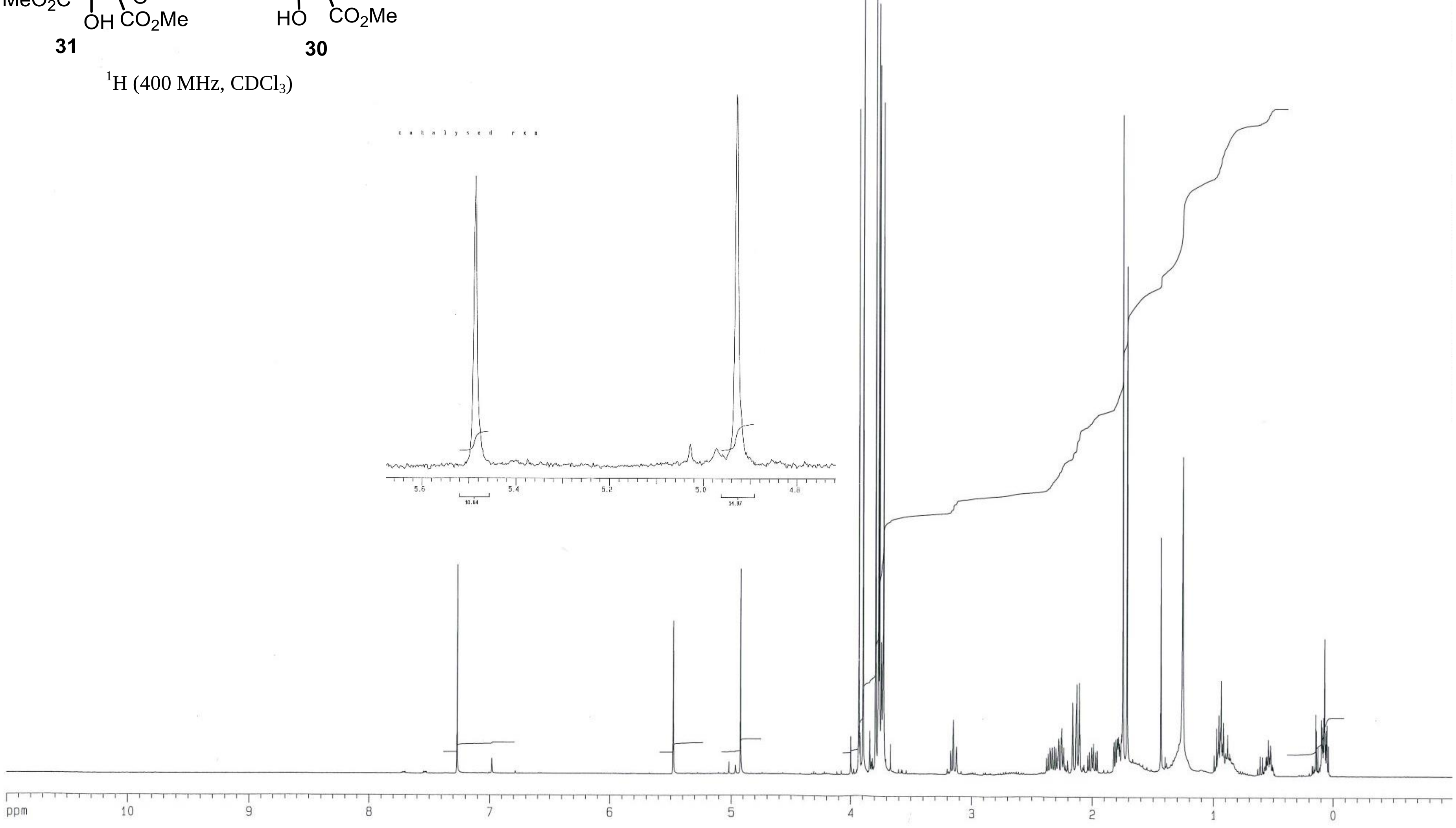

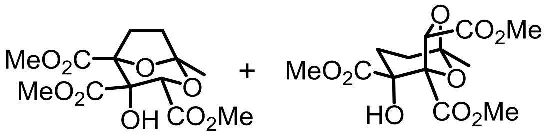

31 + **30**

^{13}C (100 MHz, $CDCl_3$)

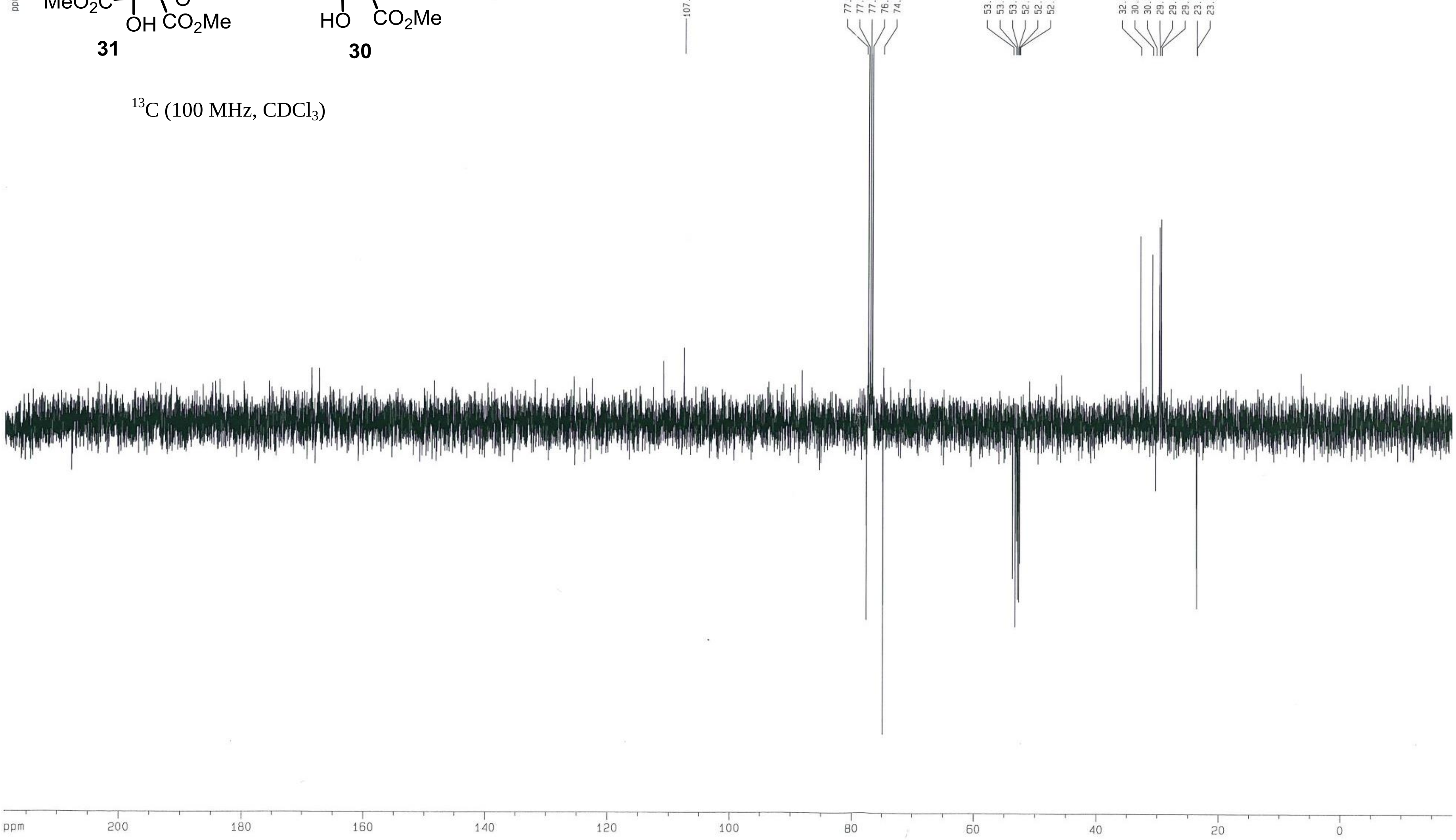

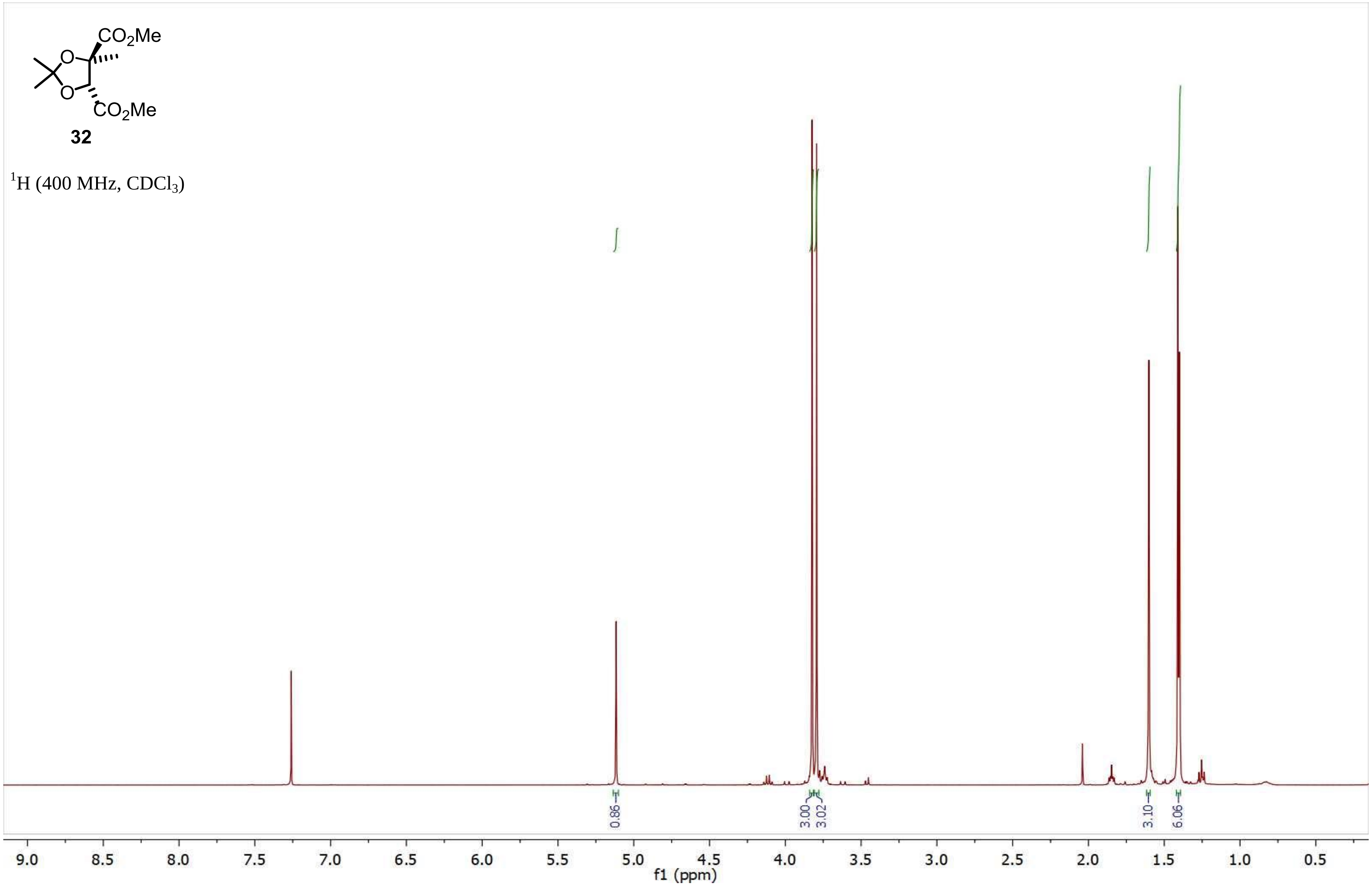

CO2Me
O
O
CO2Me
32
1H (400 MHz, CDCl3)
0.86
3.00
3.02
3.10
6.06
9.0 8.5 8.0 7.5 7.0 6.5 6.0 5.5 5.0 4.5 4.0 3.5 3.0 2.5 2.0 1.5 1.0 0.5
f1 (ppm)

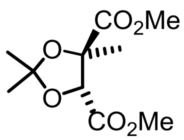

32

^{13}C (100 MHz, $CDCl_3$)

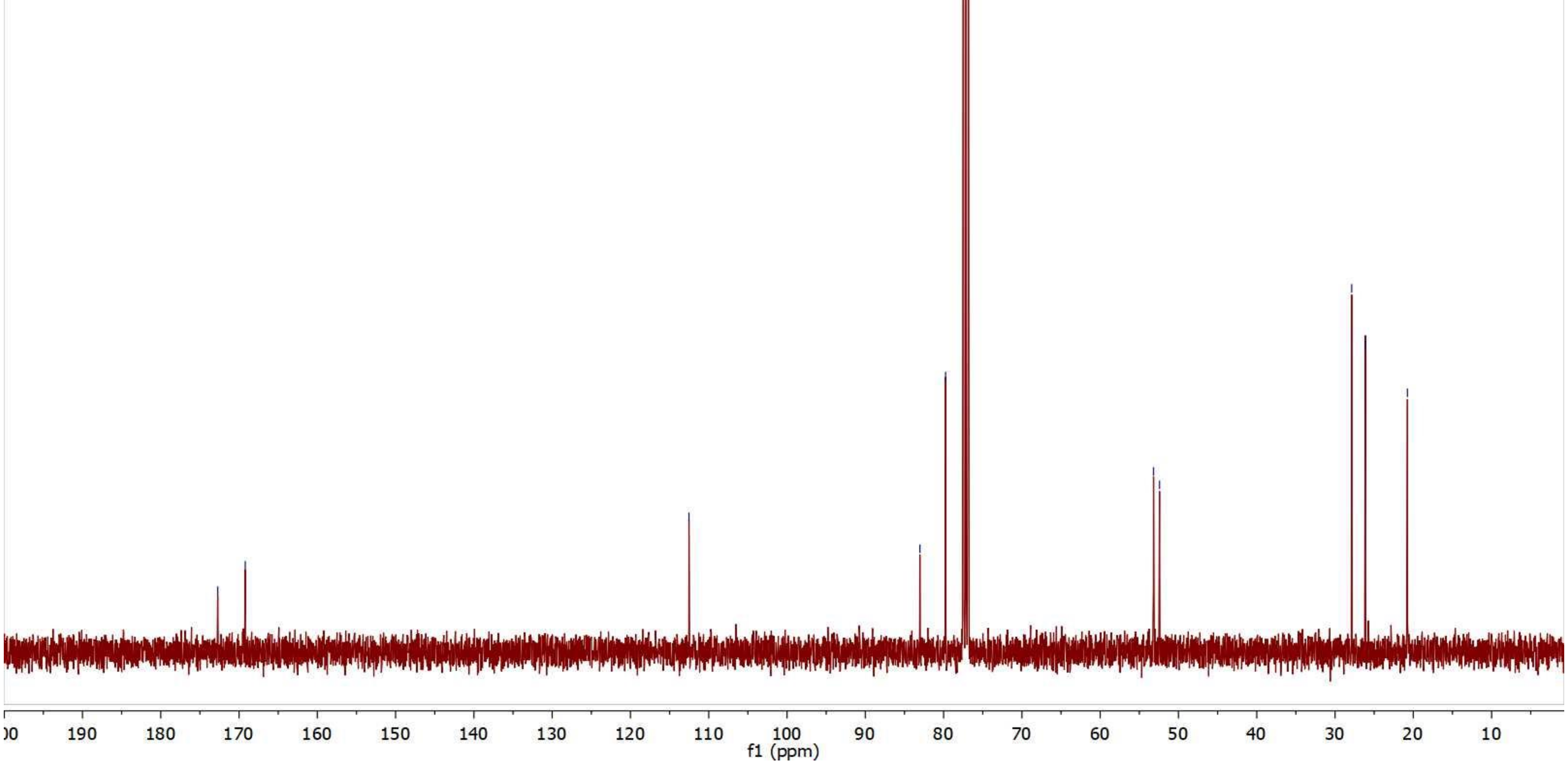

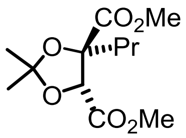

33a

^{1}H (500 MHz, $CDCl_3$)

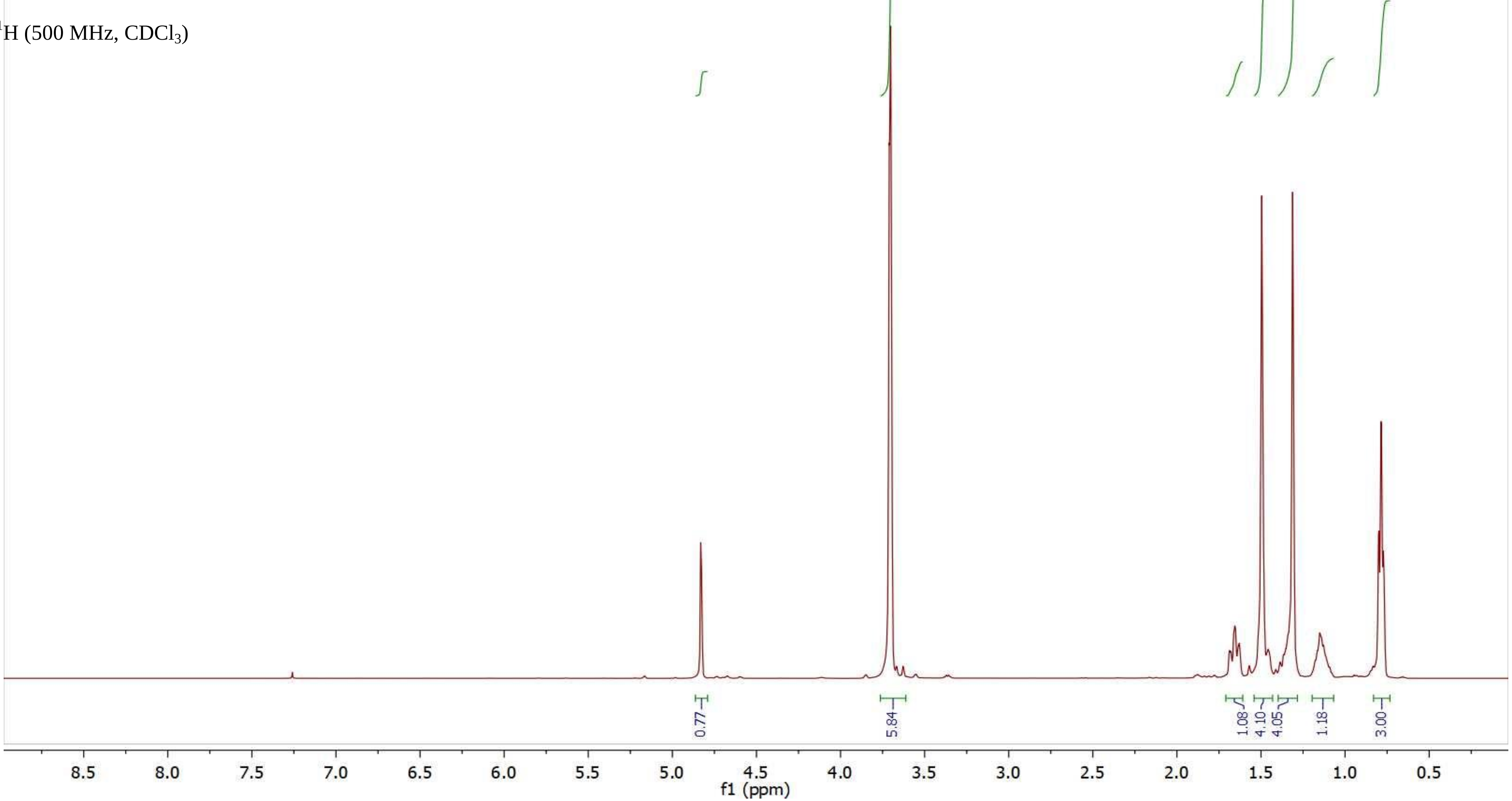

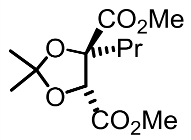

33a

^{13}C (125 MHz, $CDCl_3$)

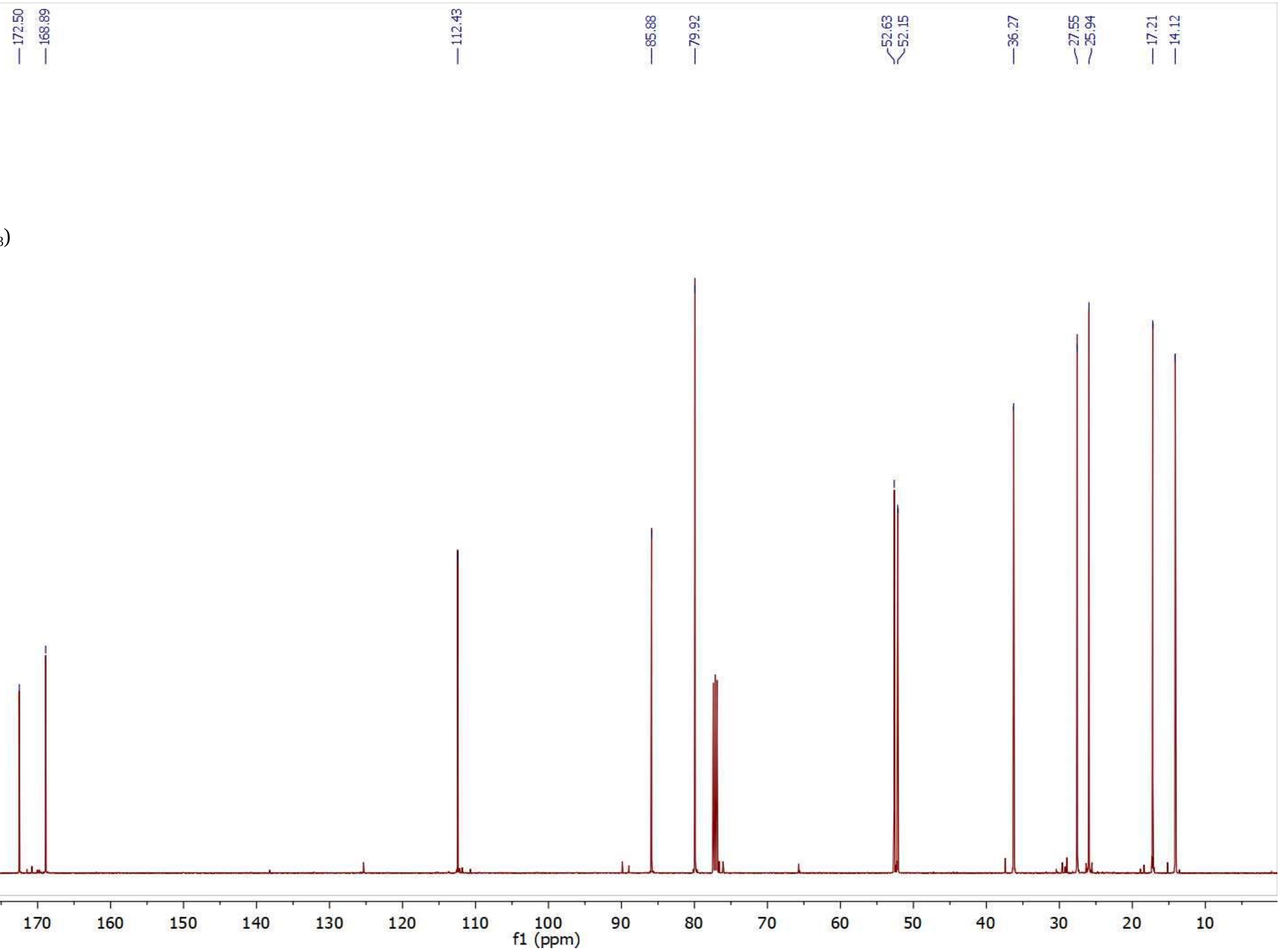

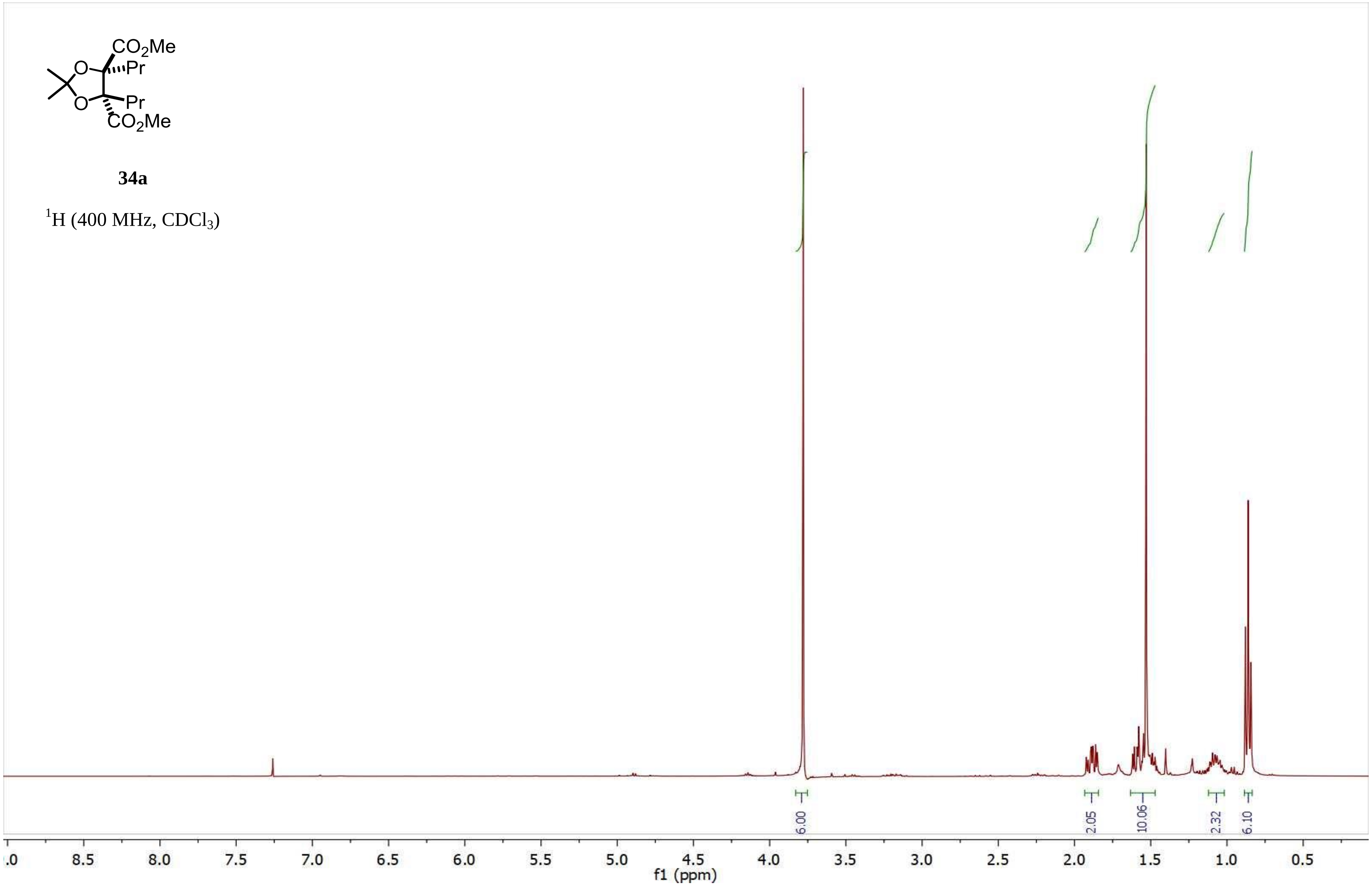

CO_2Me
O
Pr
O
Pr
CO_2Me
34a
^1H (400 MHz, $CDCl_3$)
6.00
2.05
10.06
2.32
6.10
.0
8.5
8.0
7.5
7.0
6.5
6.0
5.5
5.0
4.5
4.0
3.5
3.0
2.5
2.0
1.5
1.0
0.5
f1 (ppm)

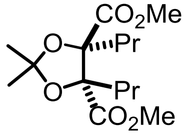

34a

^{13}C (100 MHz, $CDCl_3$)

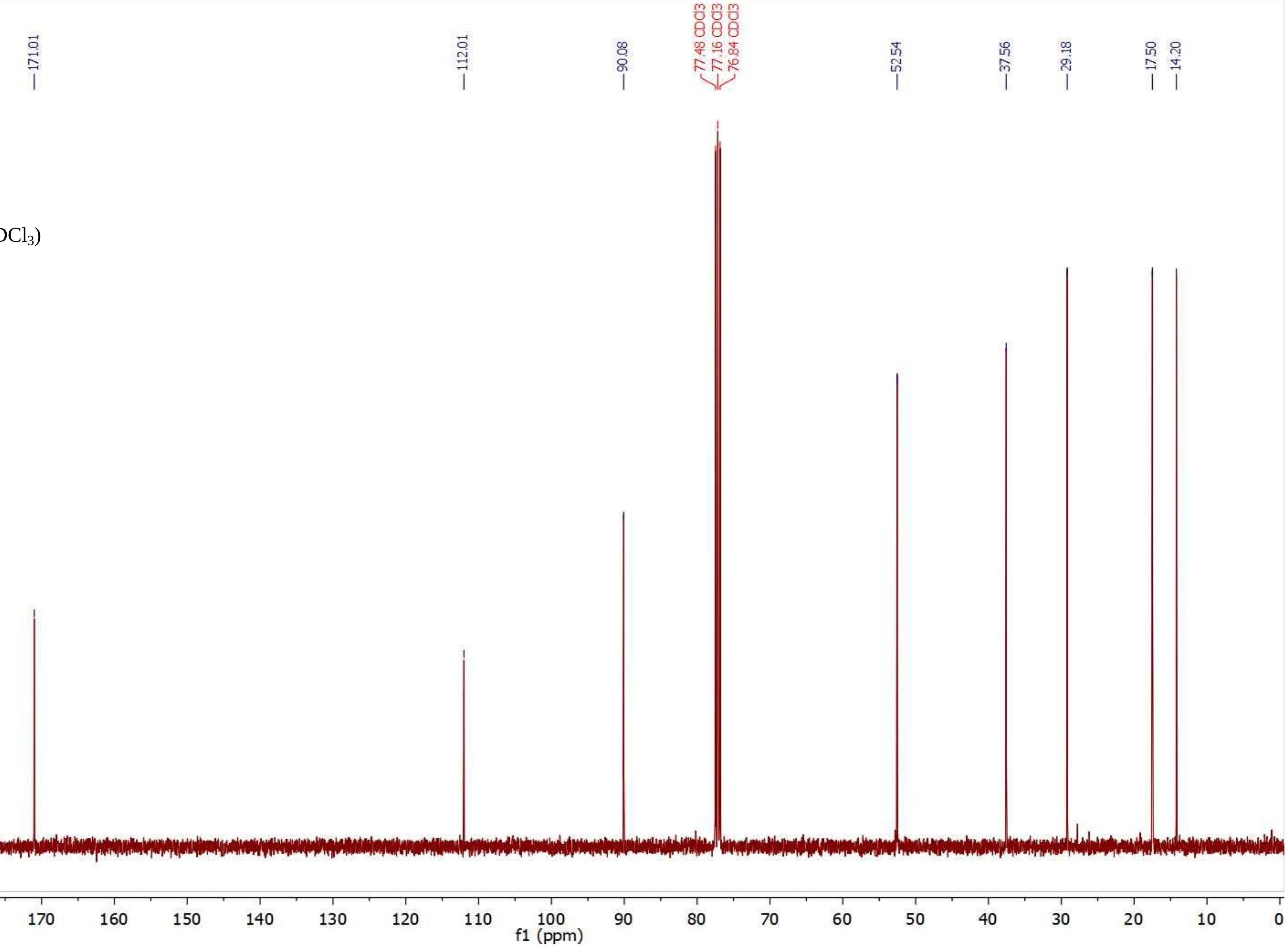

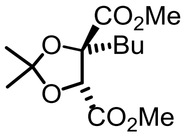

33b

^{1}H (400 MHz, $CDCl_3$)

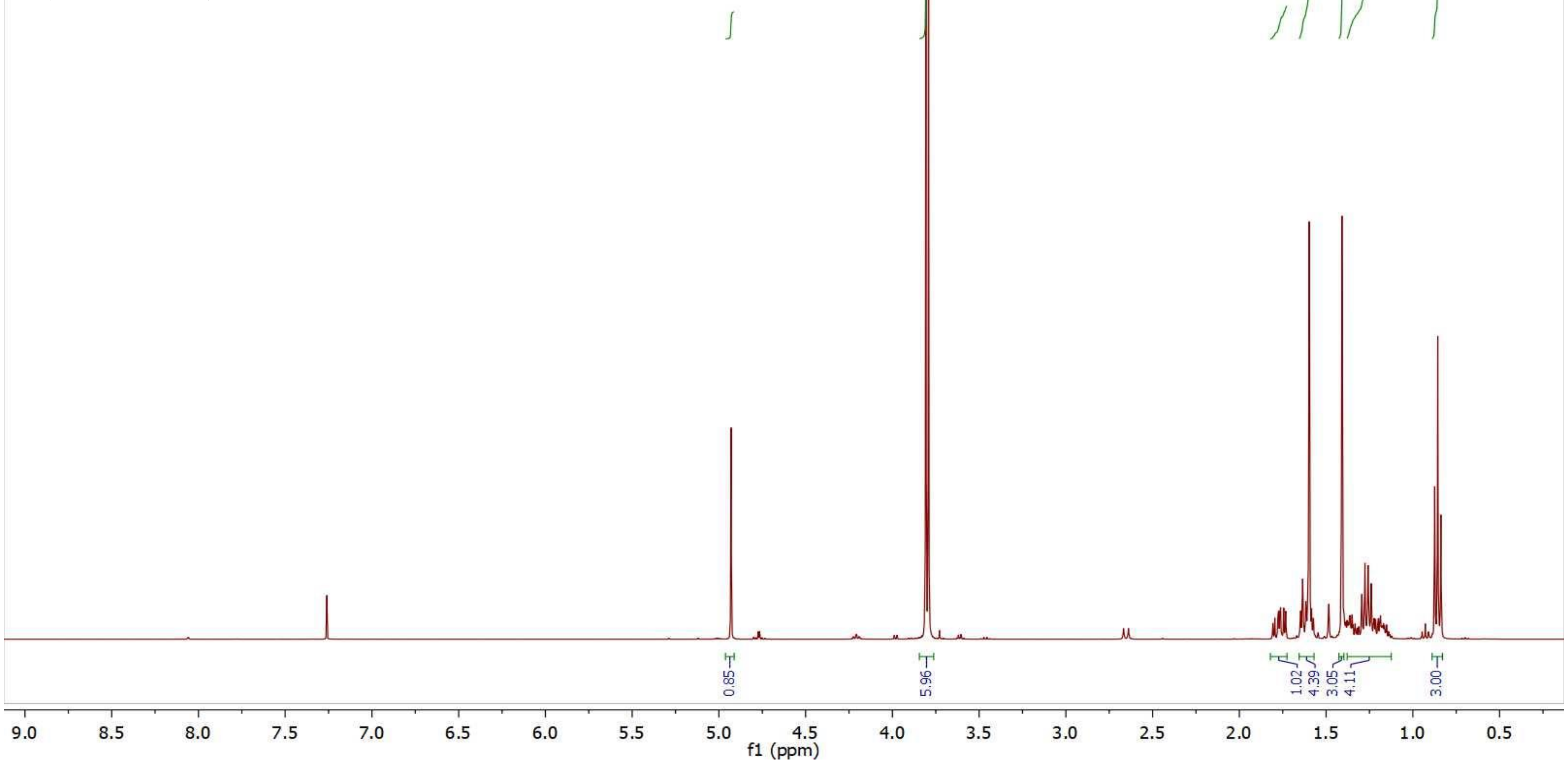

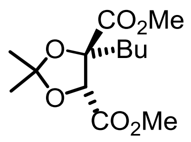

33b

^{13}C (100 MHz, $CDCl_3$)

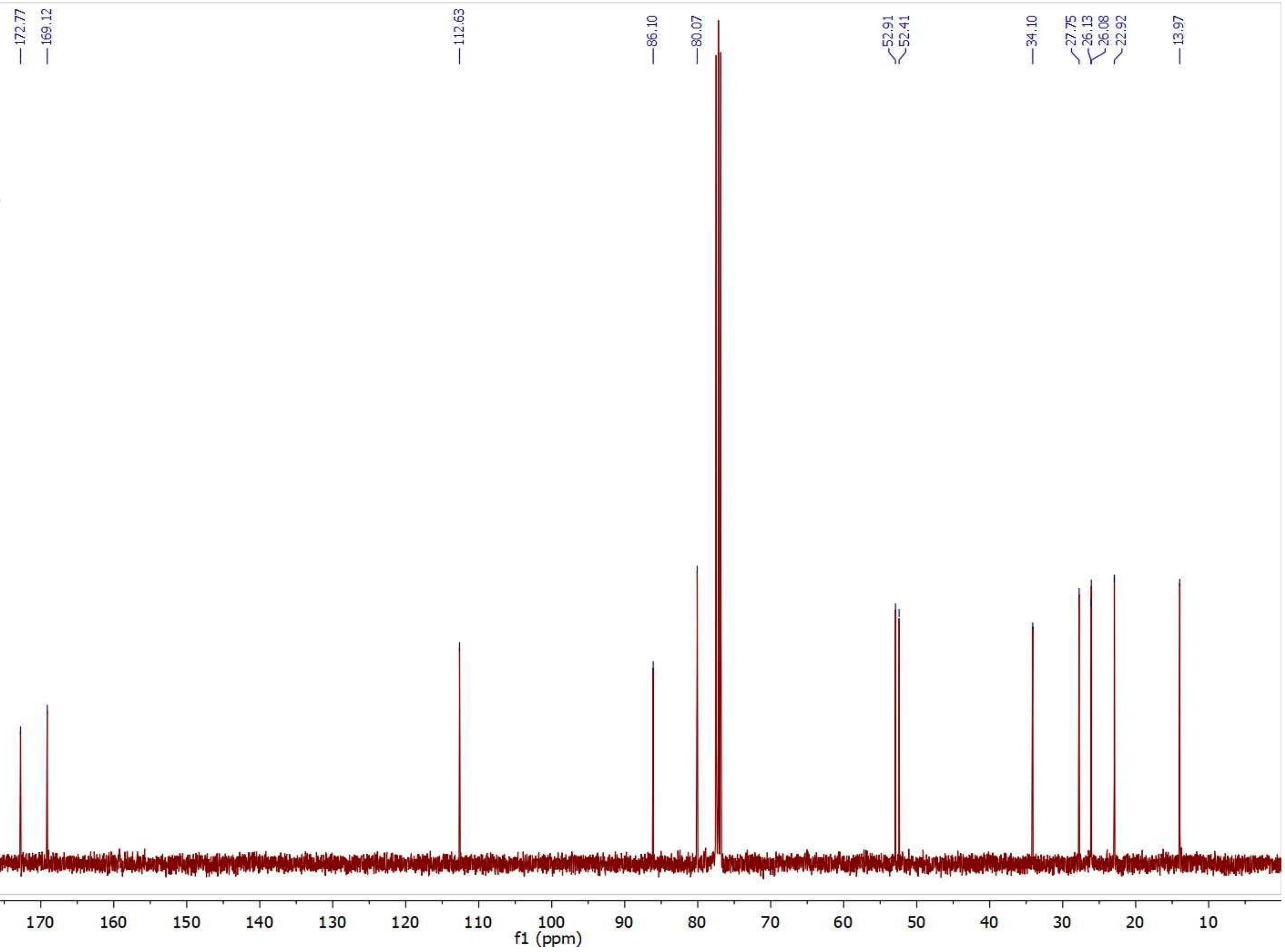

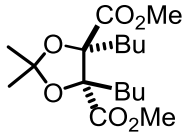

34b

^{1}H (400 MHz, $CDCl_3$)

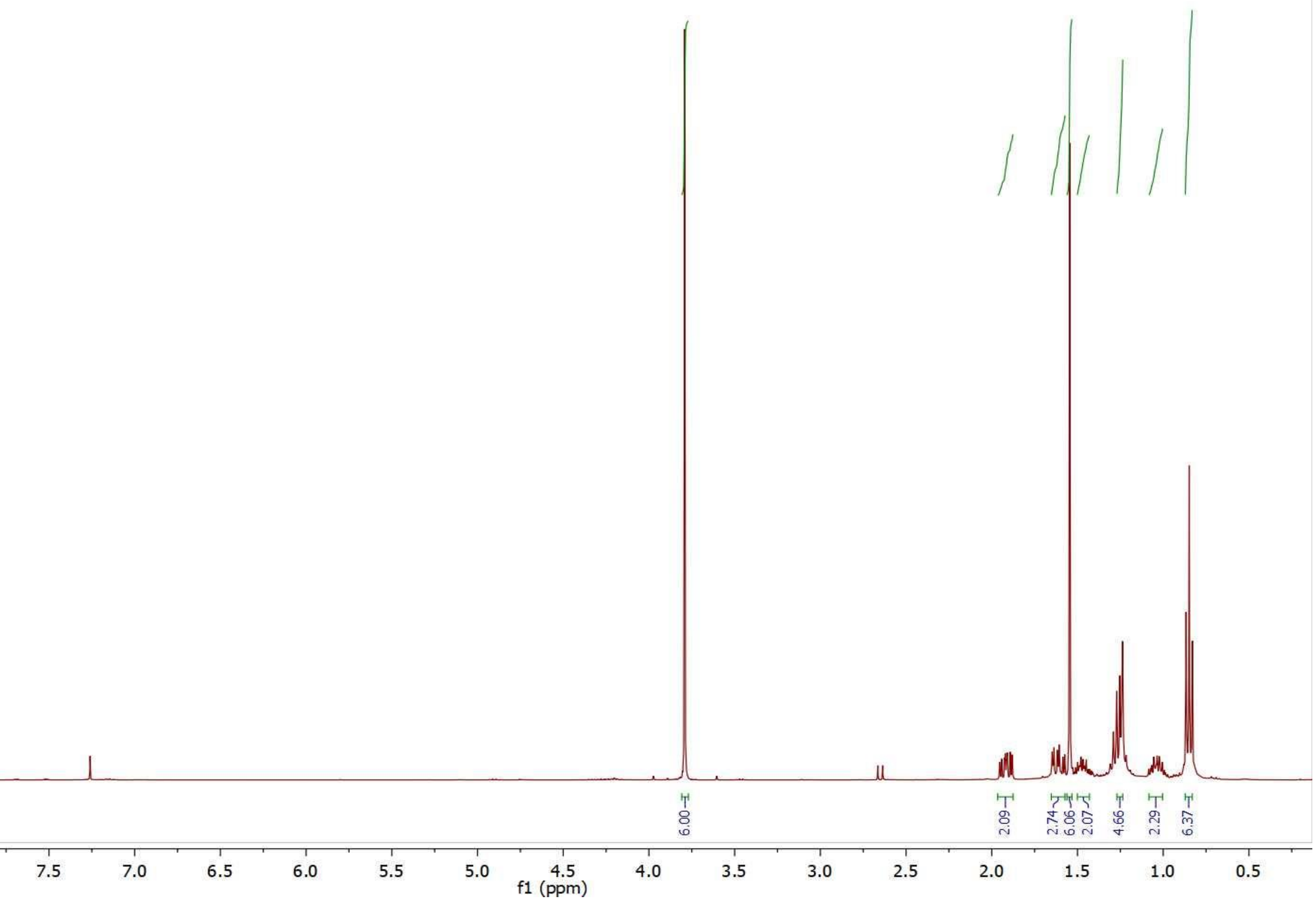

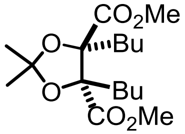

34b

^{13}C (100 MHz, $CDCl_3$)

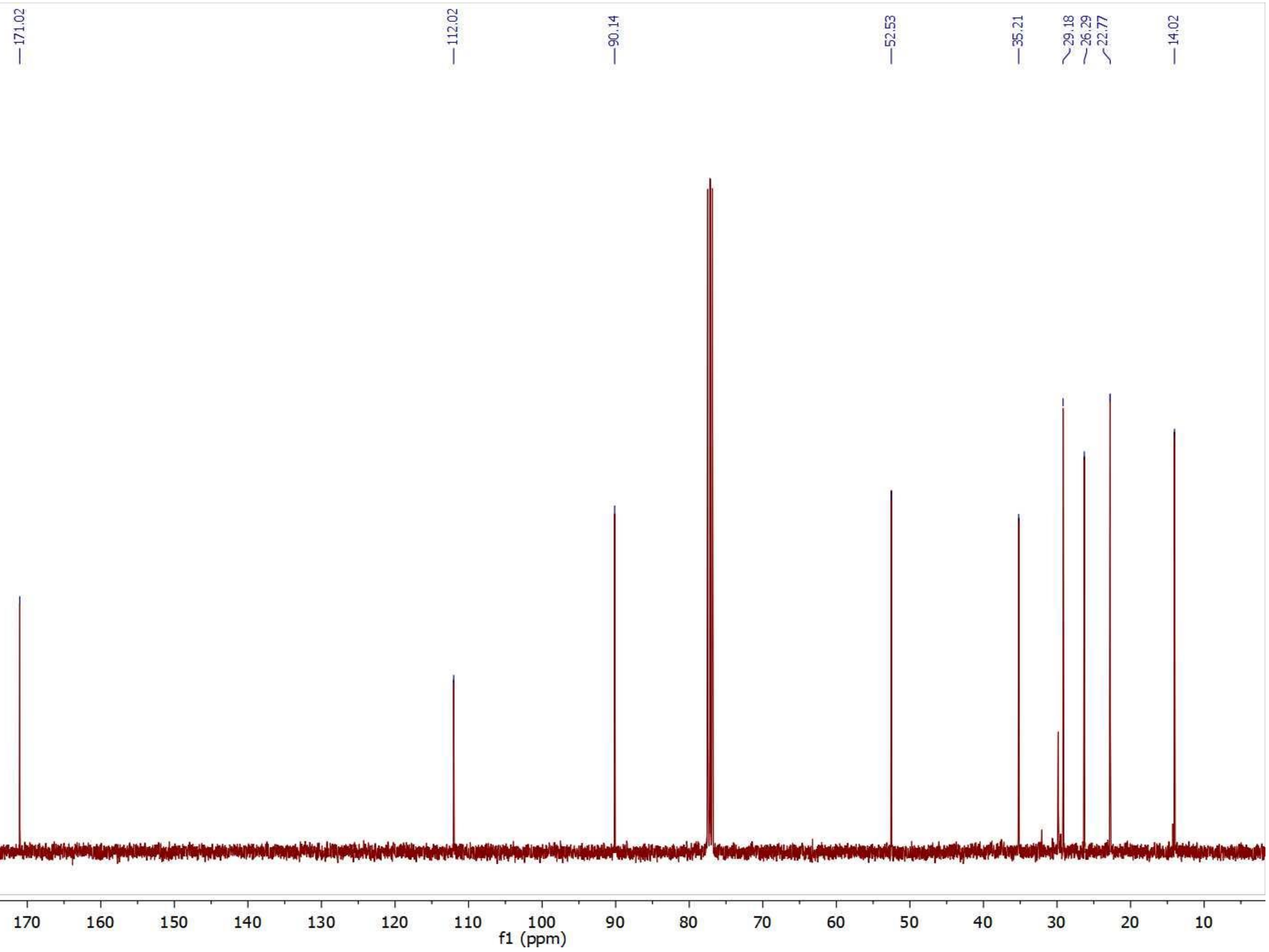

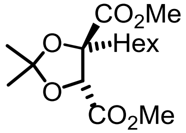

33c

^{1}H (400 MHz, $CDCl_3$)

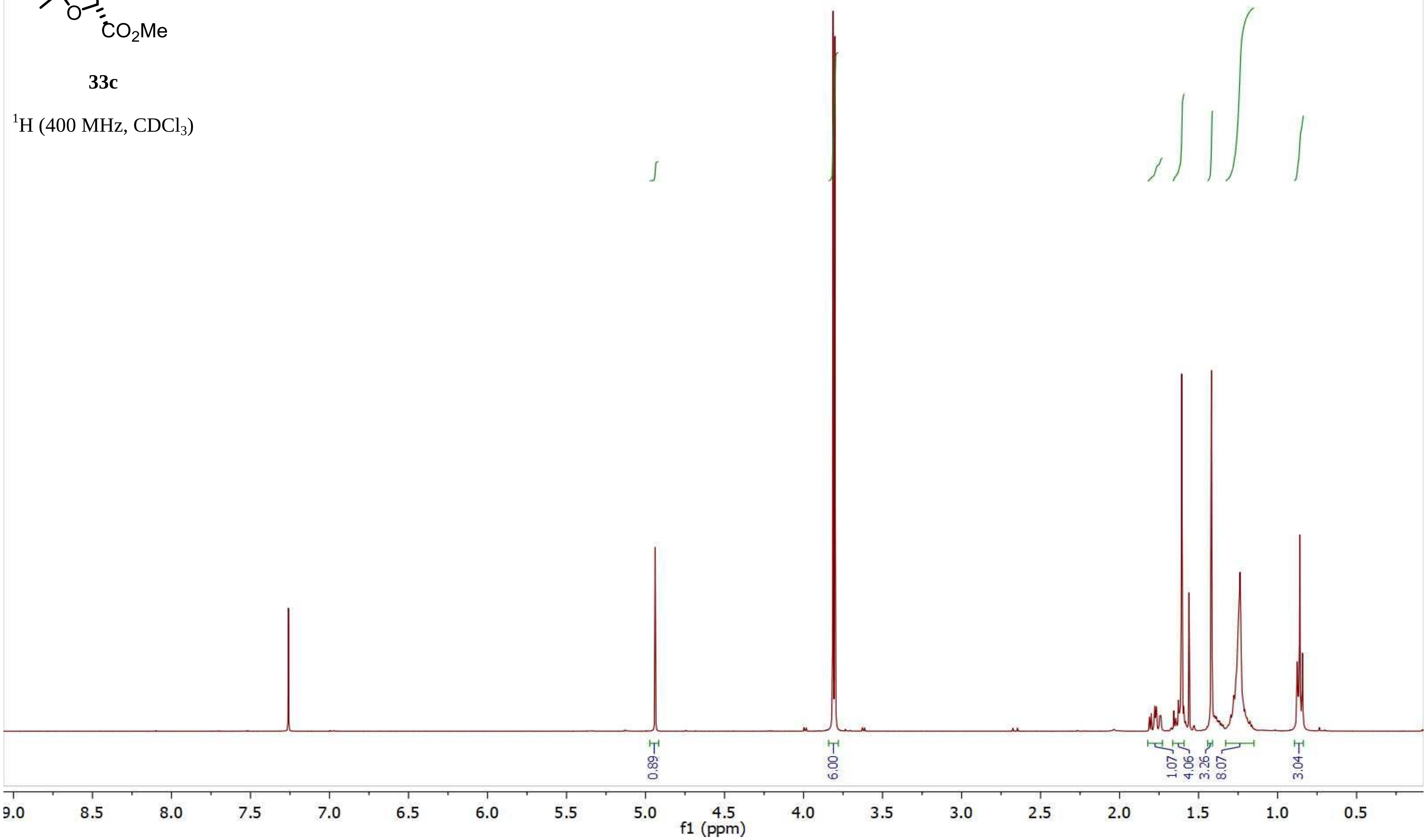

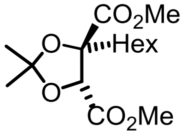

33c

^{13}C (100 MHz, $CDCl_3$)

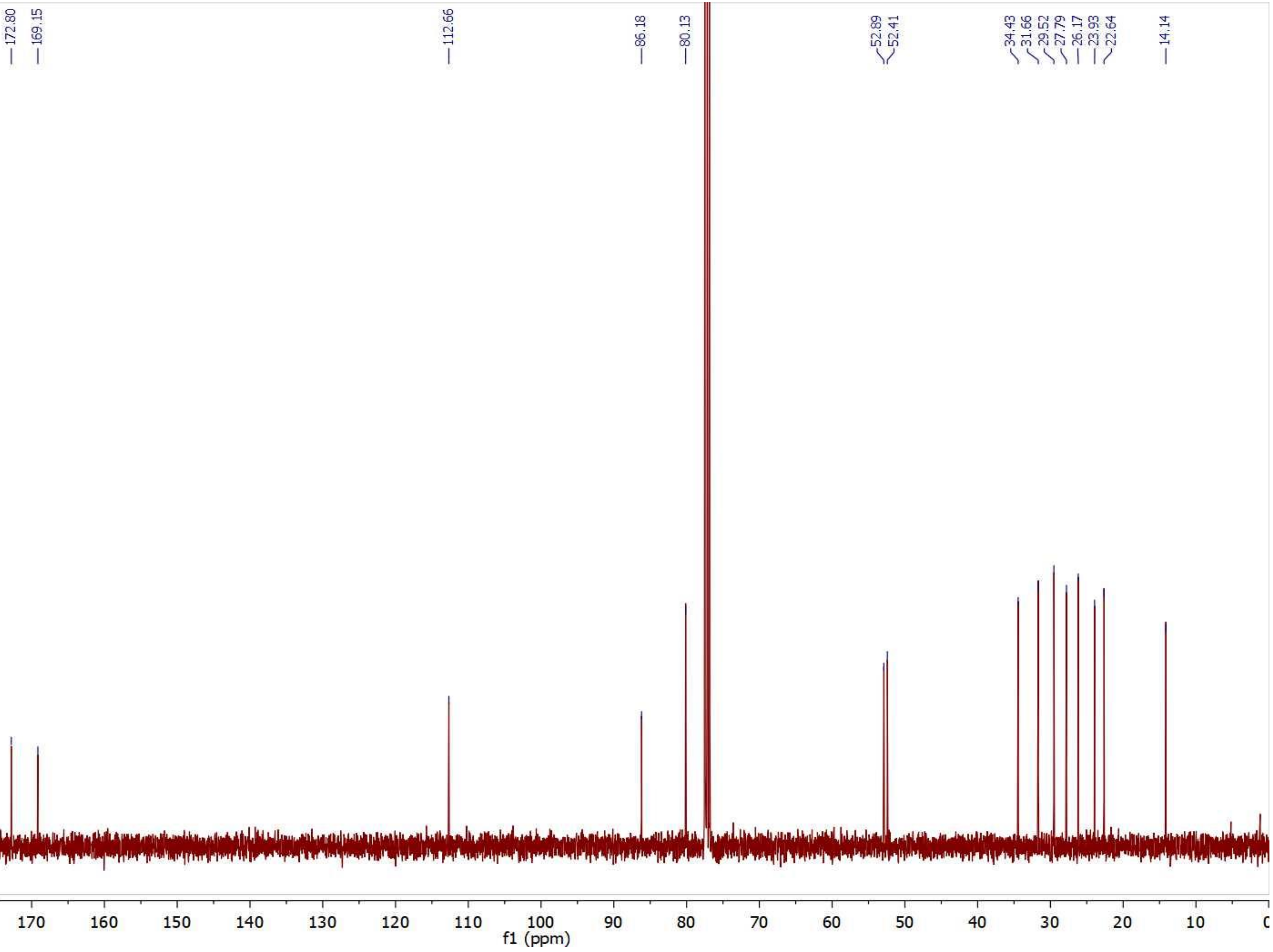

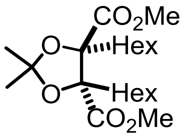

34c

^{1}H (400 MHz, $CDCl_3$)

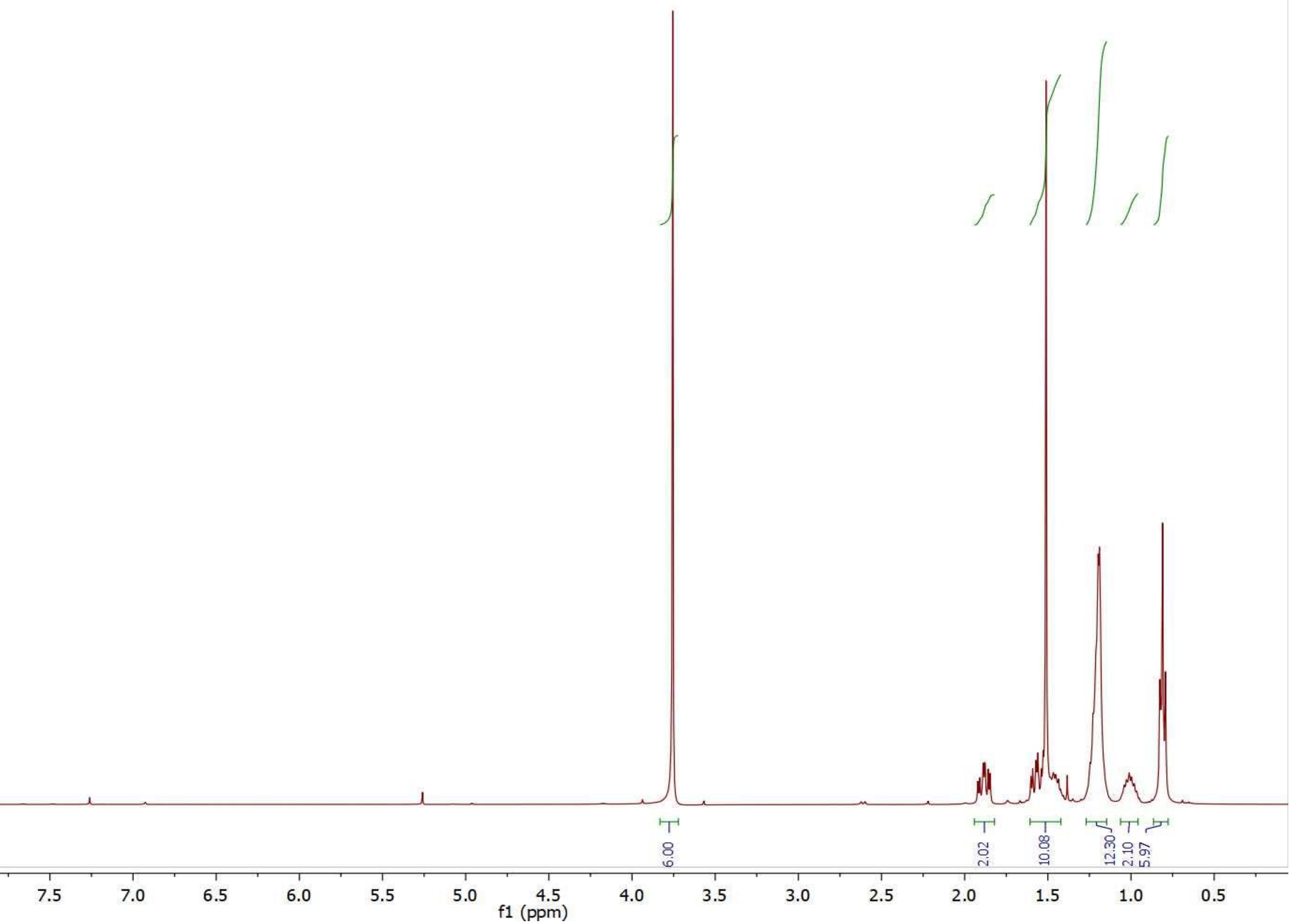

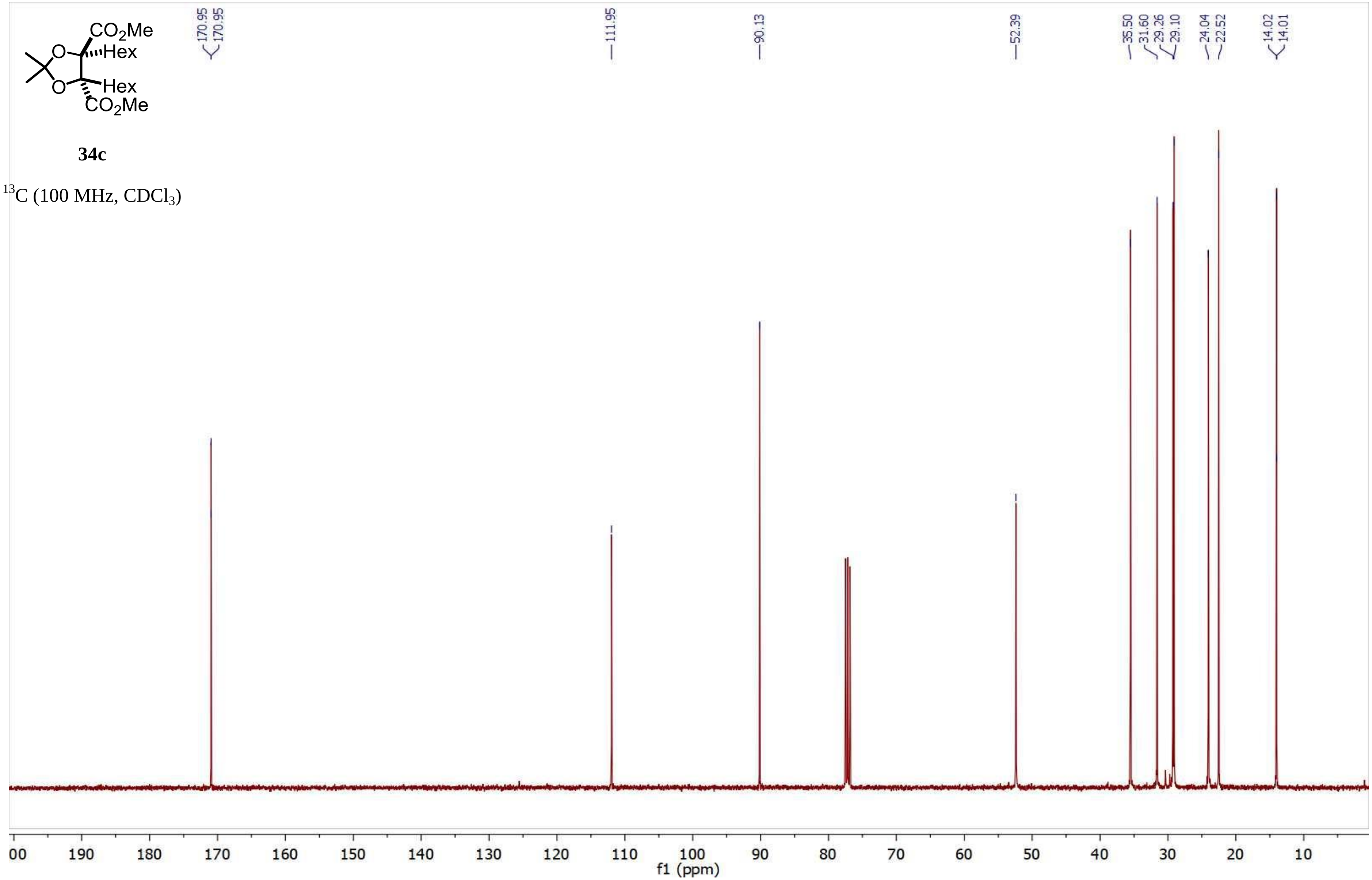

34c

^{13}C (100 MHz, $CDCl_3$)

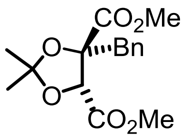

33d

^{1}H (500 MHz, $CDCl_3$)

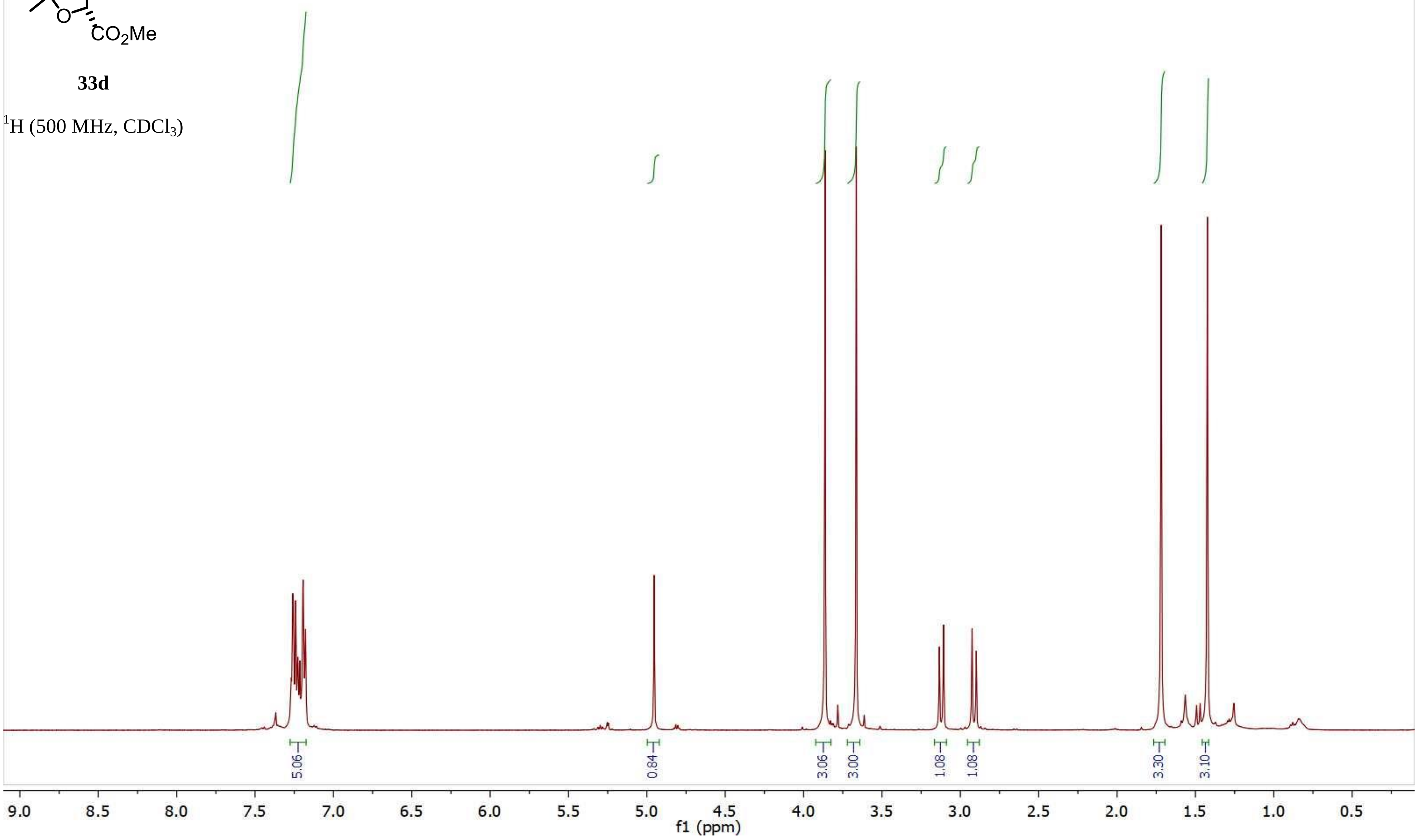

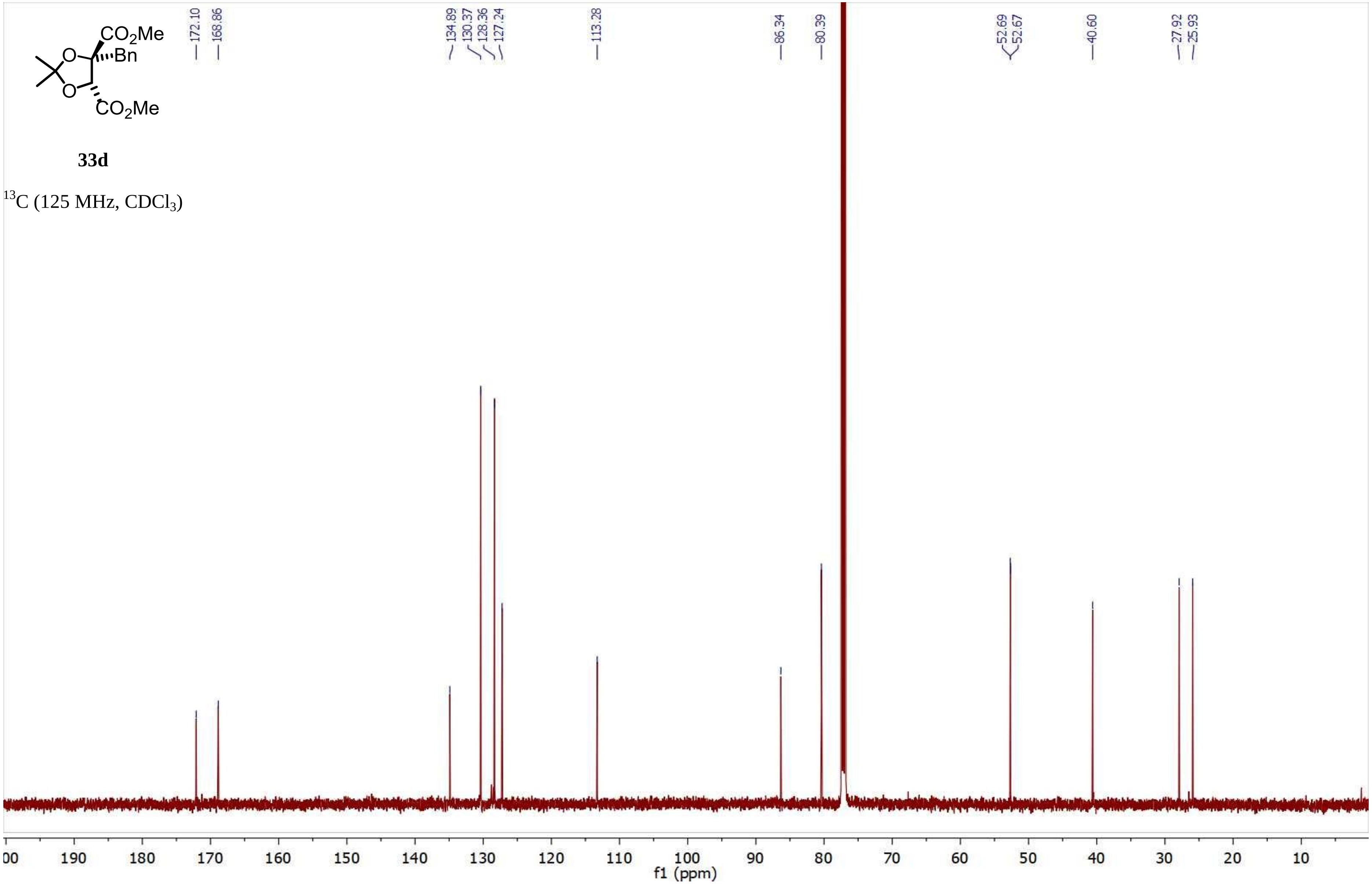

172.10
168.86
134.89
130.37
128.36
127.24
113.28
86.34
80.39
52.69
52.67
40.60
27.92
25.93
CO2Me
Bn
O
O
CO2Me
33d
^{13}C (125 MHz, $CDCl_3$)
00
190
180
170
160
150
140
130
120
110
100
90
80
70
60
50
40
30
20
10
f1 (ppm)

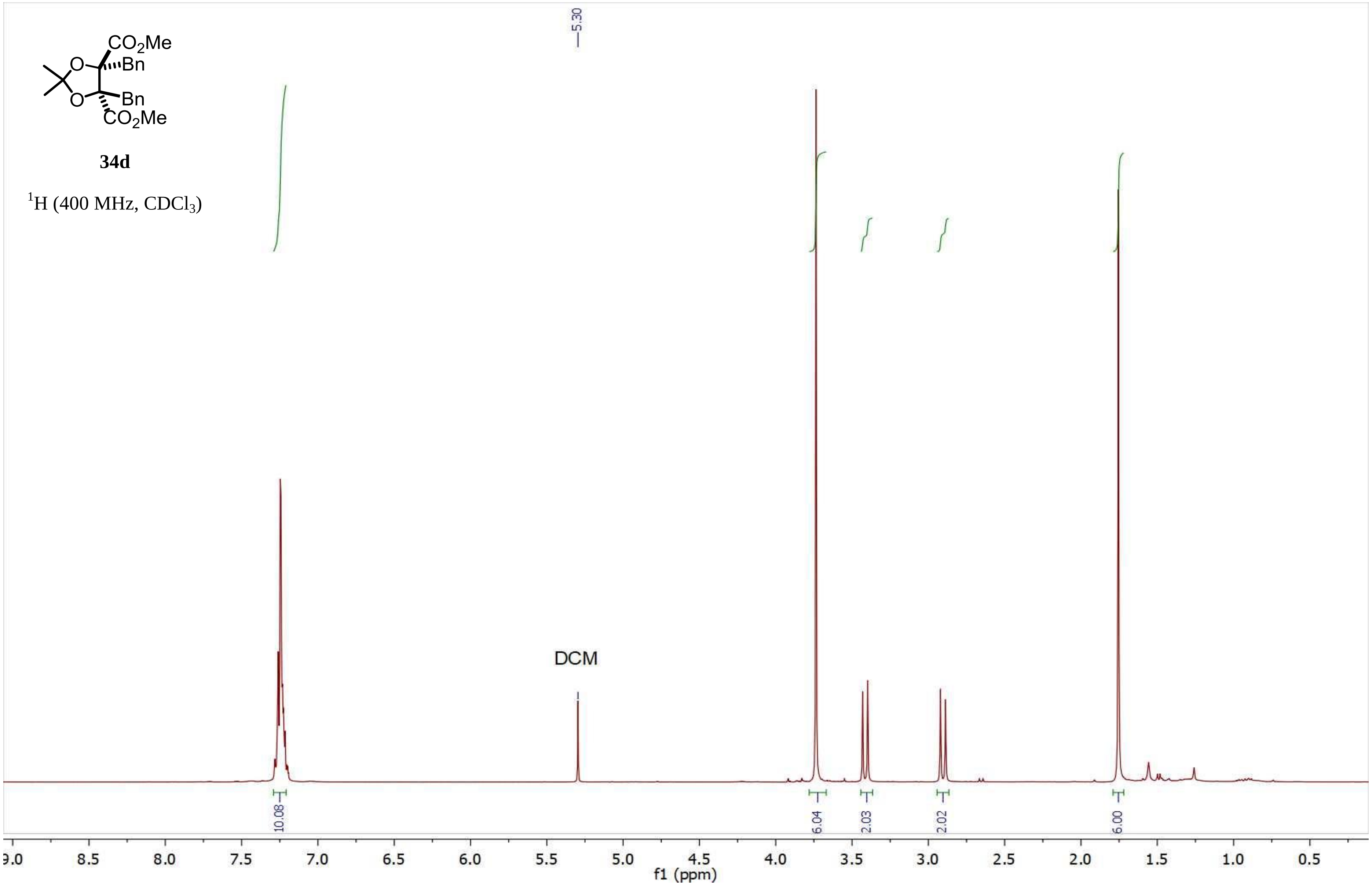
CO2Me
Bn
O
O
Bn
CO2Me
34d
1H (400 MHz, CDCl3)
5.30
DCM
10.08
6.04
2.03
2.02
6.00
9.0 8.5 8.0 7.5 7.0 6.5 6.0 5.5 5.0 4.5 4.0 3.5 3.0 2.5 2.0 1.5 1.0 0.5
f1 (ppm)

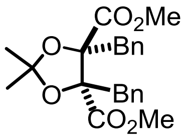

34d

^{13}C (100 MHz, $CDCl_3$)

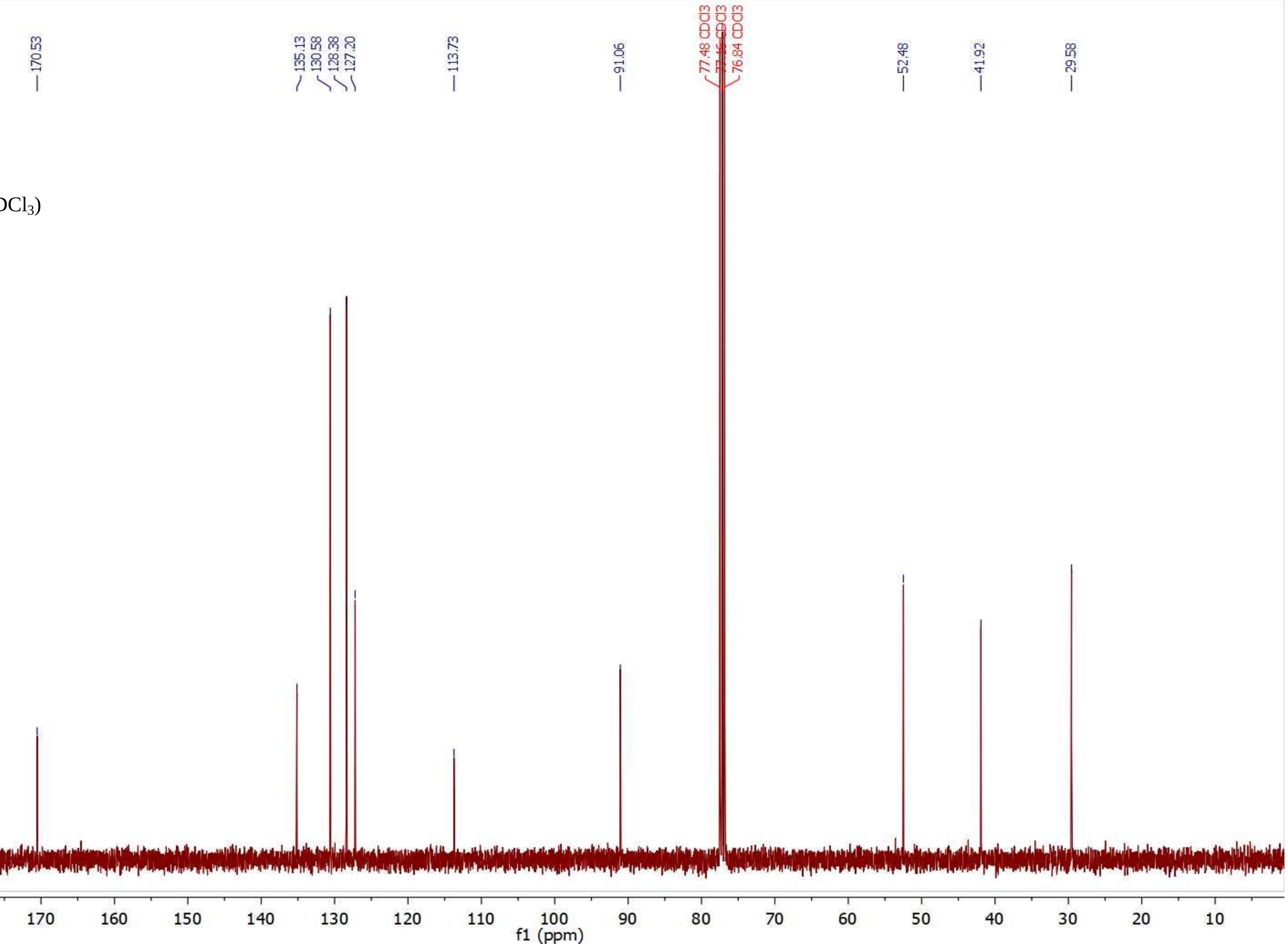

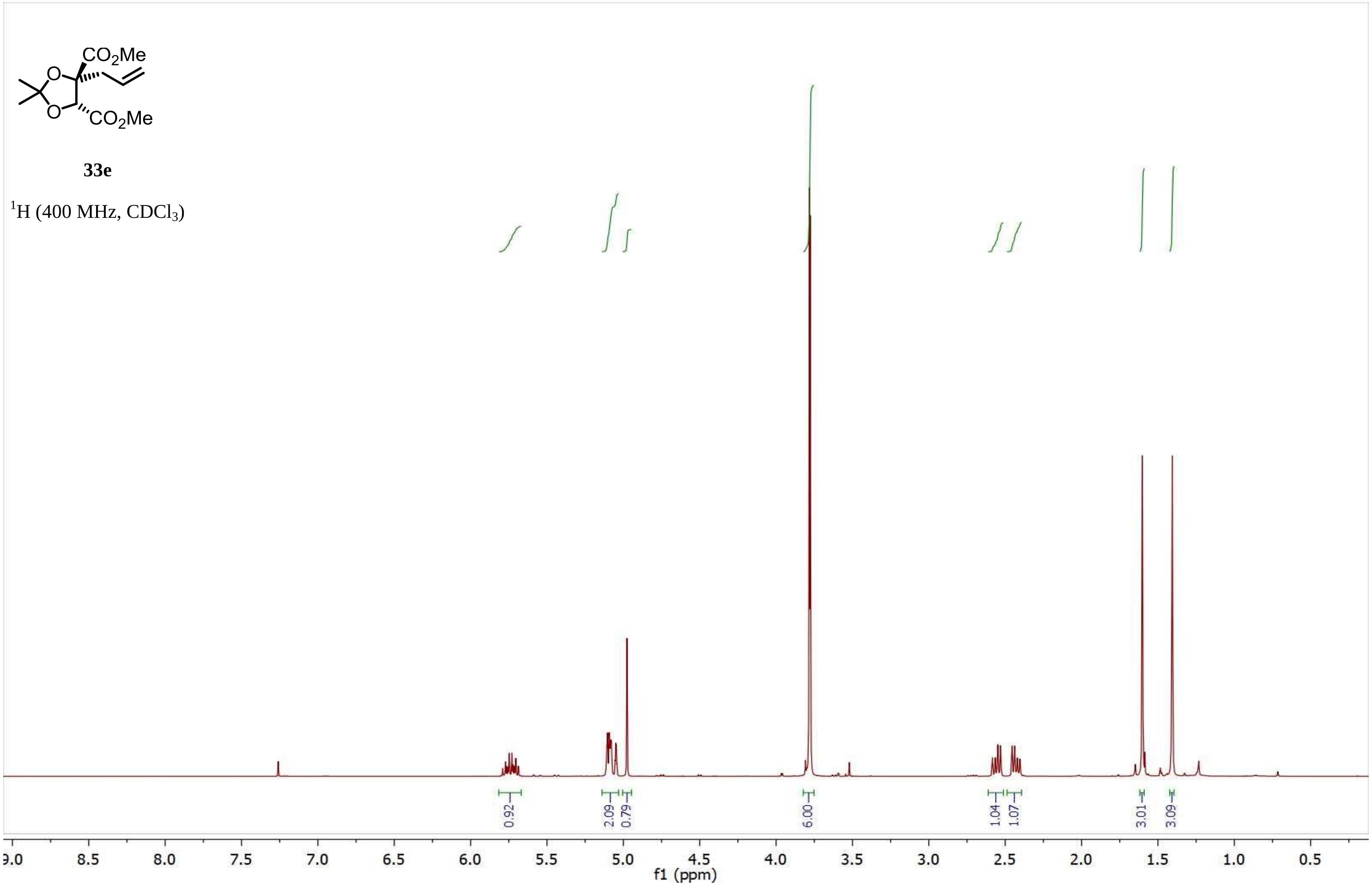
CO2Me
O
O
CO2Me
33e
1H (400 MHz, CDCl3)
0.92
2.09
0.79
6.00
1.04
1.07
3.01
3.09
9.0
8.5
8.0
7.5
7.0
6.5
6.0
5.5
5.0
4.5
4.0
3.5
3.0
2.5
2.0
1.5
1.0
0.5
f1 (ppm)

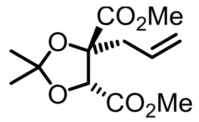

33e

^{13}C (100 MHz, $CDCl_3$)

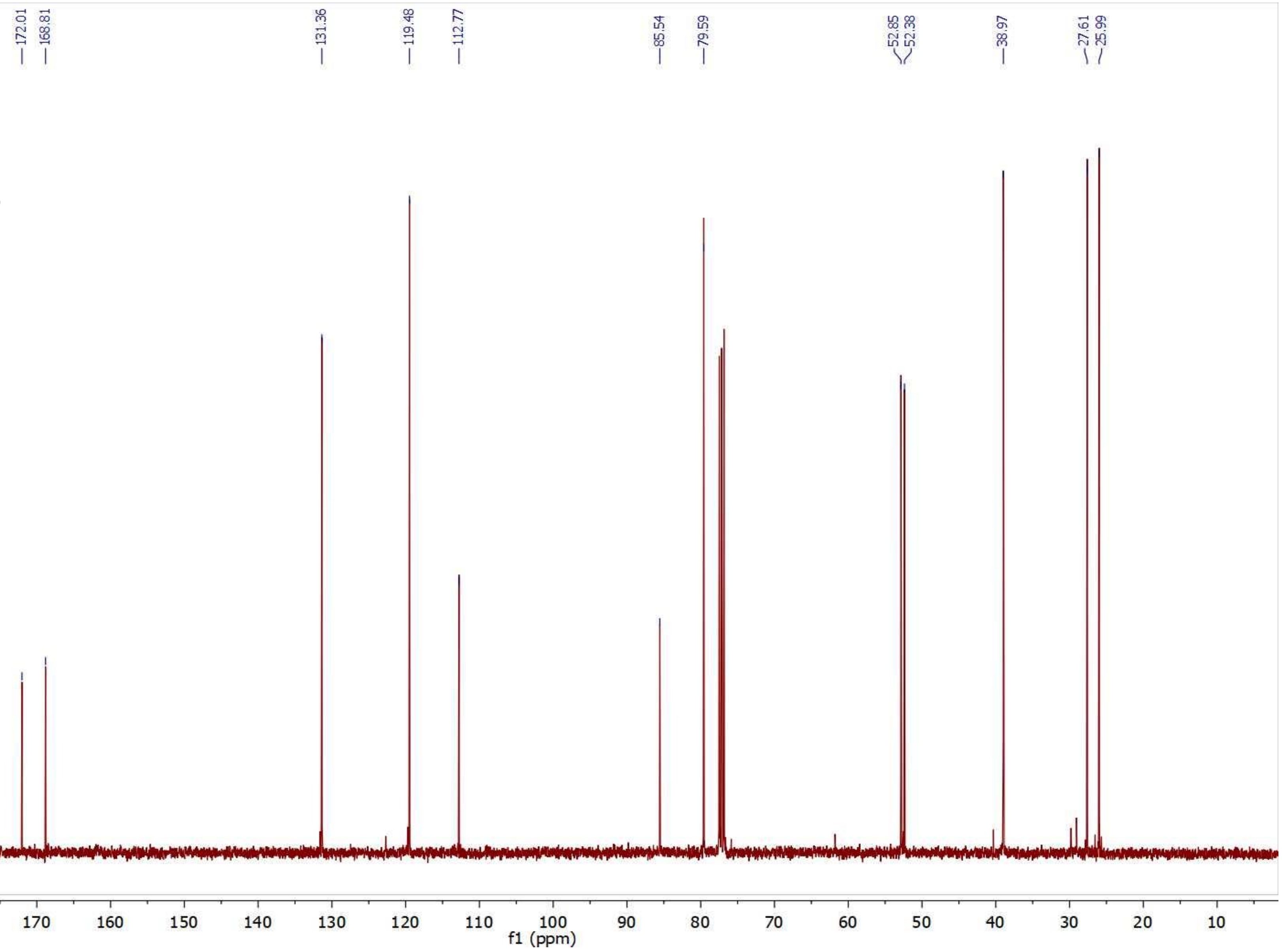

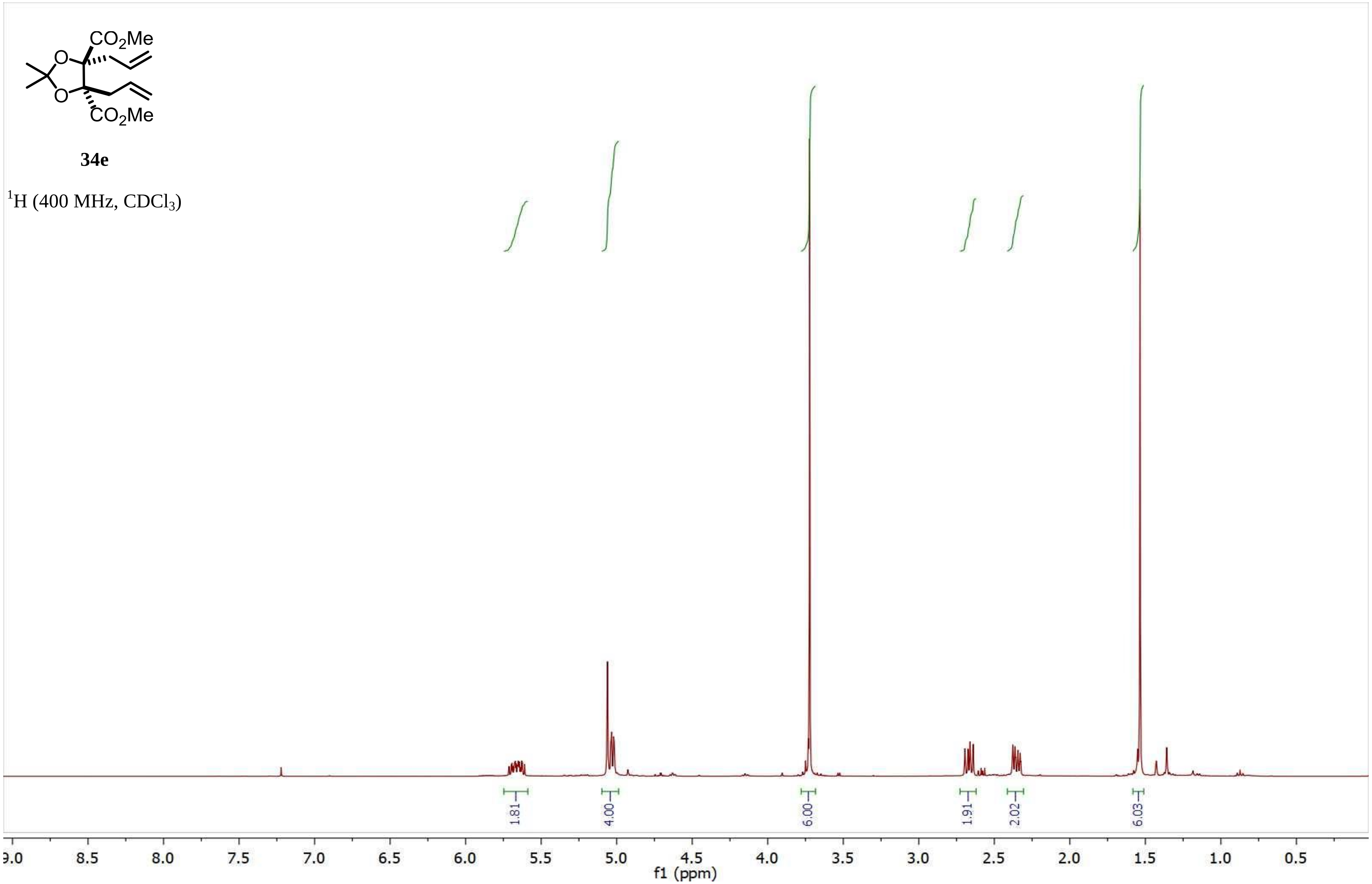

CO2Me
O
O
CO2Me
34e
1H (400 MHz, CDCl3)
1.81
4.00
6.00
1.91
2.02
6.03
9.0 8.5 8.0 7.5 7.0 6.5 6.0 5.5 5.0 4.5 4.0 3.5 3.0 2.5 2.0 1.5 1.0 0.5
f1 (ppm)

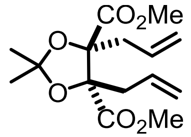

34e

^{13}C (100 MHz, $CDCl_3$)

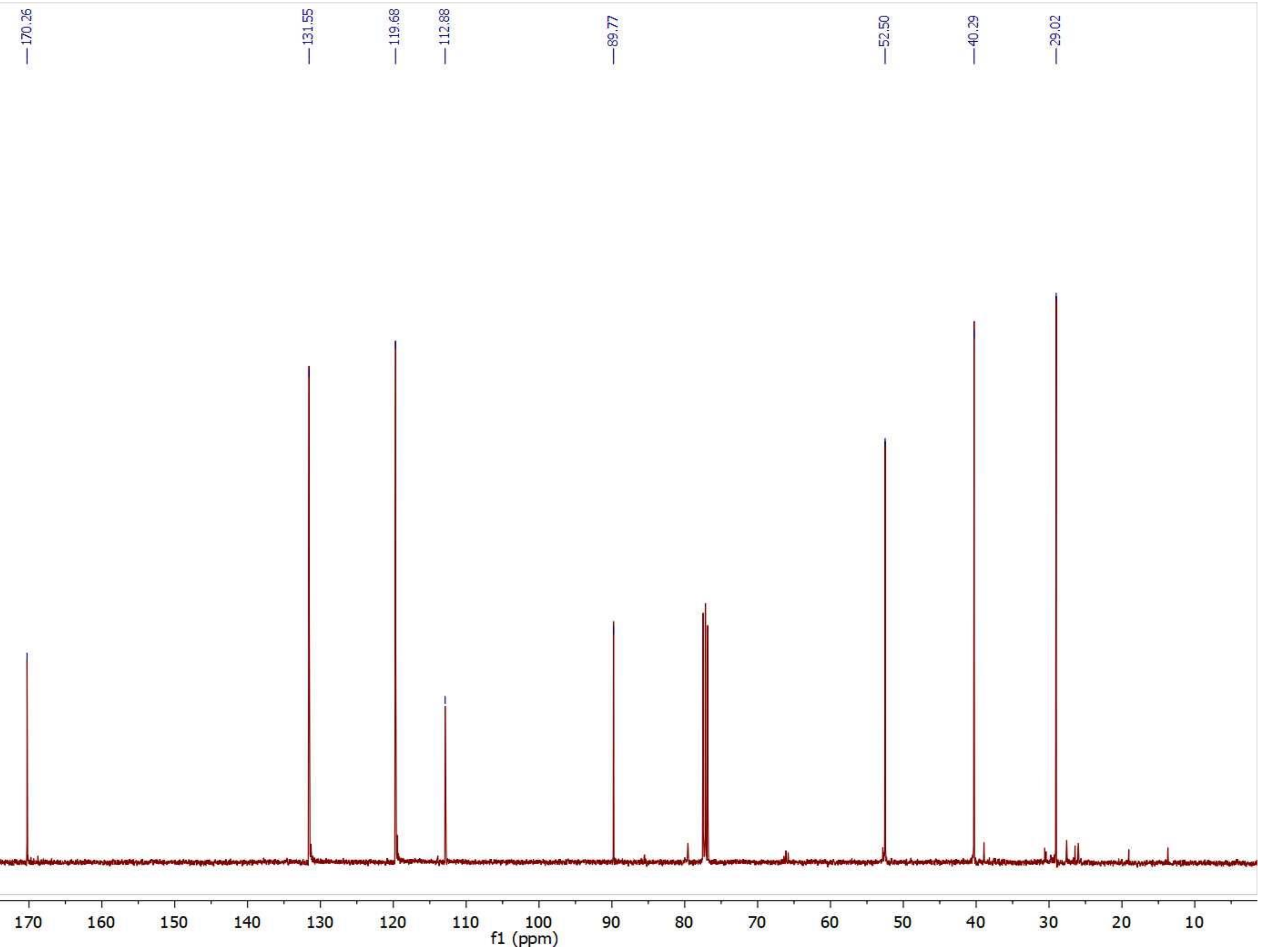

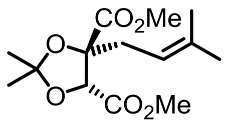

33f

^{1}H (400 MHz, $CDCl_3$)

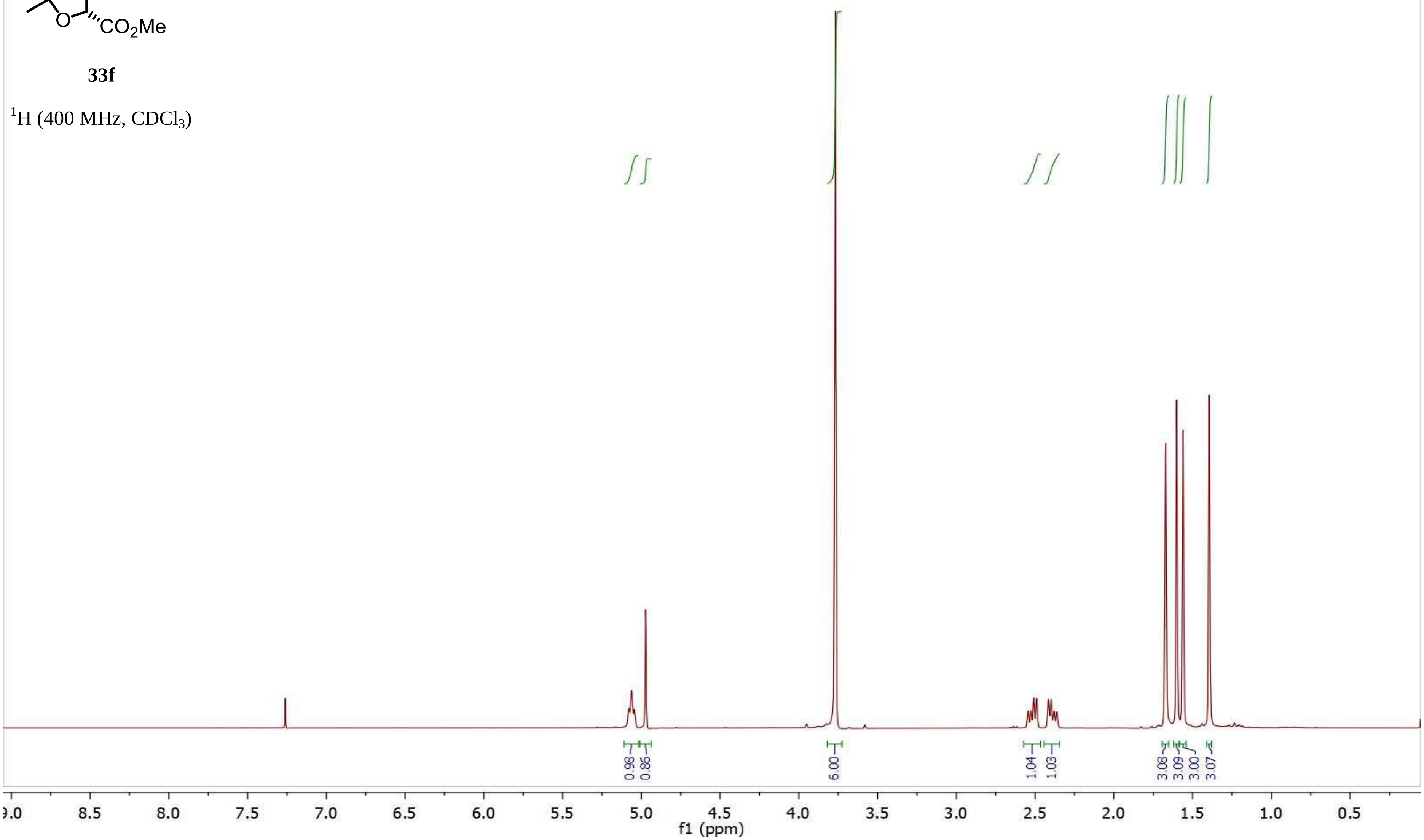

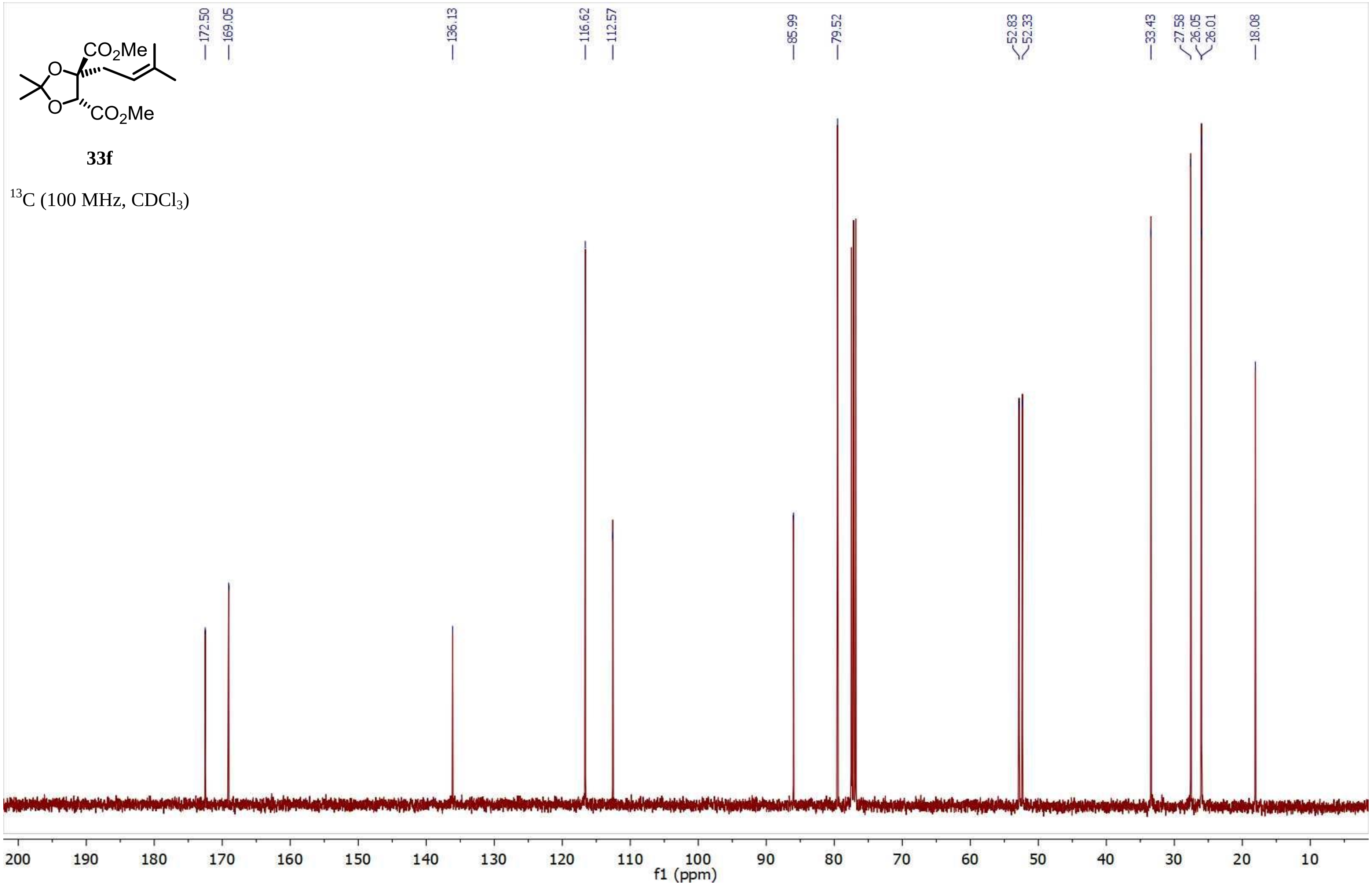
CO2Me
CO2Me
33f
13C (100 MHz, CDCl3)
172.50
169.05
136.13
116.62
112.57
85.99
79.52
52.83
52.33
33.43
27.58
26.05
26.01
18.08
200 190 180 170 160 150 140 130 120 110 100 90 80 70 60 50 40 30 20 10
f1 (ppm)

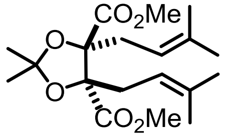

34f

^{1}H (400 MHz, $CDCl_3$)

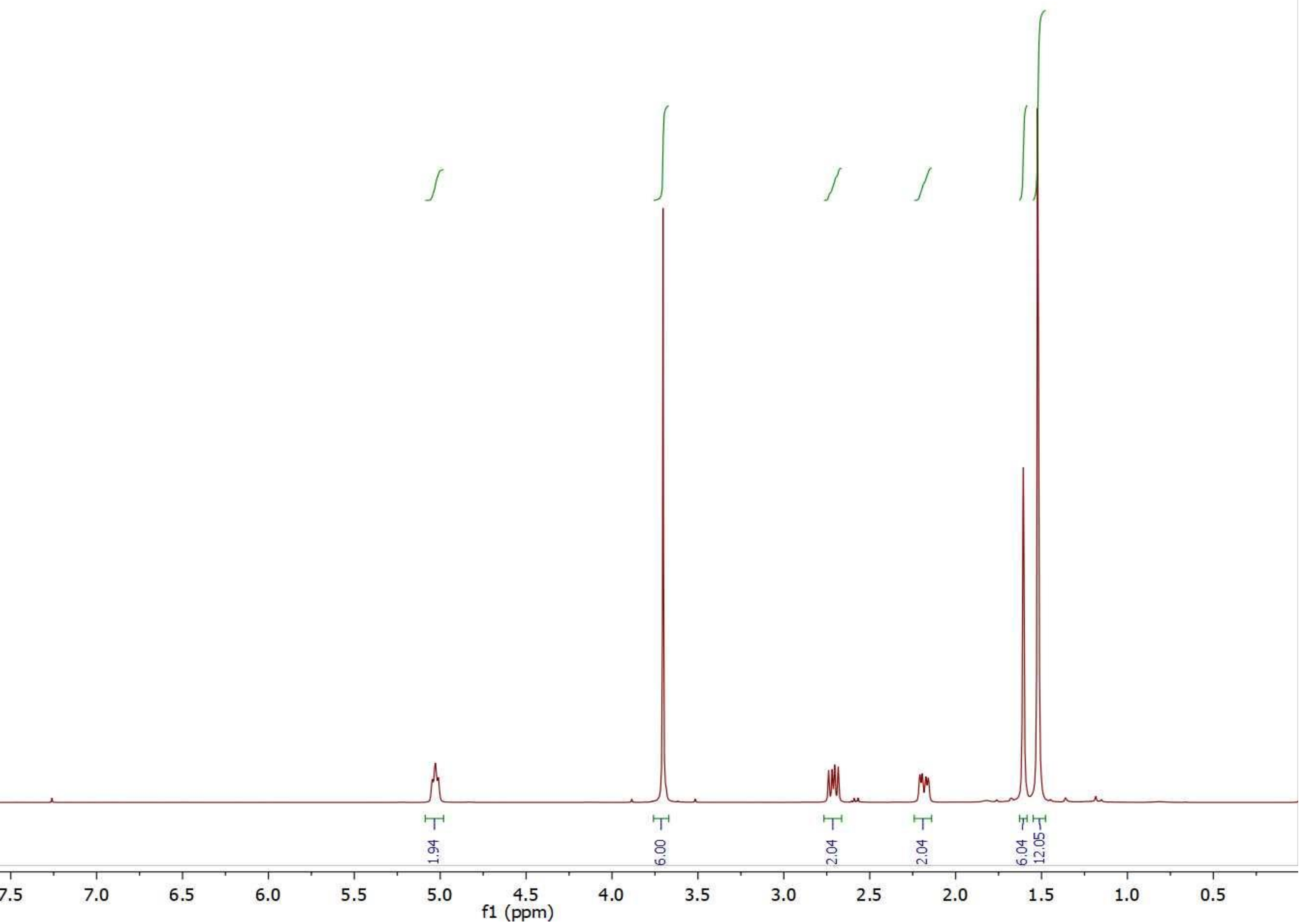

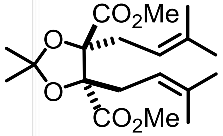

34f

^{13}C (100 MHz, $CDCl_3$)

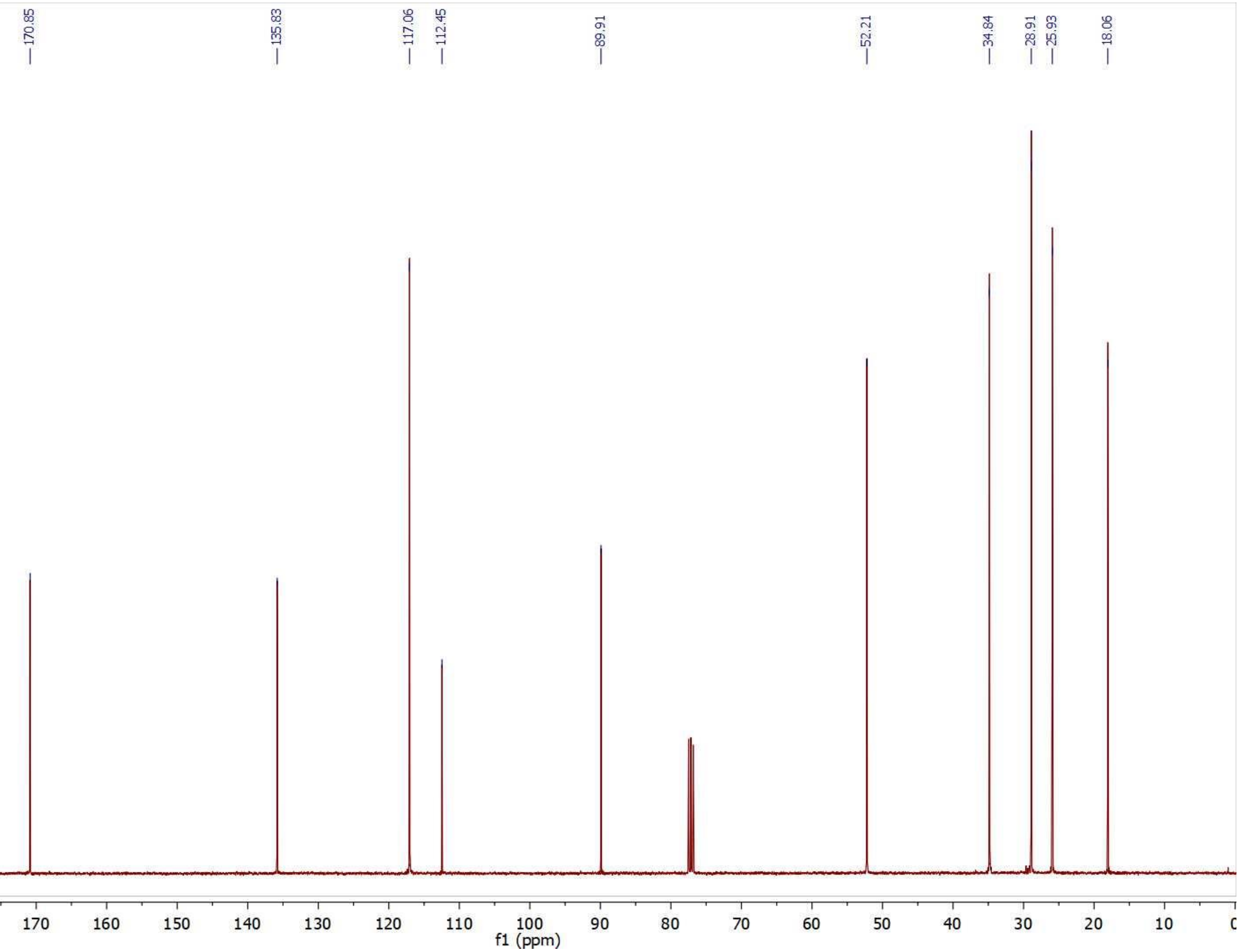

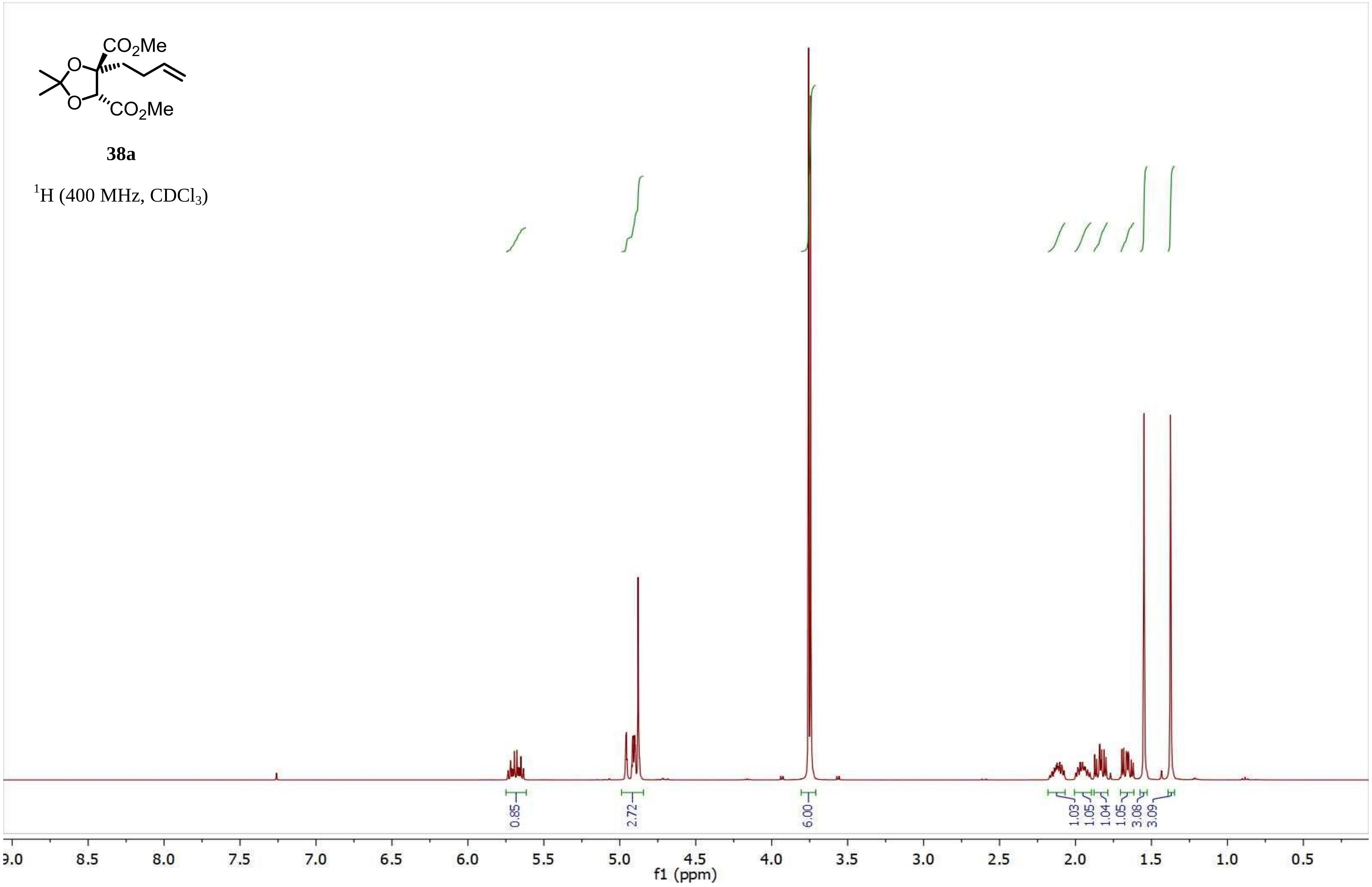
CO2Me
O
O
CO2Me
38a
1H (400 MHz, CDCl3)
0.85
2.72
6.00
1.03
1.05
1.04
1.05
3.08
3.09
9.0 8.5 8.0 7.5 7.0 6.5 6.0 5.5 5.0 4.5 4.0 3.5 3.0 2.5 2.0 1.5 1.0 0.5
f1 (ppm)

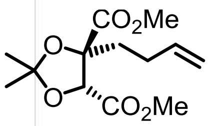

38a

^{13}C (100 MHz, $CDCl_3$)

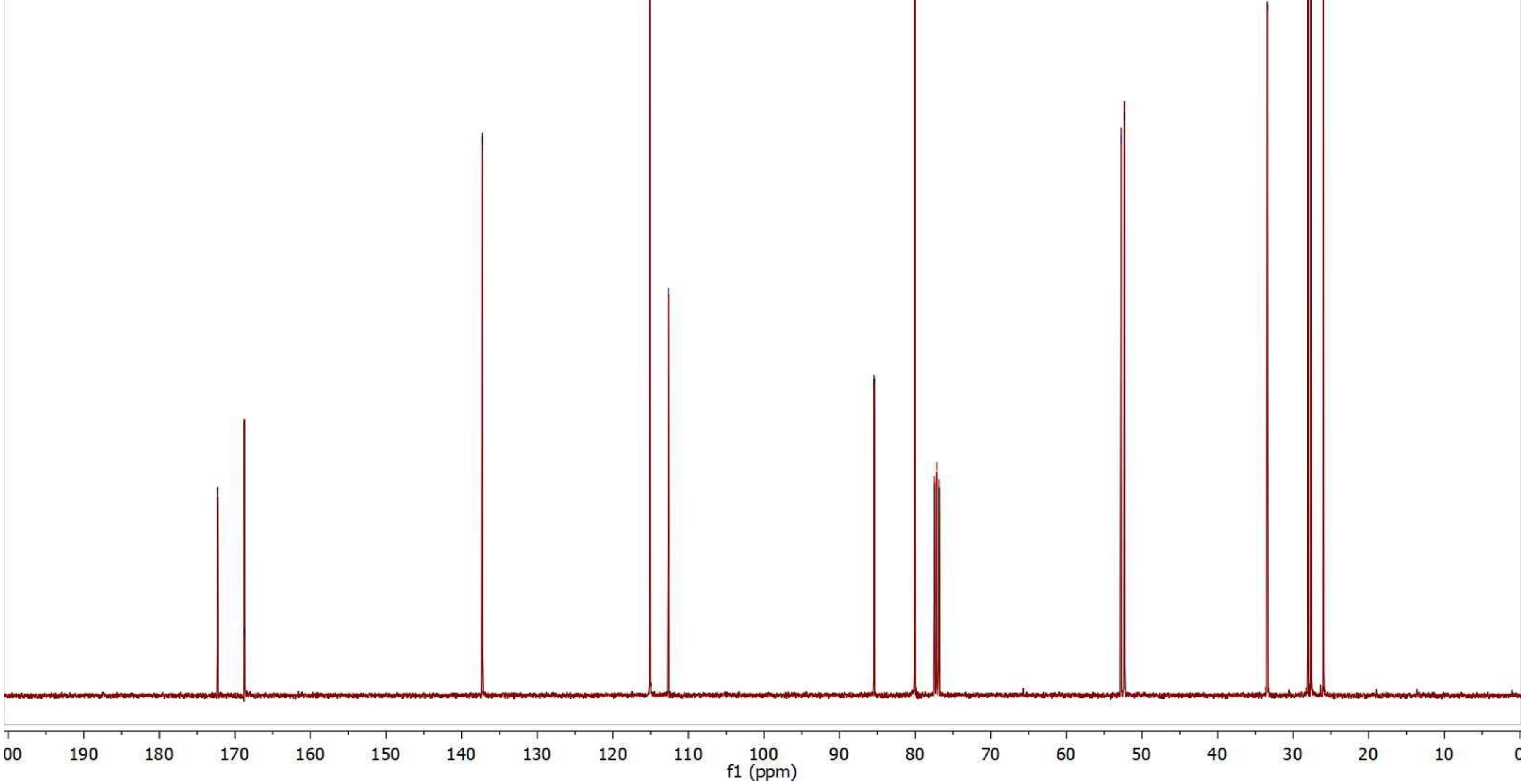

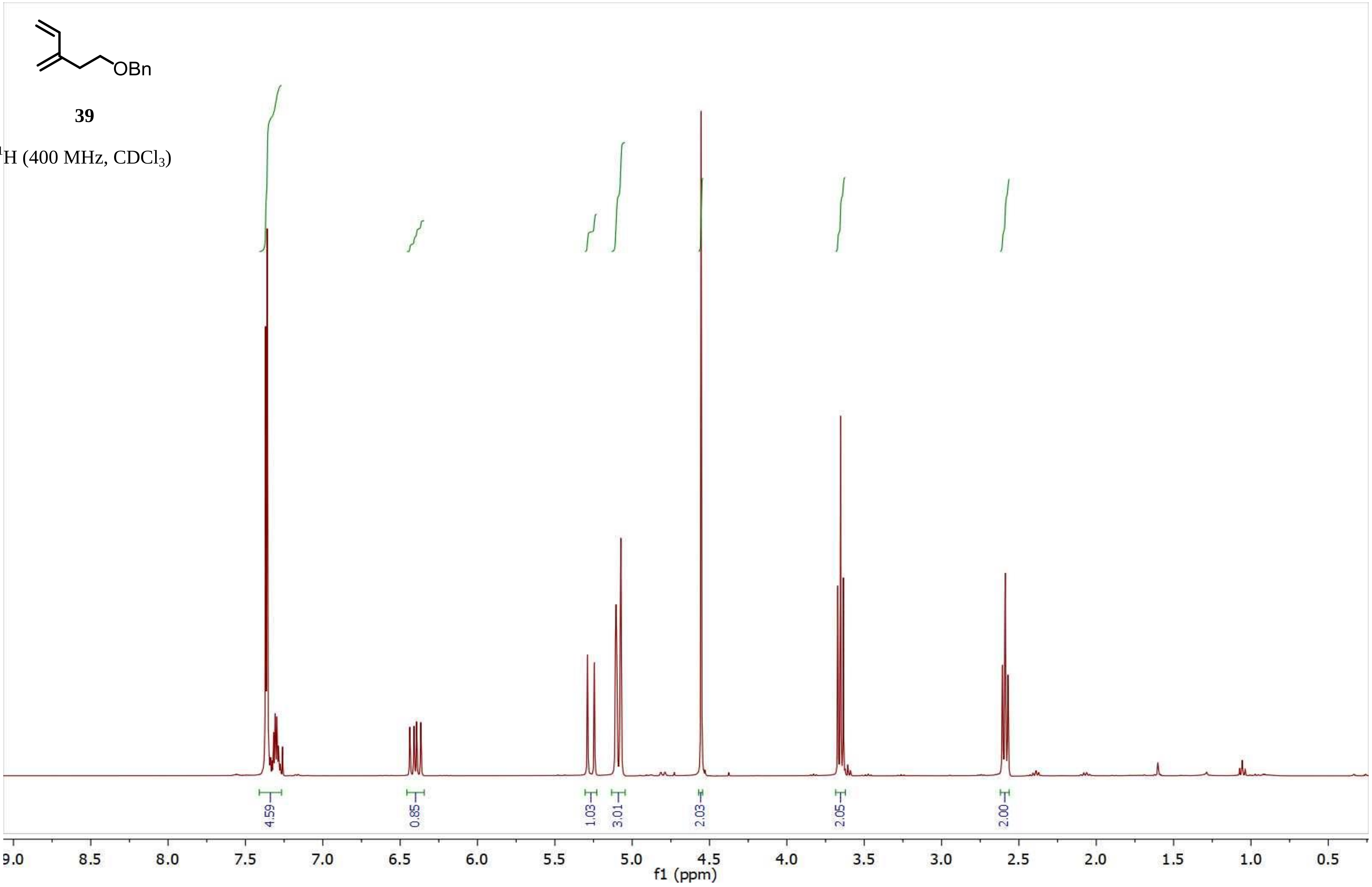

39

^{1}H (400 MHz, $CDCl_3$)

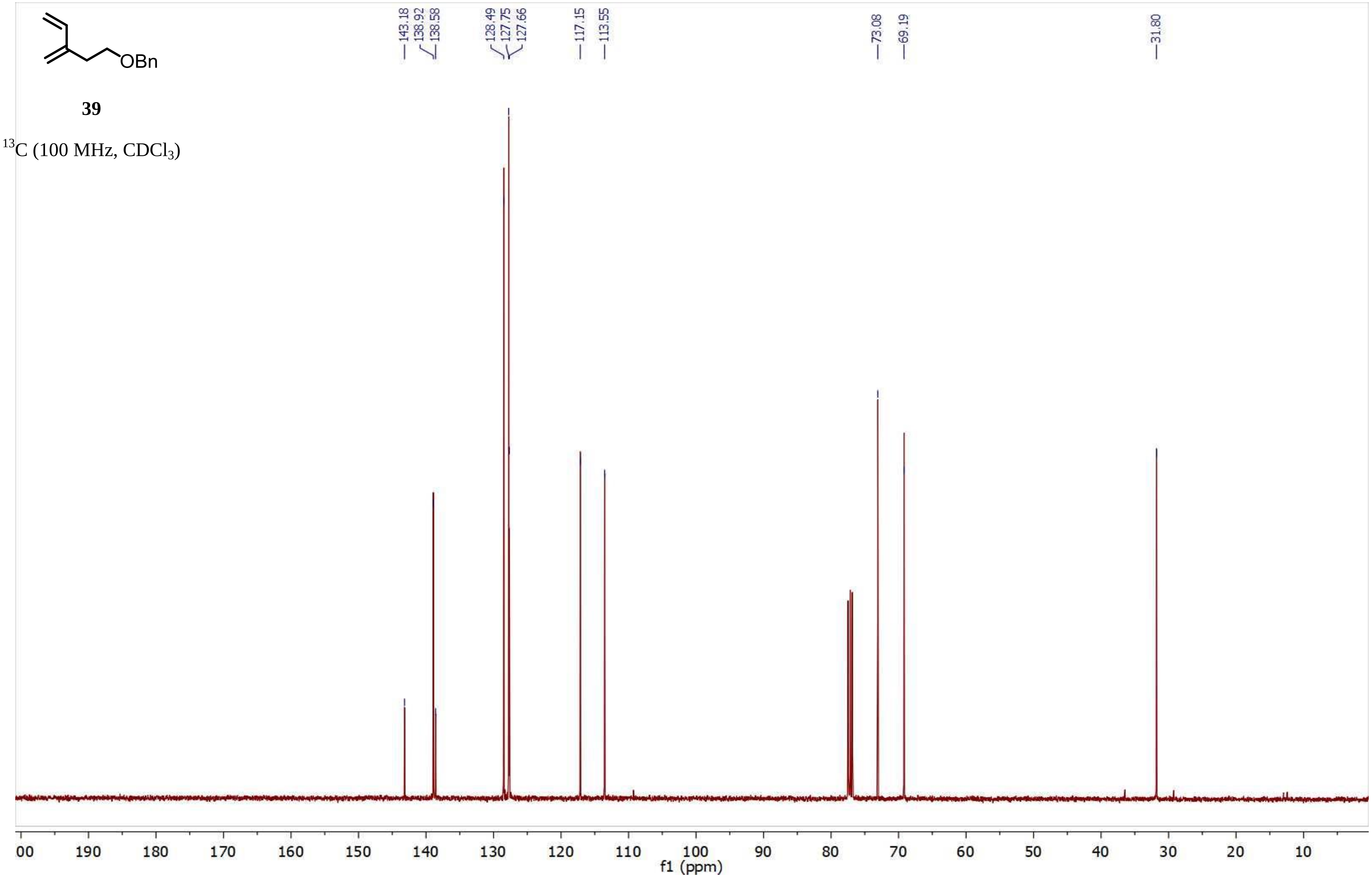
OBn
39
13C (100 MHz, CDCl3)
143.18
138.92
138.58
128.49
127.75
127.66
117.15
113.55
73.08
69.19
31.80
00
190
180
170
160
150
140
130
120
110
100
90
80
70
60
50
40
30
20
10
f1 (ppm)

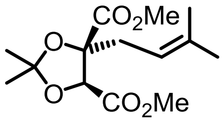

***epi*-33f**

^{1}H (400 MHz, $CDCl_3$)

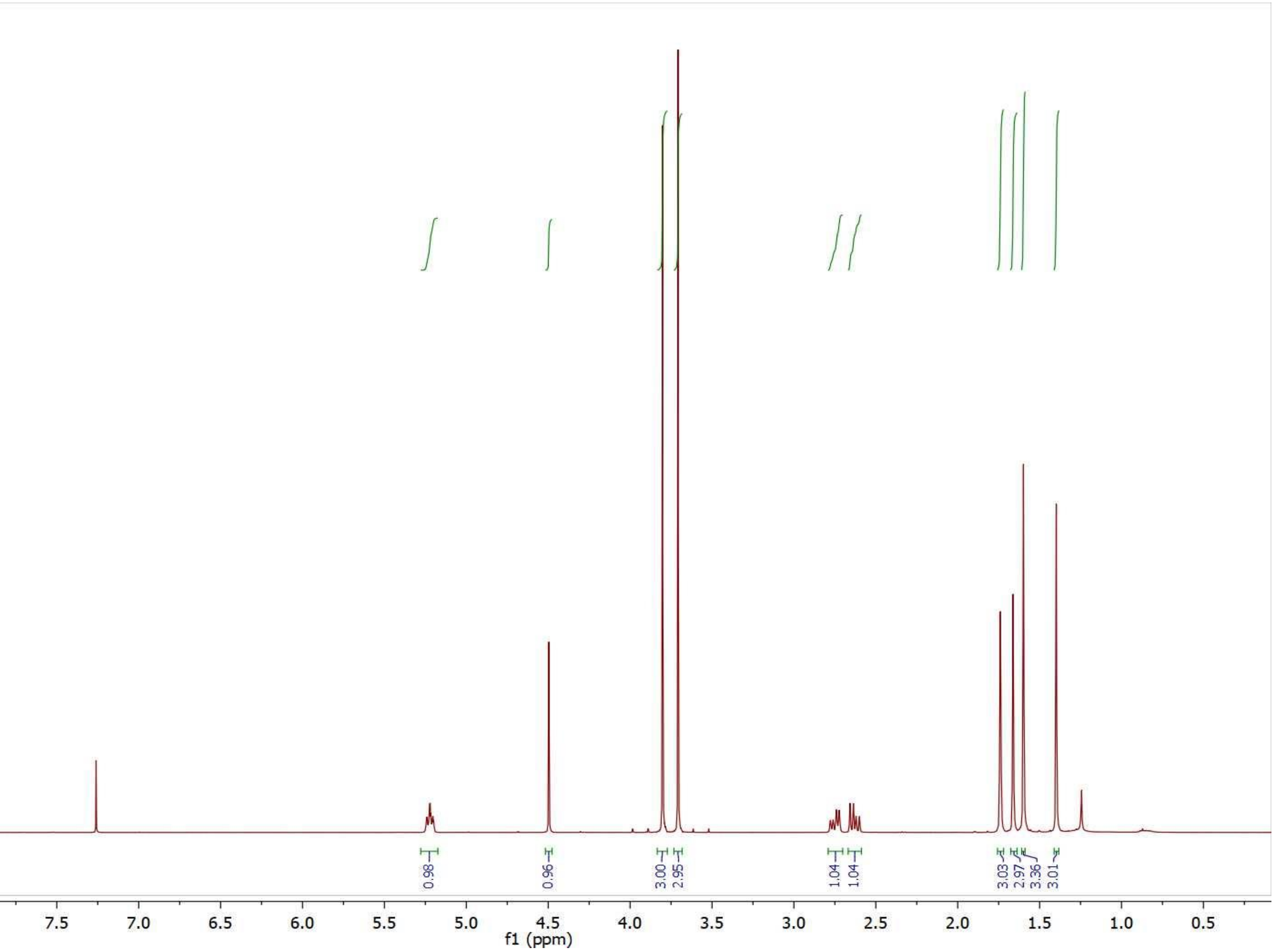

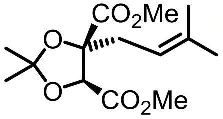

***epi*-33f**

^{13}C (100 MHz, $CDCl_3$)

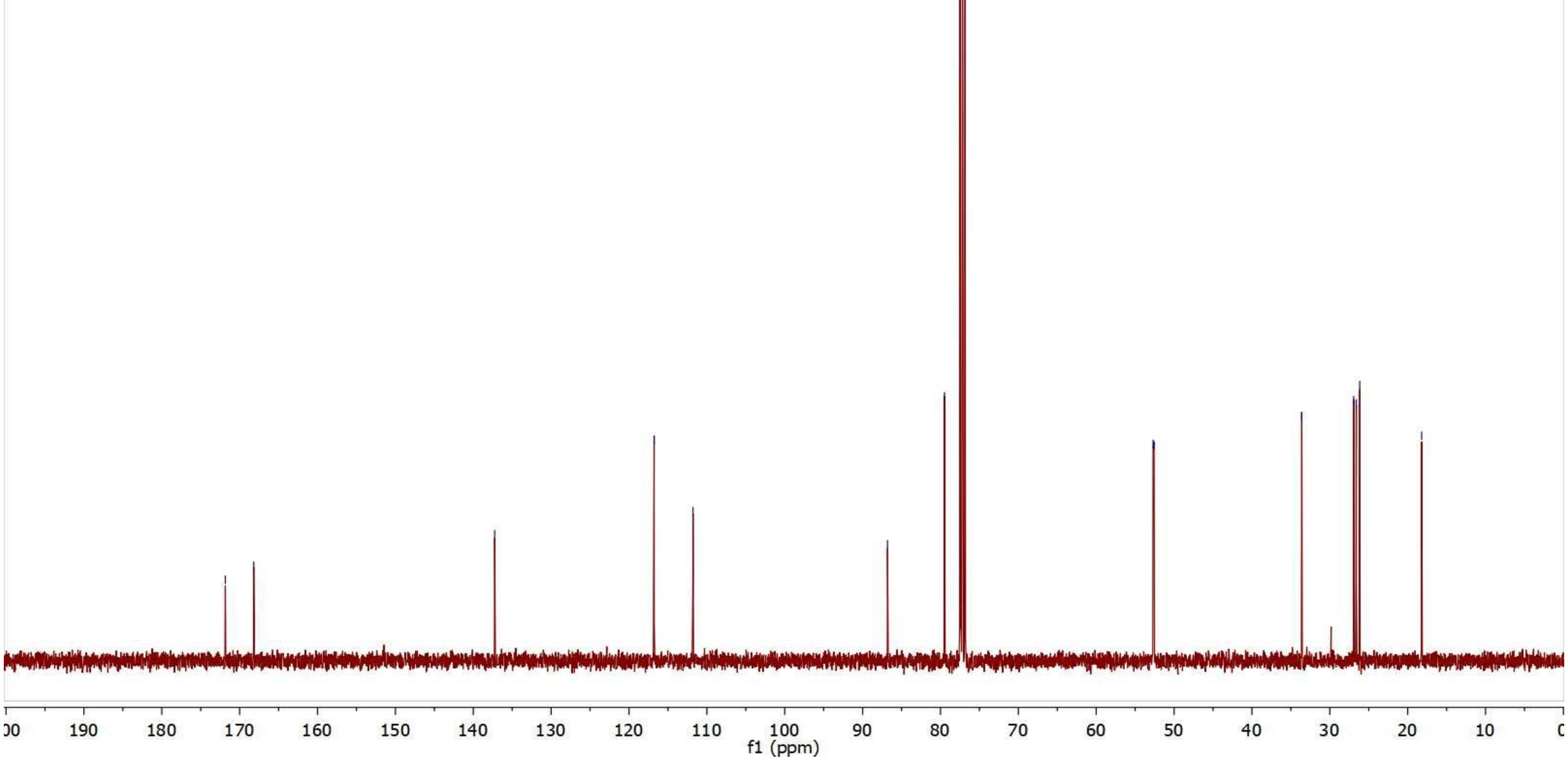

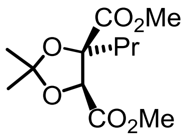

***epi*-33a**

^{1}H (400 MHz, $CDCl_3$)

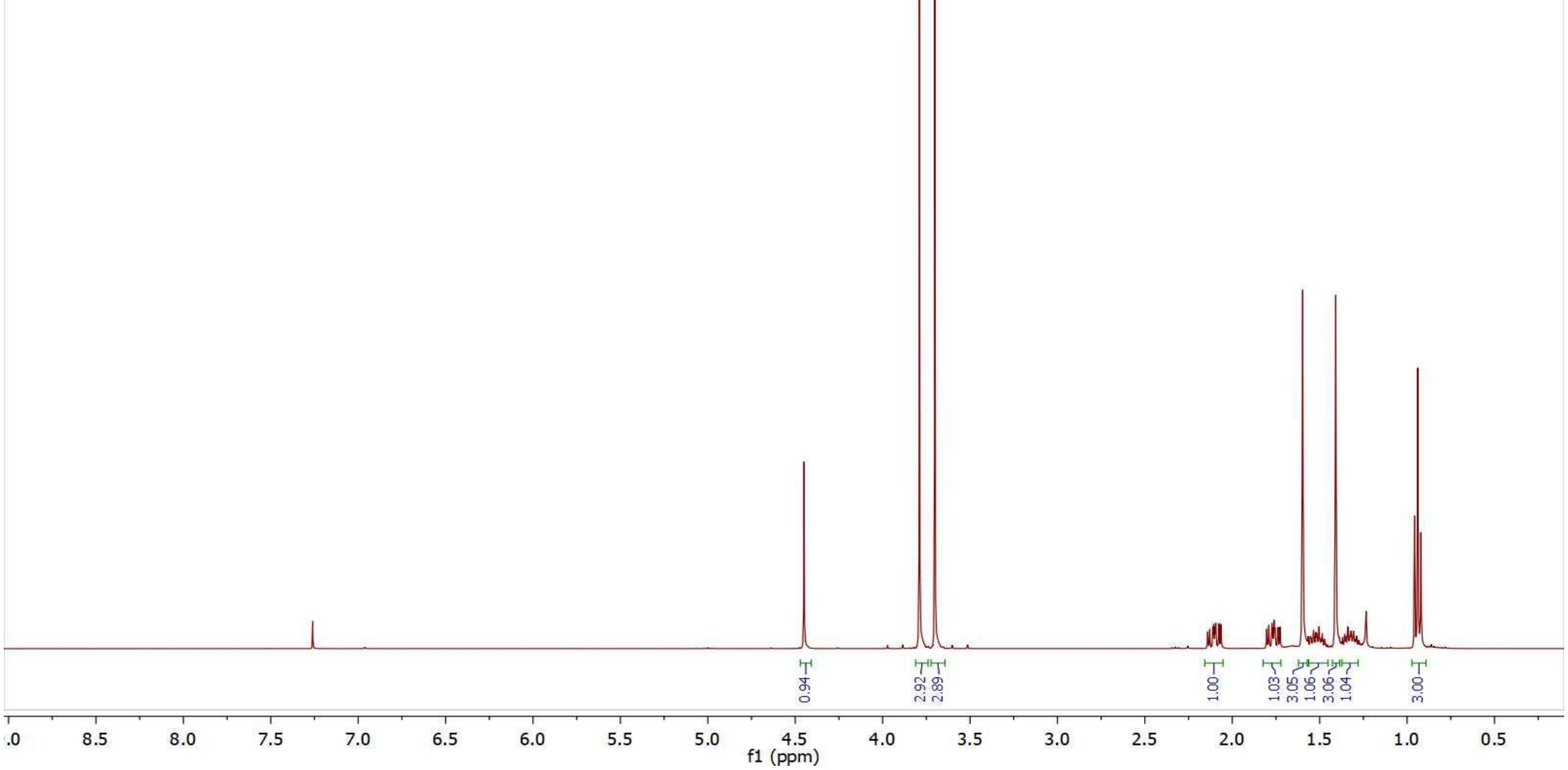

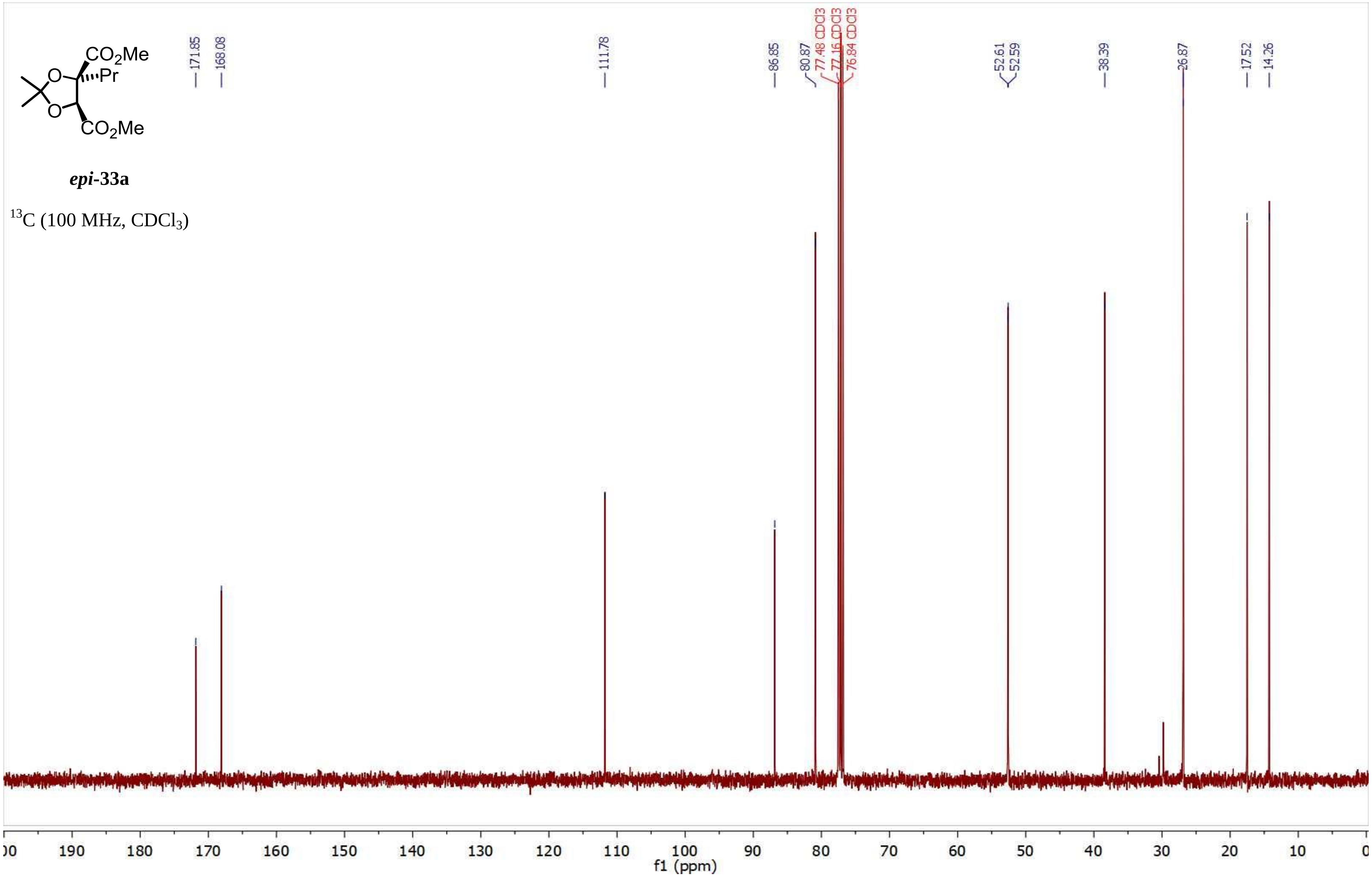
CO2Me
Pr
O
O
CO2Me
epi-33a
13C (100 MHz, CDCl3)
171.85
168.08
111.78
86.85
80.87
77.48 CDCl3
77.16 CDCl3
76.84 CDCl3
52.61
52.59
38.39
26.87
17.52
14.26
f1 (ppm)

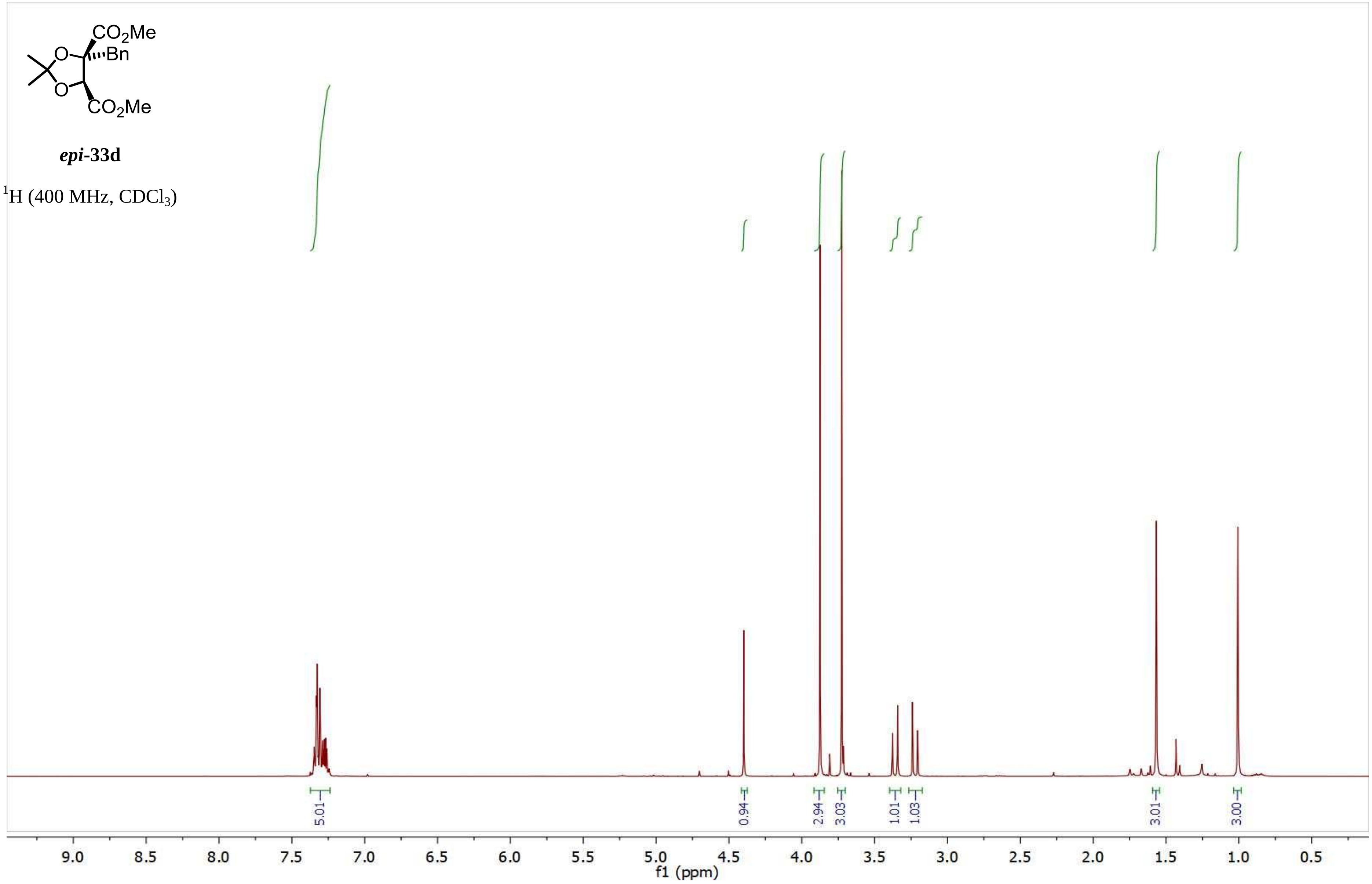

***epi*-33d**

^{1}H (400 MHz, $CDCl_3$)

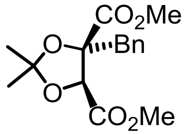

***epi*-33d**

^{13}C (100 MHz, $CDCl_3$)

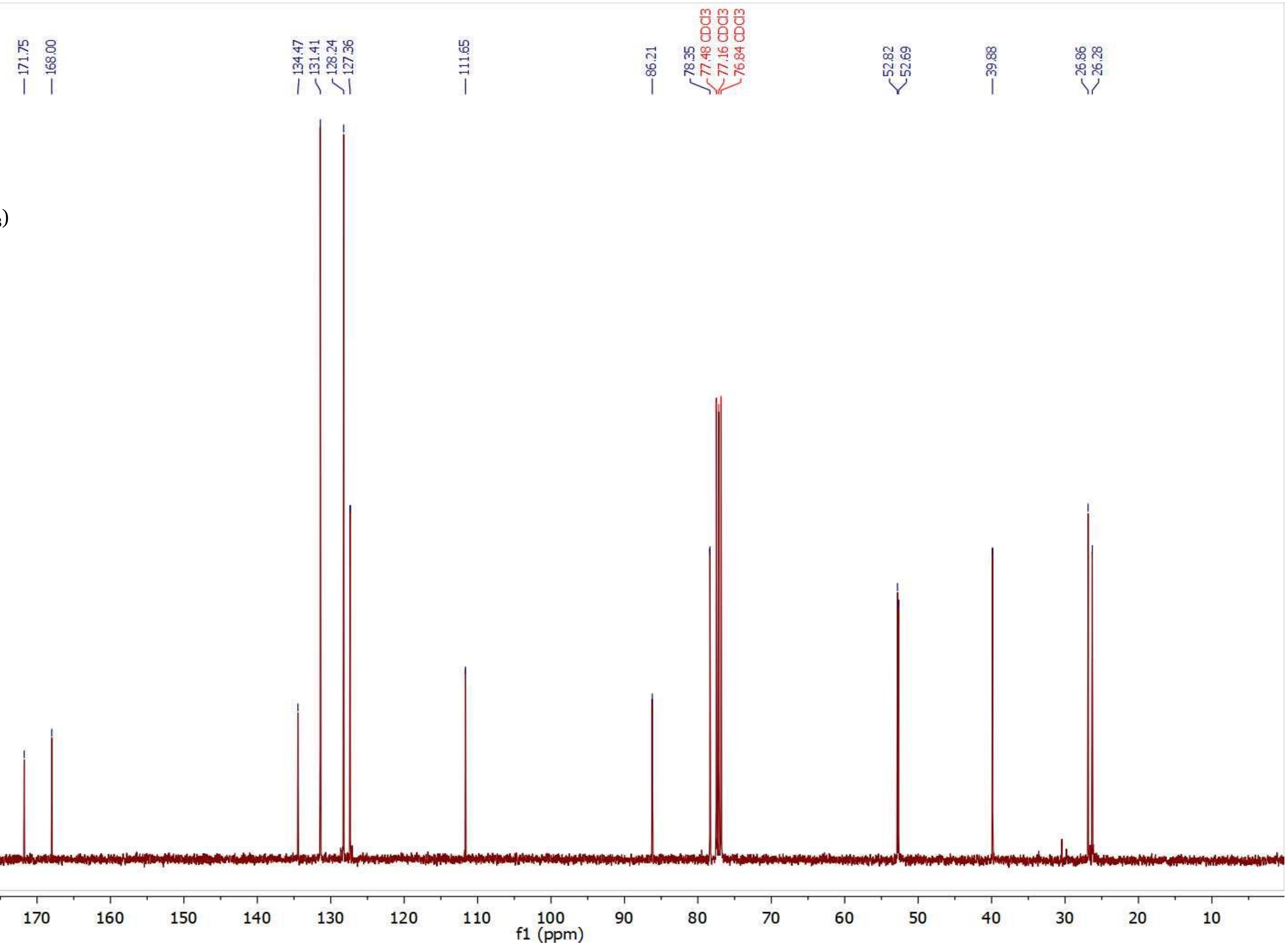

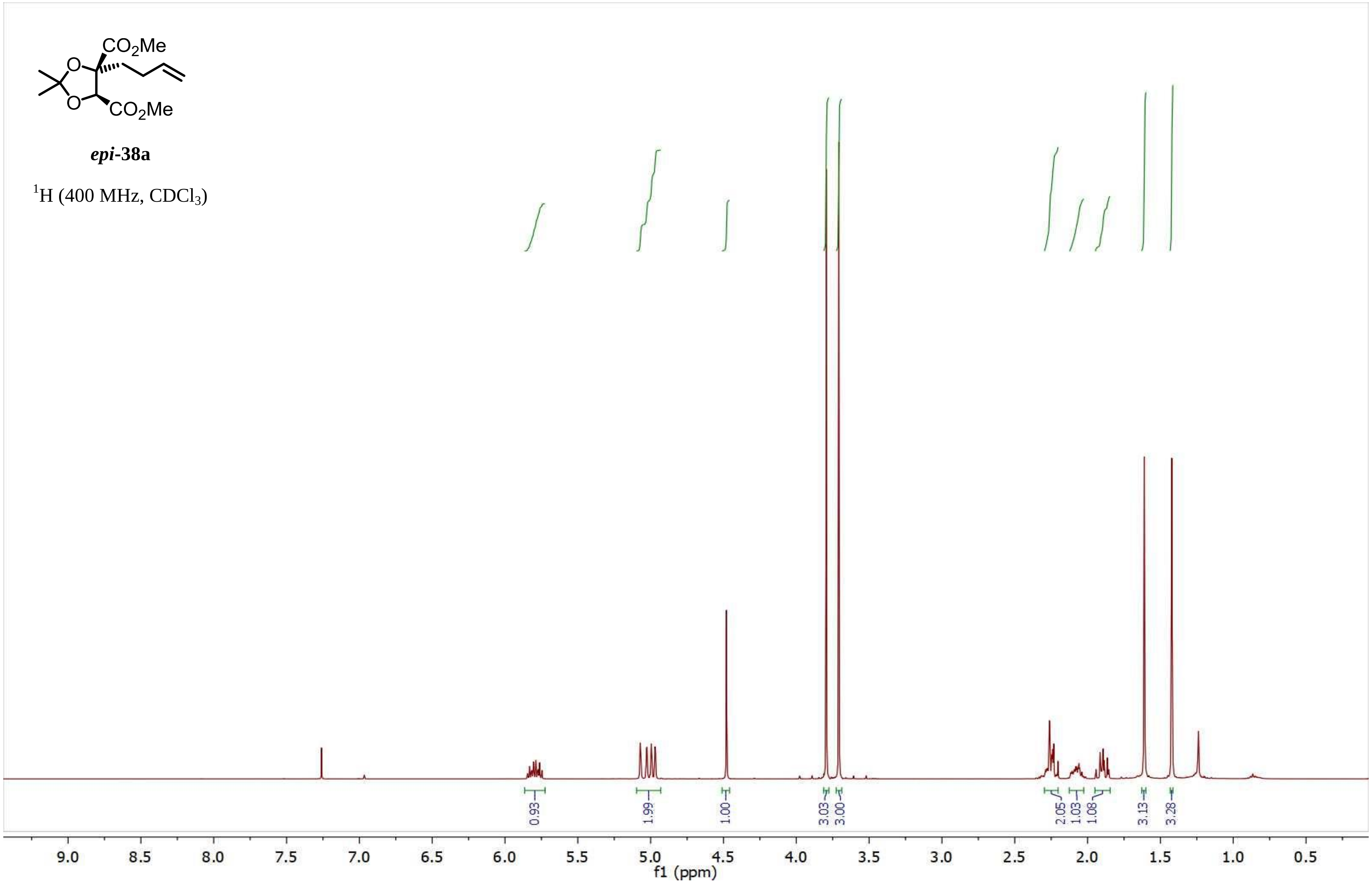
CO2Me
O
O
CO2Me
epi-38a
1H (400 MHz, CDCl3)
0.93
1.99
1.00
3.03
3.00
2.05
1.03
1.08
3.13
3.28
9.0 8.5 8.0 7.5 7.0 6.5 6.0 5.5 5.0 4.5 4.0 3.5 3.0 2.5 2.0 1.5 1.0 0.5
f1 (ppm)

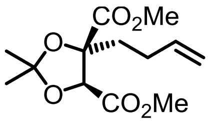

***epi*-38a**

^{13}C (100 MHz, $CDCl_3$)

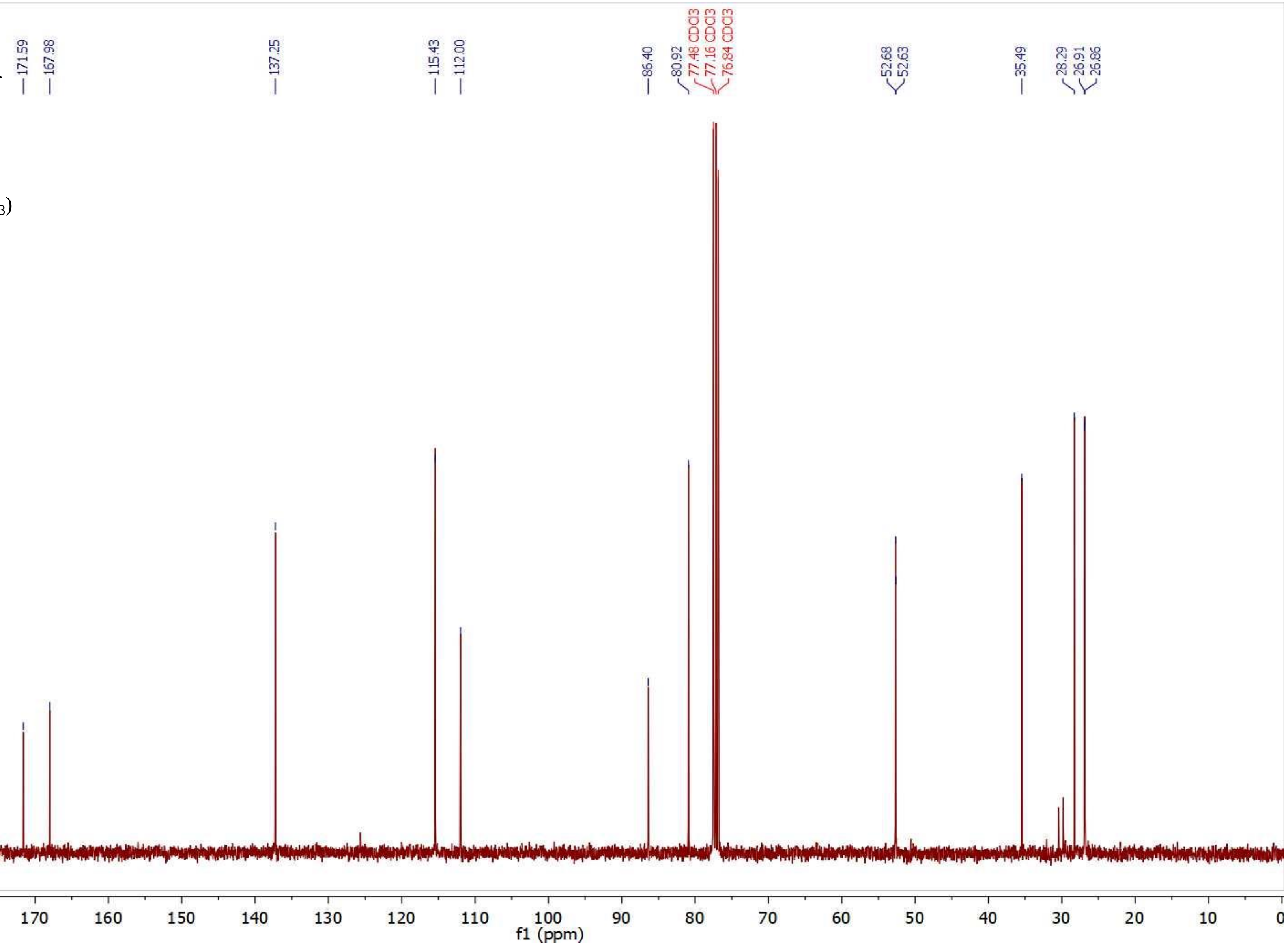

Supplement: File 1 — Experimental procedures, characterisation data and 1H and 13C NMR spectra for all new compounds. [file Beilstein_J_Org_Chem-15-1194-s001.pdf]
